# Safety and feasibility of oral zinc for patients with *GNAO1*-related disorders (ZINCGNAO1): an open-label, single-arm, single-centre, pilot trial in Germany

**DOI:** 10.1016/j.eclinm.2026.104092

**Published:** 2026-07-23

**Authors:** Moritz Thiel, Kyriakos Martakis, Ibrahim Duran, Hormos S. Dafsari, Petra Schiller, Jithmi Weliwitage, Barbara Hero, Yonika Larasati, Alexey Koval, Vladimir L. Katanaev, Anne Koy

**Affiliations:** a Department of Paediatrics, Faculty of Medicine and University Hospital Cologne, University of Cologne, Cologne, Germany; b Center for Rare Diseases, Faculty of Medicine and University Hospital Cologne, University of Cologne, Cologne, Germany; c Department of Child Neurology, Justus-Liebig University Gießen, Gießen, Germany; d Third Department of Paediatrics, ‘Attikon’ University General Hospital, Medical School, National and Kapodistrian University of Athens, Athens, Attica, Greece; e University of Cologne, Medical Faculty and University Hospital, Center of Prevention and Rehabilitation, UniReha, Germany; f Max-Planck-Institute for Biology of Ageing, Cologne Excellence Cluster on Cellular Stress Responses in Aging Associated Diseases (CECAD), Cologne, Germany; g Institute of Medical Statistics and Computational Biology (IMSB), Faculty of Medicine and University Hospital Cologne, University of Cologne, Cologne, Germany; h Department of Paediatric Oncology and Hematology, University Children's Hospital of Cologne, Medical Faculty, Cologne, Germany; i Translational Research Center in Oncohaematology, Department of Cell Physiology and Metabolism, Faculty of Medicine, University of Geneva, Geneva, Switzerland; j Qatar Biomedical Research Institute, Hamad Bin Khalifa University, Doha, Qatar

**Keywords:** GNAO1-related disorder, Zinc acetate, Safety and feasibility, Paediatric movement disorders, Developmental and epileptic encephalopathy, Precision medicine

## Abstract

**Background:**

*GNAO1*-related disorders (*GNAO1*-RD), caused by variants in *GNAO1* encoding the Gαo protein, comprise a broad phenotypic spectrum including movement disorders (MD) and/or epilepsy and are typically associated with developmental delay and intellectual disability. Currently, only symptomatic treatments are available. Zinc can restore guanosine triphosphate hydrolysis and cellular interactions of dysfunctional Gαo. ZINCGNAO1 evaluated the safety and feasibility of oral zinc supplementation in *GNAO1*-RD.

**Methods:**

ZINCGNAO1 was a 6-month, open-label, fixed-dose, single-centre pilot trial in Germany. Eligible participants were aged 6 months to 30 years, had a genetically confirmed *GNAO1* variant, at least one hallmark feature of GNAO1-RD (MD, hypotonia, epilepsy, or global developmental delay), and had a Gross Motor Function Measure-66 (GMFM-66) score of 75 or lower. Zinc acetate dihydrate was administered orally in age-dependent dosages. Primary outcomes were safety, assessed by adverse event monitoring and laboratory analyses, and feasibility, defined as treatment adherence of at least 80% of days. Exploratory secondary outcomes included changes in MD severity (Burke-Fahn-Marsden Dystonia Rating Scale-Movement Score, BFMDRS-M, Abnormal involuntary movement scale, AIMS), and related disability (BFMDRS-Disability Score), motor function (GMFM-66), quality of life (Caregiver Priorities and Child Health Index of Life with Disabilities, CPCHILD), occupational performance (Canadian Occupational Performance Measure performance and satisfaction scores, COPM-P/-S), epilepsy, and participant- or caregiver-reported outcomes. This trial was registered at ClinicalTrials.gov (NCT06412653) and the EU Clinical Trials Register (2024-512735-72-00) and is complete.

**Findings:**

Between Aug 2, 2024, and Jan 27, 2025, 13 participants with 11 different genetic variants were enrolled; 11 completed the follow up of the trial. Mean age at inclusion was 8.0 years (range 0.5–25.1). Overall treatment feasibility was 84.6% (11/13). No treatment-related serious adverse events occurred, and laboratory monitoring did not identify clinically relevant safety concerns during the 6-month treatment period. Exploratory secondary outcomes showed no change in MD severity. The mean BFMDRS Disability Score decreased from 25.5 (SD 2.6) to 23.5 (SD 2.8; nominal p = 0.005). Mean GMFM-66 increased from 33.1 (SD 12.8) to 37.8 (SD 12.8, nominal p = 0.006). COPM-P increased from 2.6 (SD 0.8) to 3.8 (SD 1.3; nominal p = 0.003), and COPM-S from 2.7 (SD 0.9) to 4.1 (SD 1.7; nominal p = 0.002). CPCHILD and seizure occurrence showed no clear treatment-related change; two participants had seizures during the trial.

**Interpretation:**

Our preliminary findings show that oral zinc supplementation was safe and feasible in this *GNAO1*-RD population. Exploratory secondary outcomes suggested possible improvements in motor function and daily activities. Larger controlled trials are needed to assess efficacy.

**Funding:**

*GNAO1*-Gemeinsam nicht allein e.V.

**Research in context**

**Evidence before this study**

Evidence on *GNAO1*-related disorders (*GNAO1*-RD) is scarce. Before recruitment was initiated, we searched PubMed/MEDLINE from database inception to June 25, 2024, without language restrictions, using the terms “GNAO1”, “GNAO1-related disorder”, “GNAO1-associated disorder”, “GNAO1 encephalopathy”, “GNAO1 movement disorder”, “zinc”, “zinc acetate”, “treatment”, and “clinical trial”. A broad GNAO1 search yielded 197 records; a treatment-focused search yielded 68 records; a zinc-focused search yielded two records, both related to preclinical work forming the rationale for the present study; and a clinical trial-focused search yielded two records, neither of which reported a completed interventional trial of a disease-modifying therapy in a GNAO1-RD cohort. The available clinical literature consisted mainly of single case reports, retrospective cohort studies, and larger case series. Disease mechanisms had been investigated using complementary experimental approaches, including computational modelling, cell-based assays, and animal models such as *Caenorhabditis elegans*, *Drosophila melanogaster*, and mice. We also searched ClinicalTrials.gov and the EU Clinical Trials Register on June 25, 2024, using the term “GNAO1”; apart from the present trial, registered studies were observational natural history or registry studies, and no completed interventional treatment trial was identified. Zinc had shown efficacy *in vitro* and in vivo in Drosophila, and safety had been shown in a mouse model. The use of zinc in paediatric patients had been limited to other disorders, including high-dose treatment for Wilson disease from the age of 1 year and above.

**Added value of this study**

To date, no disease-modifying therapy has been evaluated in interventional clinical trials for *GNAO1*-RD. This first-in-human pilot trial in paediatric patients and young adults with *GNAO1*-RD demonstrates that oral zinc supplementation is feasible and well tolerated. Exploratory secondary outcomes provide data suggesting improvements in motor function and occupational performance, but these findings should be interpreted cautiously. The study highlights the importance of structured, standardised outcome measures in this rare and clinically heterogeneous disorder.

**Implications of all the available evidence**

With no approved pharmacological treatment for patients with *GNAO1*-RD to date, the present findings support further clinical evaluation of zinc in this complex paediatric neurological disorder. The study design and outcome measures set the ground for future clinical trials on larger cohorts of patients with *GNAO1*-RD, facilitating the evaluation of additional disease-modifying therapies. Limitations related to the zinc formulation applied in this study underscore the need for further drug development efforts, including the assessment of alternative formulations and dosing strategies.

## Introduction

*GNAO1*-related disorders (*GNAO1*-RD) constitute a group of dominantly inherited neurological disorders characterised by marked genetic and phenotypic heterogeneity. They are caused predominantly by de novo variants in the *GNAO1* gene, which encodes the α-subunit of the guanosine nucleotide-binding Go protein (Gαo). Gαo is among the most abundant proteins in the brain^1^ and is a critical mediator of G-protein–coupled receptor (GPCR) signalling, including GABAB, α2A-adrenergic, adenosine A1, and dopamine D2 receptors.^2^

To date, more than 400 affected individuals with over 100 distinct *GNAO1* variants have been reported,^3^ establishing *GNAO1*-RD as an ultra-rare disorder. The clinical presentation varies widely depending on the underlying variant^3^ and typically encompasses a spectrum of hyperkinetic movement disorders (MD) with or without epilepsy, typically associated with global developmental delay and intellectual disability.^4^ Epilepsy manifests either as early-infantile developmental and epileptic encephalopathy syndrome (EIDEE), frequently drug-resistant, or as later-onset epilepsy with heterogeneous seizure types.^5^ The hyperkinetic MD is characterised by dystonia and choreoathetosis interspersed with episodes of hypotonia. A pathognomonic feature of *GNAO1*-RD is the occurrence of severe dyskinetic crises—episodes of nearly continuous involuntary movements or sustained dystonic posturing, often precipitated by infection, voluntary movement, or emotional stress. These crises can be extremely painful and, if unmitigated, may escalate into dyskinetic status, a life-threatening emergency.

The widespread application of comprehensive genetic testing, particularly exome sequencing, has recently broadened the recognised phenotypic spectrum of *GNAO1*-RD, with increasingly milder and late-onset presentations being identified.^6^ Consequently, the true prevalence of *GNAO1*-RD is likely to be substantially underestimated.

Therapeutic options for *GNAO1*-RD remain limited and targeted to the alleviation of symptoms. Recent insights into the molecular defects of Gαo resulting from pathogenic *GNAO1* variants have identified zinc (Zn^2+^) as a potential precision-medicine approach with variant-specific targeting.^7^ *In vitro* Zn^2+^ substitutes for Mg^2+^ in the active center of the dysfunctional Gαo protein, triggering structural rearrangements that restore GTPase activity and cellular interactions without influencing wild-type Gαo. In a *Drosophila* model, Zn^2+^ supplementation improved motor function and lifespan.^7^ Building on these preclinical findings, we have treated a single patient with the G203R mutation, which yielded substantial clinical improvement.^8^ In the absence of further case reports or extensive natural history study data in GNAO1-RD, these observations provided the rationale for the present pilot trial addressing safety and feasibility of zinc in GNAO1-RD.

## Methods

### Study design and ethics

ZINCGNAO1 was a 6-month, single-centre, open-label, fixed-dose pilot trial evaluating the safety and feasibility of oral zinc in patients with *GNAO1*-RD. Potential participants were recruited by the trial centre's specialised *GNAO1* outpatient clinic, supported by the German patient advocacy organisation “Gemeinsam Nicht allein e.V.”. Participants received 6 months of treatment, with assessments at baseline and at 90 and 180 days (3 and 6 months), supplemented by virtual visits at days 10 and 210 to monitor early safety and post-withdrawal effects. Continuation beyond six months was a pragmatic clinical decision based on perceived benefit or stabilisation in at least one clinically relevant symptom domain, acceptable safety, and caregiver preference; it was not used as a formal efficacy endpoint.

The trial was conducted in accordance with the Declaration of Helsinki, CIOMS guidelines, International Council for Harmonisation–Good Clinical Practice, and applicable regulations. The clinical trial was authorised through the Clinical Trials Information System under Regulation (EU) No 536/2014 by the German competent authority, the Bundesinstitut für Arzneimittel und Medizinprodukte, and received a favourable opinion from the Ethics Committee of Ulm University, which was assigned within the CTIS procedure (trial protocol code Uni-Koeln-5275). The trial was approved under EU CT number 2024-512735-72-00 and was registered at ClinicalTrials.gov (NCT06412653). Written informed consent was obtained from parents or legal guardians. The study protocol is available within the Supplementary Materials.

An independent data monitoring committee (DMC), consisting of three clinicians with expertise in the relevant field and one statistician, oversaw trial conduct and participant safety according to a prespecified DMC charter. The DMC reviewed recruitment, drop-outs, serious adverse events, suspected unexpected serious adverse reactions, unexpected safety signals, and any changes affecting the benefit–risk assessment. The committee was responsible for providing recommendations to the sponsor regarding continuation, modification, or early termination of the trial. An organisational meeting was held at trial initiation, with subsequent reviews scheduled after enrolment of the first participant and thereafter according to recruitment progress and safety needs. Serious adverse events and suspected unexpected serious adverse reactions were to be forwarded to the DMC within one business day, and the DMC chair could request unscheduled meetings if required. During the trial, the DMC reviewed trial conduct and safety data and did not recommend modification or early termination of the study.

### Participants

Eligible participants were aged 6 months to 30 years, carried a pathogenic or likely pathogenic *GNAO1* variant or a variant of uncertain significance, exhibited at least one hallmark feature of *GNAO1*-RD (MD, hypotonia, epilepsy, or global developmental delay), and had a Gross Motor Function Measure (GMFM) score of 75 or lower. Participants were required to be clinically stable on concomitant therapies, including anti-seizure medication, tonus-regulating medication, or deep brain stimulation, for at least three months before enrolment. Because dyskinetic crises are a hallmark manifestation of GNAO1-RD and part of the MD phenotype, their occurrence within this period was not considered an exclusion criterion. Prior zinc treatment within 4 months before inclusion was an exclusion criterion. Full eligibility criteria are provided in the Supplementary Methods. Sex was recorded from medical records and trial documentation as male or female; gender identity was not systematically collected.

### Procedures

Zinc acetate dihydrate (Wilzin 25 mg or 50 mg) was administered orally in age-adjusted doses corresponding to 50–150 mg Zn^2+^ per day (Table 1), starting one day after baseline assessment. Treatment was initiated in an outpatient setting. All *GNAO1* variants were eligible for inclusion.

**Table 1:** Baseline characteristics of participants with *GNAO1*-related disorders.

ID	GNAO1 variant (NM_020988.3)	Sex	Age,months, years	Movement disorder	Epilepsy	GNAO1-RD severity score	Other symptoms	Zinc dose, mg/day	Comments/previous publication
1	c.625C>T (p.R209C)	m	302 25.2	Generalised DY; 4 × DS until DBS; no therapy	Generalised and focal motor onset 7 y; Perampanel; SZ frequency 1/year	5.5 moderate	Anarthria; swallowing impaired; DBS (23.2 y)	3 × 50	4
2	c.863T>C (p.F288S)	f	58 4.8	MH; DY extremities, no therapy; no DC	None	5.5 moderate	Anarthria; swallowing impaired	2 × 25	4
3	c.138A>T (p.K46N)	f	59 4.9	Generalised DY; MH; 2 × DS; frequent DC; Gabapentin; CZP; ClHy	None	10 severe	Incomplete spinal cord injury and paralysis PEG	2 × 25	Drop-out due to non–treatment-related serious adverse event^4^
4	c.736G>A (p.E246K)	f	64 5.3	Generalised DY; CH; MH; DC with infection; 2 × DS, no therapy	None	8 severe	Anarthria, PEG	2 × 25	4
5	c.626G>A (p.R209H)	m	70 5.8	DY; CH; DC with infection/pain; no DS no therapy	None	4.75 moderate	Dysarthria, PEG	2 × 25/3 × 25	New case
6	c.626G>T (p.R209L)	m	58 4.8	MH; DY of legs; rare DC; no therapy	Generalised motor onset with tonic SZ at 3 y; SZ frequency 1/year; no therapy	7.75 moderate	Anarthria, neurological hip luxation	2 × 25	New case
7	c.674G>A (p.C225Y)	f	57 4.8	Severe MH; no DC; no therapy	Generalised motor onset; SZ free since Oxcarbazepine	6.5 moderate	Anarthria, swallowing impaired	2 × 25	4
8	c.625C>T (p.R209C)	f	266 22.2	DY; CH; MH; Tremor; Ataxia; DC and >10 × DS until DBS; CZP; Cannabis	None	6.25 moderate	Anarthria, DBS (15 y)	3 × 50	Not feasible, refused intake^4^
9	c.687C>G (p.S229R)	m	15 1.3	MH; generalised dystonia; Gabapentin; no DC	EIDEE, focal motor onset 5 d; EIMFS multiple all minor effects. VPA+LEV+STM frequency reduction	12.5 severe	Anarthria, PEG	2 × 25	New case
10	c.607G>A (p.G203R)	m	6 0.5	MH; action triggered DY; no therapy; no DC	EIDEE, focal motor onset 3 d phenobarbitone later gen. Motor onset. LEV, Lacosamide	7.75 moderate	–	2 × 25	New case
11	c.167T>G (p.I56S)	m	34 2.8	MH, generalised DY; no therapy; DC with infection	None	8.25 severe	Very irritable	2 × 25	New case
12	c.164T>C (p.I55T)	m	130 10.8	MH; mild DY; Gabapentin, no DC	Generalised motor onset 3 y; Sultiame seizure free currently no therapy	1.75 mild	Dysarthria psychiatric comorbidities	3 × 25	New case
13	c.625C>T (p.R209C)	f	125 10.4	Generalised DY; CH; Dopa; frequent DC; no DS	Generalised motor onset, 4 y; Oxcarbazepine; Clobazam seizure free	4.75 moderate	Anarthria	3 × 25	New case

The table summarises the demographic, genetic, and clinical baseline characteristics of all participants enrolled in the ZINCGNAO1 trial. Data are shown at study inclusion before initiation of zinc supplementation. Participants are listed by study identifier. Genetic variants are reported according to transcript NM_020988.3. Movement disorder phenotype, epilepsy status, previous and concomitant therapies, feeding modality, and trial participation status are indicated. Participants who discontinued the trial are explicitly identified.

Anarthria = inability to form words or phrases, with communication limited to ‘yes’ and/or ‘no’; CH = chorea; ClHy = chloral hydrate; CZP = clonazepam; DBS = deep brain stimulation; DC = dyskinetic crisis; Dopa = dopaminergic therapy; DS = dyskinetic status; DY = dystonia; Dysarthria = impaired articulation of speech with preserved ability to express needs or wishes; EIDEE = Early-infantile developmental and epileptic encephalopathy syndrome; EIMFS = epilepsy of infancy with migrating focal seizures; LEV = levetiracetam; m = months; MH = muscular hypotonia; PEG = percutaneous endoscopic gastrostomy; SZ = seizure; VPA = valproate or valproic acid.

Variant-specific zinc responsiveness was assessed post-inclusion using in-vitro functional assays of recombinant His_6_-tagged Gαo proteins, as previously described.^7^, ^8^, ^9^ Detailed methods for protein expression, purification, and guanosine triphosphate (GTP) binding and hydrolysis assays are provided in the Supplementary Methods.

### Randomisation and masking

No randomisation was performed because ZINCGNAO1 was a single-arm, open-label pilot trial. Participants, caregivers, treating clinicians, and study personnel were not masked to treatment allocation or study visit. No placebo or intervention concealment was used.

To reduce assessment bias for movement disorder severity, standardised videos were recorded at baseline, 3 months, and 6 months according to a predefined protocol. The videos were rated using the Burke-Fahn-Marsden Dystonia Rating Scale–Movement Score by an independent rater who was masked to participant identity, treatment timepoint, and chronological order of the three videos. The rater was not involved in trial conduct or clinical care of the participants.

### Outcomes

The primary outcomes were safety and feasibility. Feasibility was assessed via daily caregiver diaries and drug accountability, with treatment considered feasible if participants took the trial medication as recommended on at least 80% of the days of the trial. Safety was evaluated through adverse event monitoring and laboratory testing at each visit.

Secondary outcomes included motor function, assessed using the Gross Motor Function Measure-66 (GMFM-66) by physiotherapists, and dystonia severity, assessed by blinded video ratings using the Burke-Fahn-Marsden Dystonia Rating Scale—Movement Score (BFMDRS-M) by a blinded movement disorders expert. Additional secondary endpoints included dyskinesia (Abnormal Involuntary Movement Scale, AIMS), dystonia-related disability (BFMDRS—Disability Scale), quality of life (Caregiver Priorities and Child Health Index of Life with Disabilities, CPCHILD) and daily activities by occupational performance (Canadian Occupational Performance Measure, COPM). Caregiver diaries also captured dyskinetic crises, seizure frequency, sleep, general behaviour, and use of on-demand medication for acute worsening of the MD. The GNAO1-RD severity score was applied post-hoc to describe baseline disease severity and to support interpretation of clinical heterogeneity. This analysis was descriptive and was not prespecified as an efficacy analysis.

Exploratory post-hoc descriptive analyses were performed to examine clinical observations according to genotype, age at treatment initiation, and in-vitro zinc responsiveness category. These analyses were not prespecified and were not powered for formal subgroup inference. All secondary endpoints were analysed on an exploratory basis; no formal hierarchical testing strategy was prespecified for this pilot study.

### Statistical analysis

In this pilot study, a sample size of 12 participants was chosen as feasible for recruitment within the given timeframe, while sufficient to assess the feasibility and preliminary safety of oral zinc treatment. Statistically, this sample size (n = 12) minimises the imprecision of the estimated response probability, assuming a true response rate of 80% (calculated in R, binom.test package). The study was not powered to assess efficacy. Analyses were performed in predefined analysis sets, including a full analysis set, a modified full analysis set excluding participants who discontinued due to a non–treatment-related serious adverse event, and a safety analysis set including all participants exposed to at least one dose of zinc.

Baseline characteristics were summarised using descriptive statistics of valid data. Continuous variables were presented as mean (standard deviation) or median (interquartile range). Categorical variables are summarised as valid number (percentage). No formal methods for handling missing data were applied; analyses were based on available cases, and paired comparisons were restricted to complete observations for the relevant time points. The primary analyses of safety and feasibility were prespecified in the trial protocol. Analyses of secondary outcomes were also prespecified but exploratory, and no formal hierarchical testing strategy was prespecified. Post-hoc analyses were conducted to contextualise and further interpret selected findings. These included the comparison of GMFM-66 changes with published cerebral palsy reference trajectories, the propensity score–matched comparison with an external cerebral palsy cohort and the GNAO1-RD severity score analysis which was added during revision. These post-hoc analyses were intended to aid interpretation in the absence of an untreated GNAO1-RD control group and were not used to establish efficacy. Normality of continuous variables and outcome changes was assessed using the Shapiro–Wilk test with a significance level of 0.05, supported by visual inspection of Q–Q plots. Given the very small sample size, normality testing was considered exploratory and inherently inconclusive, and test selection was interpreted cautiously. Changes from baseline to follow-up visits were analysed using either paired t-test or Wilcoxon signed-rank test depending on whether the assumption of normality was met. Secondary endpoint analyses were exploratory. Effect sizes and corresponding 95% CIs were calculated for secondary outcomes to describe the magnitude and precision of observed effects regardless of statistical significance. Post-hoc non-parametric sensitivity analyses using Wilcoxon signed-rank tests were performed for the main secondary outcome comparisons, irrespective of distributional assumptions. Further details are given in the statistical analysis in the Supplementary Methods. Within-participant changes in GMFM-66 scores over 6 months were analysed as a prespecified exploratory secondary outcome. To contextualise these changes in the absence of an untreated GNAO1-RD control group, two post-hoc exploratory analyses were performed in a GMFM-66 comparative subset, defined as participants aged 2–18 years with complete baseline and 6-month GMFM-66 assessments. First, individual GMFM-66 changes were compared with published cerebral palsy reference trajectories. Second, GMFM-66 changes were compared with an external cohort of individuals with dyskinetic cerebral palsy using propensity score matching. Matching variables were selected a priori based on clinical relevance for gross motor development and availability in both datasets, including age, baseline GMFM-66, body weight, and height. Other potential confounders, including sex, baseline movement disorder severity, seizure burden, and concomitant treatments, were not included because of the small sample size, limited overlap with the external comparator dataset, and risk of overfitting. Details of the reference-trajectory comparison, propensity score matching, covariate balance assessment, and statistical testing are provided in the Supplementary Methods and Table S1. Residual confounding cannot be excluded, and these analyses were intended to support contextual interpretation rather than causal inference.

No formal sensitivity analyses were prespecified. All statistical tests were two-sided. For exploratory secondary outcomes, p values are nominal and were not adjusted for multiplicity.

Data preparation and statistical analyses were performed using R-version 4.4.0 (R Foundation for Statistical Computing, Vienna, Austria) and SPSS Statistics-version 30.0.0.0 (IBM Corp., Armonk, NY, USA).

**Fig. 1:**
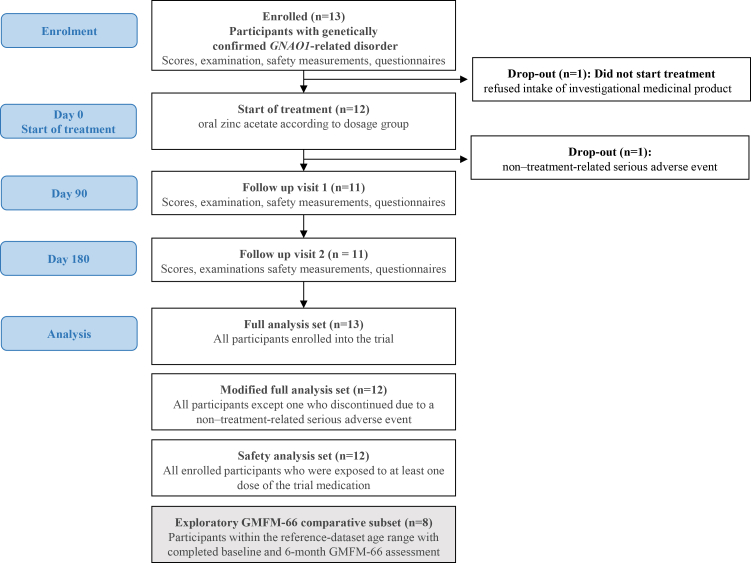
Flowchart of participant enrolment, treatment exposure, follow-up, and analysis populations in the ZINCGNAO1 trial. Sixteen individuals were screened, of whom 13 participants with genetically confirmed GNAO1-related disorder were enrolled and constituted the full analysis set. Twelve participants initiated oral zinc treatment; one participant discontinued before treatment initiation due to refusal of intake of the investigational medicinal product. One additional participant discontinued the trial due to a serious adverse event unrelated to the investigational medicinal product. The modified full analysis set comprised all participants except this individual. The safety analysis set included all participants exposed to at least one dose of zinc. For the exploratory post-hoc GMFM-66 comparison with reference trajectories, a restricted GMFM-66 comparative subset was defined, including only participants within the age range (2–18 years) covered by the reference dataset who completed both baseline and 6-month GMFM-66 assessments.

The different analysis sets are additionally illustrated in the participant flowchart (Fig. 1).

### Role of the funding source

The funder was involved in participant recruitment but had no role in study design, data collection, data analysis, data interpretation, or manuscript preparation.

## Results

Between Aug 2, 2024, and Jan 27, 2025, 16 individuals with genetically confirmed *GNAO1*-RD were screened, of whom 13 participants (P1–P13) were enrolled and constituted the full analysis set (Table 1; Fig. 1). There was one drop-out due to a non-treatment related serious adverse event. The modified full analysis set consists of 12 participants, and the safety analysis set of 12 participants as well. Seven participants were male and six were female and mean age at enrolment was 8.0 years (range, 0.5–25.1). Six participants had been included in a previously published cohort.^4^ All participants presented MD symptoms. Seven had a history of epilepsy of which five were treated with anti-seizure medication (ASM). Two participants had severe early-onset forms of epilepsy (onset in first year of life)^5^ whereas five participants presented with later-onset epilepsy. Two participants had seizures during the trial. P1 had one single seizure, whereas P9 diagnosed with Epilepsy of Infancy with Migrating Focal Seizures (EIMFS), a subtype of EIDEE, had multiple seizures during the trial. Dyskinetic crisis, defined as an acute exacerbation of the MD beyond the patient's usual baseline severity lasting for more than 1 h and accompanied by autonomic symptoms such as sweating, tachycardia, or insomnia, was specifically assessed in the patient diary. Dyskinetic crisis occurred in two participants (P5 once and P11 twice) during the trial. On demand medication for worsening of the MD was administered in seven participants on a mean of 11 days. At baseline, five (38%; 5/13) participants had a history of dyskinetic status, however no severe dyskinetic status occurred during the trial. Baseline epilepsy characteristics, antiseizure medication, and seizure course during zinc treatment can be found in Table S6.

Two participants (P1 and P9) had undergone bilateral deep brain stimulation of the globus pallidus internus (GPi-DBS) during pharmacorefractory dyskinetic status 2.0 and 7.2 years before enrolment. Of the trial cohort 8 participants (62%) were moderate in the GNAO1-RD severity score^10^ while 4 (31%) were severe and 1 was in the mild category. Detailed information of the participants including a baseline severity score for GNAO1-RD with sub scores can be found in Table S7.

Two participants discontinued the trial. One participant (P3) experienced a non–treatment-related serious adverse event (fracture of the dens axis with incomplete cervical myelopathy, and subsequent C0–C2 spondylodesis), and continuation of the trial was not in the best interest of the participant. Before this event occurred, treatment adherence was 80% in this participant. Another (P8), a 22-year-old female, was unable to ingest the capsules due to swallowing difficulties even after training with dummy capsules. The trial medication was suspended in various permitted vehicles and foods, but she refused all due to taste and therefore was never exposed and the treatment was assumed not feasible for her.

11 participants completed the 6-month trial. Overall feasibility was 84.6% in the full analysis set (11/13; 95% CI 54.6–98.1). In the modified full analysis set, feasibility was 83.3% (10/12; Clopper–Pearson 95% CI 51.6–97.9). Adherence was lowest during the initial 2 weeks, when nine participants reported gastrointestinal symptoms (nausea and/or vomiting), which resolved spontaneously. The bitter taste of Wilzin impaired intake, whereas participants who received the therapy via percutaneous endoscopic gastrostomy (PEG) achieved higher feasibility.

All adverse events are listed in Table 2. No treatment-related serious adverse events occurred during the trial. One participant experienced a treatment-related elevation of pancreatic enzymes (amylase and lipase) but remained asymptomatic. Two other participants showed clinically non-significant elevations of lipase and one of amylase. In all these participants the treatment was continued without adjustment, and the interval for follow-up blood tests was extended to three months. No copper or ferritin deficiency was observed in the safety analysis set throughout the trial. At baseline, nine participants had age-adjusted zinc deficiency. Zinc levels normalised in all treated participants after three months, with supraphysiological concentrations observed in eight individuals at 6 months. Laboratory results are summarised in Figure S2.

**Table 2:** Adverse events.

	Participants with intake of zinc (n = 12)
Participants without any adverse event	4 (33.3%)
with at least 1 adverse event	8 (66.6%)
with at least 1 serious adverse event∗	4 (33.3%)
Highest Severity∗	
Grade 4∗	1 (8.3%)
Grade 3	1 (8.3%)
Grade 2	2 (16.6%)
Grade 1	4 (33.3%)
Causal relation to zinc	
Participants with at least 1 related event∗∗	1 (8.3%)
with at least 1 not related event	7 (58.3%)

	**Adverse events (n = 11)**	**Outcome**

Serious adverse events		
Asystole∗	1 (9.0%)	Recovered/resolved
Bronchial infection	1 (9.0%)	Recovered/resolved
Commotio cerebri	1 (9.0%)	Recovered/resolved
Enterovirus infection with pneumonia∗	1 (9.0%)	Recovered/resolved with sequelae
Fracture of dens axis with dislocation∗	1 (9.0%)	Recovered/resolved with sequelae
Prolonged post-anaesthesia monitoring	1 (9.0%)	Recovered/resolved
Sum of serious adverse events	6 (54.5%)	
Non-serious adverse events		
Epistaxis	1 (9.0%)	Recovered/resolved
Mild fever after vaccination	1 (9.0%)	Recovered/resolved
Serum amylase increased∗∗	1 (9.0%)	Recovered/resolved with sequelae
Serum lipase increased∗∗	1 (9.0%)	Recovered/resolved with sequelae
Skin and subcutaneous tissue disorders–Fungal skin disorder nasolabial, scalp and penis.	1 (9.0%)	Recovering/resolving
Sum of non-serious adverse events	5 (45.5%)	

This table is an overview over the adverse events that occurred. Data are the frequency of participants (n, %) with at least one adverse event in the period from first intake of investigational medicinal product (IMP) to the 180 days visit in the safety population (n = 12, above) or the frequency of adverse events (n, % (n = 11, below)). For highest severity each patient is counted once with the event with highest grade. One patient (marked with ∗) had 3 serious adverse events which were not related to the intake of zinc. One was resolved (Asystole), the other two were resolved with sequelae. Another patient (marked with ∗∗) had two non-serious adverse events with relation to the intake of IMP, which resolved with sequelae.

**Fig. 2:**
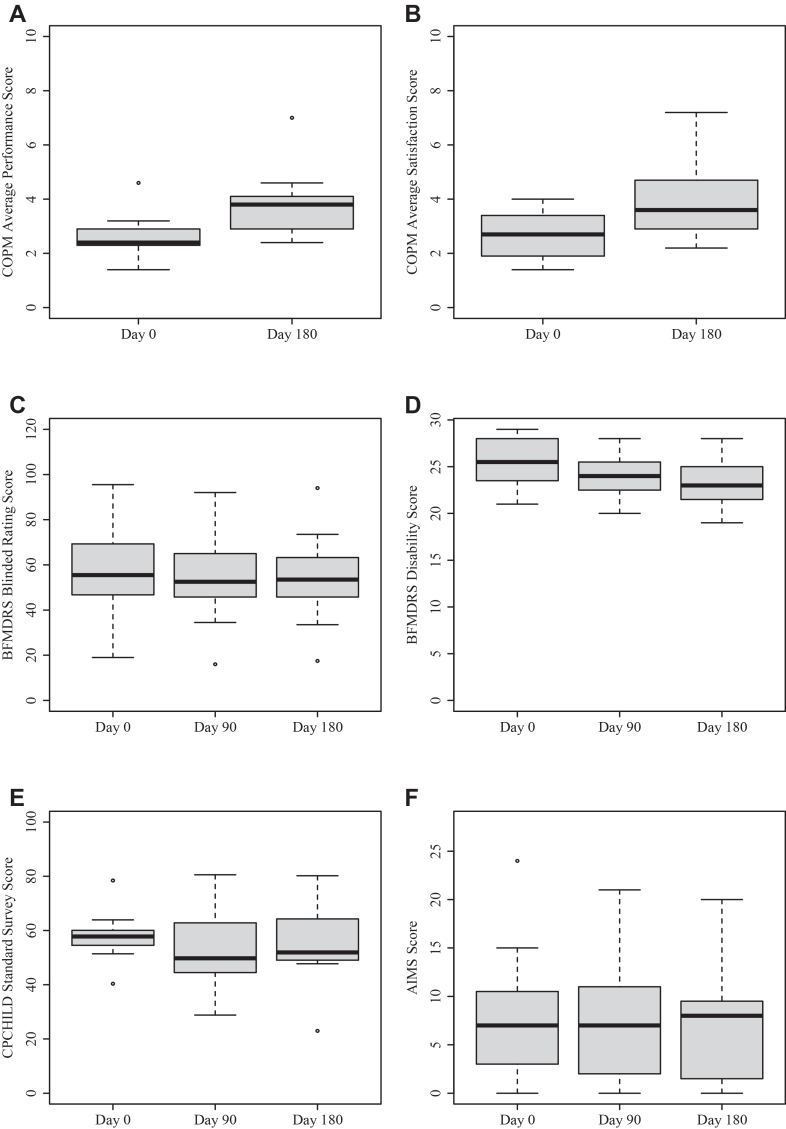
Changes in secondary clinical outcome measures assessed at baseline (day 0), day 90, and day 180 during zinc supplementation. (A) Canadian Occupational Performance Measure (COPM) performance score. (B) Canadian Occupational Performance Measure (COPM) satisfaction score. (C) Burke–Fahn–Marsden Dystonia Rating Scale (BFMDRS) movement score, assessed by a blinded rater. (D) Burke–Fahn–Marsden Dystonia Rating Scale (BFMDRS) disability score, assessed by an unblinded rater. (E) Caregiver Priorities and Child Health Index of Life with Disabilities (CPCHILD). (F) Abnormal Involuntary Movement Scale (AIMS). Box plots show the median, interquartile range, and range; individual outliers are indicated. Data are shown for the modified full analysis set.

**Fig. 3:**
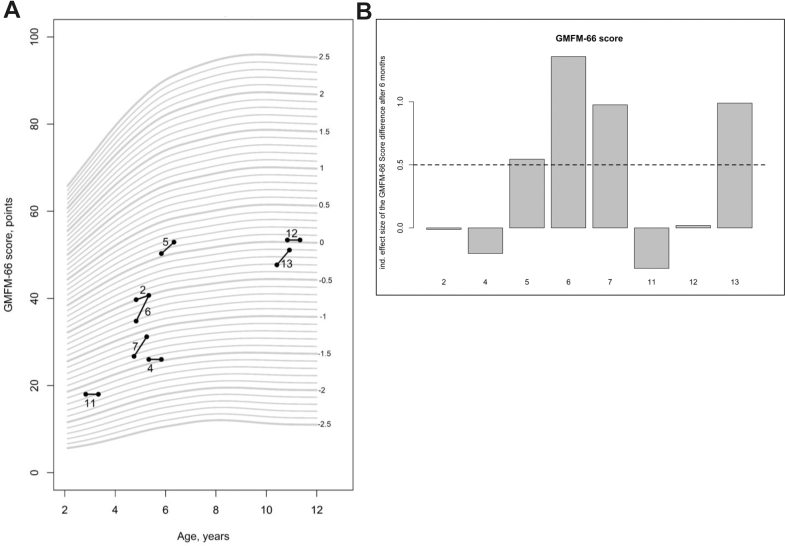
Gross Motor Function Measure–66 (GMFM-66) trajectories and effect sizes in the exploratory GMFM-66 comparative subset. (A) Individual GMFM-66 trajectories from baseline to 6 months in participants included in the exploratory GMFM-66 comparative subset (n = 8), defined by the age range covered by the reference dataset and availability of baseline and 6-month GMFM-66 assessments. Trajectories are shown in relation to published reference trajectories for individuals with dyskinetic cerebral palsy receiving standard care. (B) Effect size, expressed as z score, of the change in GMFM-66 score from baseline to 6 months in the same subset. Values above 0.5 indicate improvement exceeding that expected from reference trajectories.

Secondary outcomes were assessed in the modified full analysis set (n = 12) (Figs. 2 and 3). The mean baseline COPM Performance score was 2.6 (SD 0.8) and increased to 3.8 (SD 1.3) at 6 months (nominal p = 0.003). The mean baseline COPM Satisfaction score was 2.7 (SD 0.9) and increased to 4.1 (SD 1.7) (nominal p = 0.002). Three participants (P4, P10 and P13) in this cohort had a clinically important change of ≥2 points (COPM Manual 2019). Mean GMFM-66 scores increased over the study period, from baseline (mean 33.1 [SD 12.8]) to three months (mean 35.0 [SD 13.8]) and 6 months (mean 37.8 [SD 12.8]). In the prespecified exploratory within-cohort analysis GMFM-scores increased from baseline to six-month timepoint (nominal p = 0.006). For the exploratory post-hoc comparison with reference trajectories, a restricted GMFM-66 comparative subset was defined, including participants within the age range covered by the reference dataset who completed both baseline and six-month GMFM-66 assessments (n = 8). Fig. 3A shows individual GMFM-66 trajectories; four participants had greater improvement than expected from the natural course of cerebral palsy, with an effect size corresponding to a z-score greater than 0.5 (Fig. 3B).

In an additional exploratory propensity score–matched comparison with individuals with cerebral palsy, participants receiving zinc had a larger increase in GMFM-66 scores than matched controls (mean change 2.21 [SD 2.17] vs 0.43 [SD 2.06]; nominal p = 0.040), corresponding to a large effect size (Cohen's d 0.88 [95% CI 0.05–1.71]). Covariate balance after propensity-score matching was adequate overall; further details, including standardised mean differences, are provided in the Supplementary Methods and Table S1.

The mean BFMDRS-M evaluated by a blinded rater did not show clear change over time (baseline: 57.8 [SD 20.8]; three months: 54.3 [SD 20.1]; 6 months: 54.3 [SD 20.3]). The mean BFMDRS Disability Score decreased from baseline to 6 months (baseline: 25.5 [SD 2.6]; three months: 24.2 [SD 2.6]; 6 months: 23.5 [SD 2.8]; nominal p = 0.005). The mean AIMS score and mean CPCHILD score did not show clear change over time. Patient individualised effect trajectories of secondary outcomes and BFMDRS Disability sub scores are listed in Table S3 and individual patient trajectories of secondary clinical outcome measures in Figure S1.

Post-hoc non-parametric sensitivity analyses yielded results consistent with the corresponding parametric analyses and did not change the interpretation of the secondary outcome findings (Table S2).

Regarding genotype–phenotype correlation of the individual patients, zinc was well tolerated in P10, a participant aged 6 months at treatment initiation, representing the youngest individual documented to receive this therapy. This participant carried the G203R mutation, associated with a severe phenotype, yet in this trial he showed a comparatively favourable clinical course without seizures or sleep disturbance under zinc supplementation.

All the participants (P1, P5, P6 and P13) carrying variants affecting arginine residue 209 showed improvements in GMFM-66 scores during the trial. P1, who was the oldest participant, had pre-existing fixed orthopaedic deformities, which may have constrained motor gains. In this small treatment group, variants involving arginine 209 and glycine 203 and younger age at treatment initiation appeared to coincide with greater motor improvement.

One participant with *in vitro* zinc responsiveness (P11) did not show obvious clinical improvement and discontinued zinc after trial completion. All other participants who initiated treatment continued zinc supplementation beyond the study period, reflecting sustained caregiver-perceived benefit. In ten participants, caregivers reported improvements in cognition, while quality of sleep did not change according to the diary.

All genetic variants of the participants in this trial (Table 1) showed *in vitro* responsiveness to zinc in Group II (increased GTP hydrolysis; no effect on guanosine-5′-O-(3-thiotriphosphate) (GTPγS) binding) or III (effect on both GTP hydrolysis and GTPγS binding) as detailed in Table S5 and Figure S3.

## Discussion

This study presents the first clinical trial of oral zinc supplementation in individuals with *GNAO1*-RD. The pilot trial provides preliminary evidence that oral zinc supplementation is safe and feasible over a six-month treatment period.

Zinc was generally well tolerated, with no treatment-related serious adverse events observed. Mild gastrointestinal adverse effects were common but transient and predominantly occurred during the first two weeks of treatment, consistent with the known side-effect profile of oral zinc salts.

Treatment feasibility exceeded the predefined threshold, with adherence of at least 80% in most participants. Palatability (bitter taste) and swallowing difficulties, rather than tolerability, emerged as the main limitations, particularly in participants without a PEG, highlighting the importance of formulation choice in patients with severe neurodisability in general and specifically in *GNAO1*-RD.

No clinically relevant safety concerns were identified during the six-month treatment period. Mild elevations in pancreatic enzymes were observed, a known and typically clinically insignificant effect of long-term zinc administration.^11^ Baseline zinc deficiency was detected in several participants, a finding not previously reported in *GNAO1*-RD. Although zinc deficiency is not considered common in paediatric populations, strict pre-analytical handling was ensured in this study. In routine clinical practice, longer pre-analytical times may artificially elevate zinc concentrations, suggesting that zinc deficiency may be underdiagnosed.^12^ Although no abnormalities in copper or iron metabolism were observed, longer-term zinc administration may be associated with secondary micronutrient deficiencies, due to reduced gastrointestinal absorption,^13^ supporting the need for ongoing laboratory monitoring during prolonged treatment.

Zinc was administered to achieve supraphysiological serum concentrations, aiming to facilitate central nervous system penetration through steeper concentration gradient.^14^ However, zinc competes with magnesium for binding to Gαo, suggesting that alternative formulations or delivery strategies—including intravenous, intrathecal, prodrug-based,^15^ or nanoparticle-mediated intranasal approaches^16^ —may warrant future investigation to optimise central nervous system bioavailability. Given that symptom onset in *GNAO1*-RD typically occurs in early infancy, initiation of treatment as early as possible may be relevant and should be explored in future studies.

Exploratory analyses showed improvements in gross motor function, occupational performance, and disability scores, with reduced disability appearing to be driven mainly by improvements in daily activities such as eating and drinking. The COPM captured individualised, participant-relevant functional changes, offering sensitivity not achieved by impairment-based scales alone. GMFM-66 increased over 6 months. However, raw score increases are difficult to interpret in a paediatric population with ongoing development and age dependency variations. Therefore, an exploratory comparison with published GMFM-66 reference trajectories in children with cerebral palsy (CP) was performed and reflected that a subset of participants improved beyond what would be expected from those reference trajectories. Notably, four of eight participants exceeded the threshold corresponding to a moderate effect size (z score >0.5), indicating possible clinical relevance. The findings were further corroborated by an exploratory propensity score–based comparative analysis, where zinc-treated participants demonstrated larger gains in GMFM-66 scores and a large effect size. However, these findings should be interpreted with caution. The consistency of change across multiple analytical approaches may support an association between zinc supplementation and observed improvements, although causal inference is limited by the open-label design and the use of non–disease-specific comparator data. Because the GMFM-66 was developed for cerebral palsy, it may not fully capture clinically meaningful functional changes in GNAO1-RD, a hyperkinetic disorder characterised by episodic dyskinetic crises. Although improvements in GMFM-66 scores in milder GNAO1-RD could partly reflect natural maturation, only one participant in our trial was classified as having mild disease according to the GNAO1-RD severity score. Most participants had moderate or severe disease, in whom ongoing motor development is not commonly reported. Consistent with this interpretation, a recent longitudinal study of GNAO1-RD,^17^ published after completion of our trial and therefore not available when our study was designed, found no statistically significant longitudinal change in gross motor function measured with GMFM-88, nor in the motor skills domain of the Vineland Adaptive Behaviour Scales. Because that study used GMFM-88 rather than GMFM-66, direct comparison with the present GMFM-66 findings should be made cautiously.

In contrast, dystonia severity and hyperkinetic movements, remained largely unchanged, consistent with the often refractory nature of dystonia in *GNAO1*-RD. However, the observed decrease in dystonia related disability suggests that zinc supplementation may have reduced the functional impact of dystonia in some patients, even in the absence of measurable changes in dystonic movements themselves. This dissociation between dystonia severity and functional disability is also consistent with improvements observed in COPM performance and satisfaction scores. These patient-centred outcomes capture individualised, goal-oriented changes that may be particularly relevant in disorders characterised by complex, heterogeneous motor phenotypes. At the same time, the open-label design does not exclude expectation-driven bias, particularly for caregiver-reported outcomes.

Quality of life did not change over the relatively short observation period. Given the broad scope of this instrument and the high baseline burden of disease, longer follow-up or larger sample sizes may be required to detect changes at this level.

Caregivers reported fewer and shorter infections, consistent with known immunomodulatory effects of zinc,^18^^,^^19^ whereas seizure frequency remained unchanged. These observations may have been influenced by the small number of participants with active seizures and the short observation period, especially since favourable effects of zinc on epilepsy have been reported in other settings.^20^ Sleep quality did not change, although diary-based assessment was unreliable and should be replaced by validated sleep instruments in future studies.

Overall, these exploratory results should be interpreted cautiously and used to influence the design of future controlled trials in *GNAO1*-RD.

In clinical practice, at present zinc supplementation should be regarded as an experimental add-on therapy in patients with GNAO1-RD whose variants show in-vitro responsiveness to zinc. This approach should be considered experimental and should require shared decision-making and close monitoring of the treating physician and would not preclude escalation of established therapies for MDs or epilepsy, including deep brain stimulation in patients with severe or refractory dyskinetic crises. Further long-term data are needed to determine the optimal timing of initiation, durability of clinical benefit, and the extent to which increased serum zinc concentrations translate into meaningful CNS exposure. Our findings should be viewed within the broader shift towards precision medicine in rare genetic MDs,^21^ in which molecular diagnosis, variant-level functional characterisation, and targeted or mechanism-based therapies are increasingly used to guide treatment development; in GNAO1-RD, future studies will need to establish whether in-vitro zinc responsiveness predicts clinical benefit.

This study has several limitations. Although blinded assessment was implemented for dystonia ratings, the open-label design without an internal control group remains the main study limitation, introducing potential bias, including placebo effects, regression to the mean, developmental confounding, and expectation-driven reporting particularly for caregiver-reported outcomes. Selection bias and clinical heterogeneity should also be considered, as recruitment through a specialised tertiary referral centre and patient advocacy networks might have enriched the cohort for highly motivated families, while diverse clinical presentations might have influenced outcome assessment. Caregiver-reported outcomes might have been further affected by expectation-driven reporting, particularly because this was the first interventional trial in GNAO1-RD. Awareness of active treatment might also have increased caregiver engagement, stimulation, and encouragement during the study. The small sample size limits statistical power and precludes definitive conclusions regarding efficacy, while also restricting exploration of treatment effects across clinically relevant subgroups defined by age, genotype or disability severity.

Wide confidence intervals were observed for the estimated differences in secondary endpoints over time, which is expected given the limited sample size inherent to a pilot study design, thereby limiting the precision of the findings. Because of the small sample size and clinical heterogeneity of this ultra-rare disorder, analyses stratified by sex were not performed, and the potential influence of sex or gender on treatment feasibility, safety, or exploratory outcomes could not be assessed. The small sample size also limits the robustness of statistical inference, including assessment of distributional assumptions and test selection for paired comparisons; therefore, all secondary endpoint analyses should be interpreted as exploratory and descriptive.

At time of study design, disease-specific natural history data were not available, necessitating comparison with dyskinetic CP reference populations, to contextualise motor outcomes. While CP is biologically non-equivalent to *GNAO1*-RD, it provided a pragmatic external context for interpreting the results in an ultra-rare disorder without an untreated comparison group. The application of both trajectory-based reference data and a matched external comparator propensity score matching, represents a pragmatic exploratory strategy in the context of an ultra-rare disorder for which untreated natural history data are unavailable and randomised control groups are not feasible. Although age, baseline GMFM-66, body weight, and height were selected as clinically relevant and commonly available matching variables, residual confounding cannot be excluded. Other potentially relevant variables, including sex, movement disorder severity, seizure burden, and concomitant treatments, could not be incorporated into the propensity score model because of the small sample size, limited overlap with the external comparator dataset, and the risk of overfitting.

The restricted age range of validated GMFM-66 reference data limited eligibility for some analyses, underscoring the lack of validated outcome measures for adolescents and adults with severe childhood-onset neurodevelopmental disorders. The treatment duration may have been insufficient to capture longer-term functional or quality-of-life effects, and the long-term safety of sustained supraphysiological zinc exposure remains uncertain. Finally, formulation-related tolerability affected feasibility in two participants, and although all variants demonstrated *in vitro* zinc responsiveness, the relationship between molecular effect size and clinical benefit could not be examined and warrants further investigation. Accordingly, the present findings should be considered hypothesis-generating and cannot establish efficacy.

Key strengths include the prospective, interventional design in an ultra-rare disorder with no established disease-modifying treatments. The trial demonstrates feasibility of conducting systematic clinical research in *GNAO1*-RD despite its low prevalence and marked phenotypic heterogeneity. Importantly, zinc supplementation was applied within a mechanism-based, precision-medicine framework, supported by variant-specific *in vitro* functional data. All *GNAO1* variants carried by participants showed zinc responsiveness, providing biological plausibility for the intervention and strengthening the translational link between molecular dysfunction and clinical observations.

The study further benefits from a comprehensive, multidimensional outcome assessment. Patient-centred functional measures enabled detection of individualised changes beyond standardised motor scales. Together with systematic laboratory safety monitoring and high treatment adherence despite the severe disability, these features enhance the interpretability and clinical relevance of the findings within the constraints of ultra-rare disease research.

Another strength of this study is the integration of clinical outcomes with variant-specific functional data. All *GNAO1* variants carried by participants demonstrated zinc responsiveness *in vitro*, consistent with Group II or III effects on GTP binding and/or hydrolysis. This concordance supports the biological plausibility of zinc as a targeted modulator of Gαo dysfunction and provides a mechanistic framework linking molecular effects to clinical observations. These findings also suggest that in-vitro functional profiling may be useful for future trial design and, potentially, for individualised treatment decisions in *GNAO1*-RD.

This pilot study provides preliminary evidence that oral zinc supplementation is safe and feasible in *GNAO1*-RD. Exploratory analyses suggested possible improvements in motor function and daily activity and dystonia-related disability, but these findings remain hypothesis-generating. Larger multicentre randomised, crossover, delayed-start, or N-of-1 trials, together with optimised paediatric formulations and longer follow-up, are needed to assess efficacy and define long-term benefits.

## Contributors

MT conceived and designed the trial, was responsible for data acquisition, and drafted the manuscript. KM and HD contributed to trial conduct and participant recruitment. PS and JW developed the statistical analysis plan and performed the statistical analyses. MT, BH, PS, and JW accessed and verified the underlying data. ID conducted additional analyses related to the Gross Motor Function Measure–66. BH contributed to the study protocol, trial planning, and coordinated data acquisition at the clinical trial centre. YL and AKo performed the basic research experiments, including assessment of zinc responsiveness of the *GNAO1* variants. VK contributed to trial planning and coordinated the basic research component. AKy and VK provided substantial input into trial design from both basic science and clinical perspectives. All authors critically revised the manuscript for important intellectual content and approved the final version for publication. All authors meet the ICMJE criteria for authorship.

## Data sharing statement

The study protocol, statistical analysis plan, and supporting materials will be available immediately after publication of this Article. Deidentified individual participant data that underlie the results reported in this Article, together with the corresponding data dictionary where applicable, will be available beginning 2 years and ending 5 years after Article publication. Data will not be made publicly available because the study involves a small cohort of children, adolescents, and young adults with an ultra-rare disorder, for whom re-identification might be possible despite deidentification. Data will be shared with investigators whose proposed use of the data has been approved by an independent review committee identified for this purpose, for analyses aimed at achieving the objectives of the approved proposal. Proposals should be directed to kinderklinik-studiensekretariat@uk-koeln.de. Data requestors will be required to sign a data access agreement and comply with applicable data protection requirements before access is granted. No custom software was developed for this study; statistical analyses were performed using the software specified in the Methods section. Analysis scripts and relevant code used to generate the reported statistical analyses will be made available with approved data access requests, where applicable. Public release of individual-level test data is not planned because the study involves a small cohort of children, adolescents, and young adults with an ultra-rare disorder, for whom re-identification might be possible despite deidentification.

## Declaration of interests

The authors declare that they have no competing interests.

## Appendix A Supplementary data

Supplementary Appendix

Translated Abstract

Protocol
