# Supplementary material for: Safety and feasibility of oral zinc for patients with GNAO1-related disorders (ZINCGNAO1): an open-label, single-arm, single-centre, pilot trial in Germany
Source: eClinicalMedicine. 2026 Jul 23;98:104092. doi: 10.1016/j.eclinm.2026.104092 (PMC13427569; doi:10.1016/j.eclinm.2026.104092)
Supplement: Supplementary Appendix [file mmc1.pdf]

Supplementary Appendix

Supplement to:

Safety and feasibility of oral zinc for patients with *GNAO1*-related disorders (ZINCGNAO1): an open-label, single-arm, single-centre, pilot trial in Germany

This appendix has been provided by the authors to give readers additional information about the work.

TABLE OF CONTENTS

1 Sponsor, Data Monitoring Committee (DMC), and cooperation partner 2

2 Supplementary Methods 3

Inclusion and exclusion criteria 3

***In vitro* assessment of *GNAO1* variants** 4

Further statistical methods: Details of the additional evaluations of the motor function using the GMFM-66 5

3 Tables 6

Table S1. Propensity Score-matched analysis of changes in GMFM-66 scores 6

Table S2. Longitudinal changes in secondary clinical outcome measures 7

Table S3. BFMDRS-Disability scale of individual items 8

Table S4. Group-level summary of baseline characteristics 9

Table S5. Three categories of pathogenic Gαo based on their responses to zinc *in vitro* 10

Table S6. Baseline epilepsy characteristics, antiseizure medication, and seizure course during zinc treatment 11

Table S7. Baseline GNAO1-related disorder severity scores in trial participants 12

Table S8. Longitudinal changes in secondary clinical outcome measures grouped by baseline severity score 13

4 Figures 14

Figure S1. Individual patient trajectories of secondary clinical outcome measures 14

Figure S2. Number of patients shifting between laboratory value categories at each visit. 15

Figure S3. Effects of zinc on novel pathogenic Gαo variants of participants 16

5 References 17

6 Statistical Analysis Plan 18

1 Sponsor, Data Monitoring Committee (DMC), and cooperation partner

Sponsor

University of Cologne

Represented by:

Dr. Moritz Thiel

Children's Hospital University Hospital Cologne

Kerpener Strasse 62

50937 Cologne, Germany

Data Monitoring Committee

Member of DMC	Affiliation
Joachim Opp	Sozialpädiatrisches Zentrum, Evangelisches Krankenhaus Oberhausen, Oberhausen, Germany
Burkhard Stüve	Pediatric Neurology, DRK-Kinderklinik Siegen gGmbH, Siegen, Germany
Walid Fazeli	Pediatric Neurology, University of Bonn, Faculty of Medicine, Bonn, Germany.
Corine Baljé-Volkers	Clin-Q B.V.; 9723 DJ Groningen; Netherlands

Clinical project Management

Clinical Trials Unit, Paediatrics

University of Cologne

Kerpener Str. 62

50937 Cologne, Germany

Project Management, Data Management, Monitoring

CTCC (Clinical Trials Center Cologne)

University of Cologne

Gleueler Str. 269

50935 Cologne, Germany

Study Statistician

University of Cologne

Institute of Medical Statistics and Computational Biology (IMSB)

Kerpener Str. 62

50937 Cologne, Germany

2 Supplementary Methods

Inclusion and exclusion criteria

Inclusion criteria

- *GNAO1* associated neurological disorder, documented by either:
  Proven pathogenic or likely pathogenic mutation in *GNAO1* or a variant of unknown significance in *GNAO1* and clinical symptoms likely to be consistent with *GNAO1* as determined by the investigators
  and
  at least one of the common symptoms of *GNAO1*: Movement disorder (dystonia, chorea, ataxia, stereotypic movements, clonic), central muscular hypotonia, epilepsy, global developmental delay
- Age: 6 months to 30 years
- GMFM ≤ 75*
- Written informed consent prior to any trial-related procedure by parents or legal guardian
  (due to severe cognitive impairment, informed consent by participants is not feasible even with older age or in adulthood)
- Stable on the following concomitant treatments for at least 3 months before trial inclusion: anti-seizure medication, baclofen, and deep brain stimulation setting

Exclusion criteria

- Treatment of Zinc in the last 4 months before inclusion
- Known other genetic variants that are known to cause symptoms like observed in *GNAO1*-related disorders, additional to the proven *GNAO1* mutation
- Implantation of Deep brain stimulation planned during the duration of the trial, i.e. in the six months after inclusion
- Start of intrathecal baclofen therapy planned during the duration of the trial, i.e. in the six months after inclusion
- Known allergy/hypersensitivity to the scheduled trial drug
- Concomitant participation in other clinical drugs with investigational drugs or with competing interventions
- Sexually active participants who are not willing to use/ not using a highly effective contraception method with a pearl-index < 1. Sexually active participants resp. their partner, unless surgically sterile, must be using a highly effective contraception method (including oral, transdermal, injectable or implanted contraceptives, IUD, using a condom of the sexual partner or sterile sexual partner) and must agree to continue using such precautions during the whole study period.
- Pregnant women and nursing mothers

* The GMFM-66 eligibility threshold of 75 or lower was chosen to ensure that participants had measurable motor impairment and to avoid inclusion of asymptomatic or minimally affected individuals; it was not intended to define a homogeneous efficacy population. Given the broad clinical spectrum of GNAO1-RD, inclusion across severity levels supported assessment of safety and feasibility in a representative cohort, but clinical heterogeneity and severity-related floor effects might have limited the ability to detect changes in exploratory motor outcomes

In vitro assessment of *GNAO1* variants

Post-inclusion, variant-specific Zn^{2+} sensitivity was evaluated *in vitro* using recombinant His_6-Gαo produced in *E.coli* as previously described. Briefly, pET23b plasmids encoding different variants of His_6-Gαo were transformed into *E.coli* Rosetta(DE3)pLysS (Novagen, 70956). Transformed bacteria were grown at 37°C to an OD_{600}=0.6 before induction with 0.25mM isopropyl-β-D-thiogalactopyranoside (IPTG, Biosynth, EI05931) at 18°C overnight. Bacteria were centrifuged at 3,500xg at 4°C and resuspended in TBS (20mM Tris-HCl (pH 7.5) and 150mM NaCl) supplemented with 1mM PMSF and 30mM imidazole (all from Sigma-Aldrich). Cells were lysed using One Shot Cell disrupter (Constant Systems) and centrifuged at 15,000xg for 15min at 4°C. Cleared lysate was incubated with Ni-NTA agarose beads (QIAGEN) in a rotary shaker at 4°C overnight. The beads were washed repeatedly with 10 resin volumes of ice-cold wash buffer (TBS supplemented with 10mM imidazole) and bound proteins were GDP-loaded in TBS supplemented with 3% glycerol, 10 mM $MgCl_2$, 0.1 mM DTT, and 200 µM GDP (all from Sigma-Aldrich). Recombinant Gαo was eluted with TBS containing 300 mM imidazole. Protein concentration was measured using the Bradford assay, and the purity was analysed by SDS-PAGE followed by Coomassie staining.

The sensitivity of recombinant pathogenic Gαo towards Zn^{2+} was evaluated in GTP binding/hydrolysis assays using BODIPY-GTPγS or BODIPY-GTP (both from Jena Bioscience)[1,2]. Recombinant Gαo was diluted to 1 µM in the reaction buffer [TBS supplemented with 10 mM $MgCl_2$ and 0.5% bovine serum albumin (BSA)] and mixed with 25-100 µM of $ZnCl_2$ (Sigma-Aldrich). The mixture was then pipetted into black 384-well plates (Greiner), and BODIPY-GTP or BODIPY-GTPγS was added to the wells to a final concentration of 1µM. Fluorescence measurements were performed at 28°C in a Tecan Infinite M200 PRO plate reader with excitation at 485nm and emission at 530nm. Data from the assay was plotted into graphs using GraphPad Prism v.10.5.0.

Further statistical methods: Details of the additional evaluations of the motor function using the GMFM-66

The GMFM-66 is a validated instrument for assessing motor abilities in children with cerebral palsy (CP) [3]. Given that children with *GNAO1*-RD phenotypically resemble those with dyskinetic CP ("CP mimics") [4], the GMFM-66 was applied in this cohort. The GMFM-66 is a standardized, criterion-referenced tool derived from the original GMFM-88 using Rasch analysis to improve scaling, interpretability, and precision across the spectrum of motor abilities [3]. The measure provides a single interval-level score (range 0–100) representing overall gross motor function, allowing for longitudinal and comparative analyses in both clinical and research settings.

In children with CP, the GMFM-66 typically increases with age, particularly in those younger than 10 years[5], therefore the information of an observed increase is limited. Therefore, in addition to the the planned raw GMFM-66 analysis, two further statistical approaches were used to assess change in GMFM-66 scores:

Comparison with reference percentiles for GMFM-66

Since no control group was available in the present study, and no historical data exist describing 6-month GMFM-66 trajectories in children with *GNAO1*-RD, GMFM-66 score changes were compared with a historical cohort of children with CP using the reference percentiles for the GMFM-66 in children with CP published by Duran and colleagues, receiving standard care, together with a method for quantifying individual change over 6 months using an effect-size approach comparable to Cohen's d for dependent groups [5]. The mathematical formulation of the method is described in detail in the original publication[5].

A limitation of this reference dataset is its restricted age range (2–18 years) allowing only the inclusion of a subset of all participants of the appropriate age (n=8) in this analysis.

Propensity score-based comparison

A propensity score (PS)-based analysis was conducted to further evaluate GMFM-66 changes while accounting for potential confounding. PS methods are commonly used in observational research to improve baseline comparability between exposure groups.[6,7] Because no data were available for *GNAO1*-RD without zinc supplementation, a comparison cohort was drawn from the follow-up phase of the "On your feet" neuromuscular rehabilitation program[8]. This program combined intensive, goal-directed training with home-based whole-body vibration therapy over 6 months, with assessments at baseline (M0), 6 months (M6), and 12 months (M12; follow-up). All participants received standard CP care throughout the study period.

For the present analysis, data from M6 and M12 were used to calculate 6-month GMFM-66 changes. Eligible participants (2006–2025) had a diagnosis of dyskinetic CP (ICD-10: G80.3) or spastic–dyskinetic CP (G80.8) and complete GMFM-66, age, weight, and height data at both time points. Data from 137 participants aged 3–22 years were available. Ethical approval for use of these data was granted by the Ethics Committee of the University of Cologne (reference 16–269), and the registry is publicly accessible (DRKS0001131).

Given the limited number of treated participants (n=10) and the large control pool (n=137), we performed 1:2 matching for the following parameters: GMFM-66 baseline score, age, weight and height with the nearest neighbour ("nearest") method and a ratio of 2, without replacement to increase statistical power while maintaining covariate balance, as recommended by Austin [9] and Rassen et al. [10]. According to Rosenbaum and Rubin, covariates can be considered sufficiently balanced if the absolute standardized mean difference (SMD) is less than 0·25 [11]. However, because lower thresholds have also been proposed, including an SMD of 0·1, covariate balance was additionally reviewed using this stricter criterion. Only body weight showed minor residual imbalance (SMD 0·121), whereas age and baseline GMFM-66 score, the covariates considered most prognostically relevant for GMFM-66 change, were balanced with SMDs below 0·1. Given the small number of treated participants and the limited prognostic relevance of this minor imbalance, no additional covariate-adjusted sensitivity analysis was performed.

The additional analyses focusing on the GMFM-66 were performed in R (version 4·5·1) using RStudio (version 2025·09·0, Build 38). Propensity score matching was conducted with the MatchIt package (version 4·7·2)[12], and effect sizes were calculated using the effsize package (version 0·8·1)[13].

3 Tables

Table S1. Propensity Score-matched analysis of changes in GMFM-66 scores

	**All**			**PS-matched**		
	**Study cohort (n=10)**	**Control group (n=137)**	**SMD**	**Study cohort (n=10)**	**PS-matched control group (n=20)**	**SMD**
Mean age, years	7·53	7·42	0·015	7·53	6·9	0·091
Mean height, cm	107·2	116·3	-0·343	107·2	106·0	0·043
Mean weight, kg	18·2	21·3	-0·252	18·2	16·7	0·121
Mean initial GMFM-66 score	35·5	42·9	-0·595	35·5	36·0	-0·040
Mean propensity score	0·184	0·060	0·692	0·184	0·145	0·219
	**Study cohort (n=10)**	**PS-matched control group (n=20)**	**p-value**	**Effect size (Cohen's d)**		
Change in GMFM-66 between baseline and 6 month	2·21 (2·17)	0·43 (2·06)	0·040	0·88 (0·05; 1·71)		

Study cohort for the propensity score-based analysis and analysis results. PS propensity score, SMD Standardized mean difference. The distance metric for propensity score was estimated using a generalized linear model ("glm" distance in matchit R-package). The ratio study participant to control cohort was set to 2. For more details see above in supplemental methods.

Table S2. Longitudinal changes in secondary clinical outcome measures

	Baseline	Visit 2	Visit 3	Change to visit 2		Wilcoxon sensitivity p value, baseline to visit 2	Change to visit 3		Wilcoxon sensitivity p value, baseline to visit3
	(at day 0)	(at day 90)	(at day 180)	(after 90 days)	effect size (95% CI)		(after 180 days)	effect size (95% CI)	
**GMFM-66**	33·1 (12·8), n=12	35·0 (13·8), n=11	37·8 (12·8), n=10	1·3 (-0·7 to 3·4), p=0·180	0·436 (-0·194 to 1·047)	0·123	2·3 (0·8 to 3·8), p=0·006	1·123 (0·302 to 1·908)	0·018
**CPCHILD**	57·9 (8·8), n=12	53·8 (14·7), n=11	55·5 (15·0), n=11	-4·2 (-12·0 to 3·6), p=0·260	-0·362 (-0·964 to 0·258)	0·424	-2·6 (-10·6 to 5·5), p=0·497	-0·212 (-0·805 to 0·391)	0·722
**COPM-Performance**	2·6 (0·8), n=12	··	3·8 (1·3), n=11	··	··	··	1·3 (0·6 to 2·1), p=0·003	1·196 (0·395 to 1·965)	0·005
**COPM - Satisfaction**	2·7 (0·9), n=12	··	4·1 (1·7), n=11	··	··	··	1·5 (0·7 to 2·3), p=0·002	1·230 (0·418 to 2·008)	0·005
**BFMDRS-Disability**	25·5 (2·5), n=12	24·2 (2·6), n=11	23·5 (2·8), n=11	-1·0 (-1·9 to 0·0), p=0·049	-0·674 (-1·320 to -0·002)	0·040	-1·6 (-2·6 to -0·6), p=0·005	-1·090 (-1·829 to -0·318)	0·017
**BFMDRS-Movement**	57·8 (20·6), n=12	54·3 (20·1), n=11	54·3 (20·3), n=11	-1·0 (-2·6 to 0·7), p= 0·260	-0·399 (-1·006 to 0·226)	0·249	-1·0 (-2·3 to 0·4), p=0·091*	-0·491 (-1·109 to 0·147)	0·091
**AIMS**	7·8 (6·9), n=12	7·1 (6·5), n=11	6·8 (6·1), n=11	-0·8 (-1·9 to 0·2), p=0·110	-0·532 (-1·155 to 0·113)	0·111	-1·1 (-2·6 to 0·4), p=0·140	-0·483 (-1·100 to 0·154)	0·120

Secondary clinical outcome measures assessed at baseline (day 0), visit 2 (day 90), and visit 3 (day 180), and corresponding changes from baseline over the 6-month treatment period. Data are presented for the modified full analysis set (n=12). Continuous variables are shown as mean (SD) with sample size (n) or as mean change from baseline with 95% confidence intervals, as appropriate. Changes from baseline were analysed using paired t tests or Wilcoxon signed-rank tests depending on the distributional assumptions. In response to peer review, post-hoc non-parametric sensitivity analyses using Wilcoxon signed-rank tests were performed for all main secondary outcome comparisons; corresponding p values are shown in separate sensitivity columns. COPM was assessed at baseline and at month 6 only. Results for COPM Performance and Satisfaction are given as average values. BFMDRS–Movement ratings were performed by a blinded rater who was not present at study visits and assessed anonymised video recordings at the end of the study. Effect sizes are reported as Cohen's d.

Abbreviations: GMFM-66 = Gross Motor Function Measure–66; CPCHILD = Caregiver Priorities and Child Health Index of Life with Disabilities; COPM = Canadian Occupational Performance Measure; BFMDRS = Burke–Fahn–Marsden Dystonia Rating Scale; AIMS = Abnormal Involuntary Movement Scale.

Table S3. BFMDRS-Disability scale of individual items

		**Baseline (at day 0)**	**Visit 2 (at day 90)**	**Visit 3 (at day 180)**
**Dressing**	N	n=12	n=11	n=11
	Median (IQR)	4·0 (4·0 to 4·0)	4·0 (3·0 to 4·0)	4·0 (4·0 to 4·0)
	Mean (CI)	3·8 (3·6 to 4·1)	3·8 (3·6 to 4·1)	3·8 (3·6 to 4·1)
**Eating and swallowing**	N	n=12	n=11	n=11
	Median (IQR)	2·0 (1·5 to 3·0)	1·0 (1·0 to 3·0)	1·0 (1·0 to 2·0)
	Mean (CI)	2·3 (1·7 to 3·0)	1·8 (1·1 to 2·5)	1·6 (1·1 to 2·2)
**Feeding**	N	n=12	n=11	n=11
	Median (IQR)	4·0 (3·0 to 4·0)	3·0 (2·0 to 4·0)	3·0 (2·0 to 4·0)
	Mean (CI)	3·5 (3·1 to 3·9)	3·2 (2·6 to 3·8)	3·0 (2·5 to 3·5)
**Handwriting**	N	n=12	n=11	n=11
	Median (IQR)	4·0 (3·0 to 4·0)	3·0 (3·0 to 4·0)	3·0 (3·0 to 4·0)
	Mean (CI)	3·7 (3·4 to 4·0)	3·3 (2·8 to 3·7)	3·3 (2·8 to 3·7)
**Hygiene**	N	n=12	n=11	n=11
	Median (IQR)	4·0 (4·0 to 4·0)	4·0 (4·0 to 4·0)	4·0 (4·0 to 4·0)
	Mean (CI)	3·9 (3·7 to 4·1)	3·9 (3·7 to 4·1)	3·9 (3·7 to 4·1)
**Speech**	N	n=12	n=11	n=11
	Median (IQR)	4·0 (4·0 to 4·0)	4·0 (4·0 to 4·0)	4·0 (4·0 to 4·0)
	Mean (CI)	4·0 (4·0 to 4·0)	3·9 (3·7 to 4·1)	3·8 (3·6 to 4·1)
**Walking**	N	n=11	n=10	n=10
	Median (IQR)	5·0 (4·0 to 5·0)	5·0 (5·0 to 5·0)	5·0 (4·0 to 5·0)
	Mean (CI)	4·6 (4·2 to 5·1)	4·7 (4·2 to 5·2)	4·5 (4·0 to 5·0)

Secondary clinical outcome measures assessed at baseline (day 0), visit 2 (day 90), and visit 3 (day 180). Data are presented for the modified full analysis set (n=12). Ordinal scale variables are shown as sample size (n), median (IQR) and mean (CI). BFMDRS = Burke–Fahn–Marsden Dystonia Rating Scale, IQR= Interquartile range, CI=95% confidence intervals.

Table S4. Group-level summary of baseline characteristics

		Eligible Participants (n=13)
**Age at inclusion (months) ***		59 (57 to 125)
**Sex**	Female	6 (46·2%)
	Male	7 (53·8%)
**Body measurements**	Height (cm)	108·5 (29·0)
	Body weight (kg)	18·9 (12·5)
	Body mass index (kg/m²)	14·7 (2·0)
**Dose groups according to age and weight**	≥ 6 months up to 6 years / 2 x 25mg	9 (69·2%)
	≥ 6 years up to 16 years / 3 x 25mg	2 (15·4%)
	≥ 16 years or body weight > 57 kg /3 x 50mg	2 (15·4%)
**Epilepsy**	All types	7/13 (53·8%)
**Types of epilepsy**	Focal onset	1/7 (14·2%)
	Generalized onset	4/7 (57·1%)
	Focal and generalized onset	2/7 (28·7%)
**Focal Motor and Nonmotor onset**	Motor onset: clonic	2/7 (28·7%)
	Nonmotor onset: behaviour arrest	2/7 (28·7%)
**Generalized Motor/Nonmotor onset**	Motor: Tonic-clonic	4/7 (57·1%)
	Motor Tonic	2/7 (28·7%)
	Nonmotor: Typical absence	1/7 (14·2%)
	Nonmotor: Atypical absence	1/7 (14·2%)
	Other or mixed seizure types	3/7 (42·8%)
**Dystonia**	Only with particular action	3 (23·1%)
	With many actions	8 (61·5%)
	On action of distant part of body or intermittently at rest	2 (15·4%)
**Speech impairment**	Speech impairment present	13 (100%)
	Anarthric	11 (84·6%)
	Dysarthric	12 (14·1%)
**Intellectual disability****		9 (69·2%)

Summary of demographic, genetic, and clinical baseline characteristics of eligible participants included in the full analysis set (n=13). Data are median (IQR), n (%), or mean (SD), collected at baseline (day 0). All characteristics refer to the status at study inclusion before initiation of zinc supplementation. Age is reported in months. Epilepsy classifications follow the International League Against Epilepsy terminology. *Age range at inclusion was 6–302 months. **Intellectual disability was not assessed in children younger than 2 years.

Table S5. Three categories of pathogenic Gαo based on their responses to zinc *in vitro*

Class	Zinc effect	Gαo variant	Reference
Class I	No effect on both GTP hydrolysis and GTPγS binding	wild-type	[1,2]
		L23P	[2]
		C215Y	[2]
		T241_N242insPQ	[14]
		S264F	[15]
		I344del	[2]
Class II	Increase GTP hydrolysis; no effect on GTPγS binding	K46R	[2]
		G203R	[1,2]
		R209C	[1,2]
		R209L	this study
		E246K	[1,2]
Class III	Effect on both GTP hydrolysis and GTPγS binding	K46N	[2]
		I55T	this study
		I56S	this study
		H57P	[2]
		P170R	[16]
		T182I	[2]
		R209H	[2]
		S229R	this study
		Y231C	[2]
		E237K	[2]
		F251L	[15]
		F288S	this study
		Y291N	[2]

Results of *in vitro* testing of pathogenic Gαo variants identified in participants in this study. Some variants have been reported previously; corresponding references are provided. At the molecular level, pathogenic Gαo variants can differentially disrupt GTP hydrolysis and GTPγS binding. *In vitro* zinc responsiveness testing classified variants into three categories based on their molecular effects (Classes I–III). Class I variants showed no detectable response to zinc supplementation. Class II variants demonstrated increased GTP hydrolysis in the presence of zinc. Class III variants showed both increased GTP hydrolysis and restoration of impaired GTPγS binding under zinc supplementation.

Table S6. Baseline epilepsy characteristics, antiseizure medication, and seizure course during zinc treatment

ID	GNAO1 variant (NM_020988.3)	Age at inclusion	Epilepsy phenotype	ASM at inclusion in (mg/kg/day)	Seizure burden before Inclusion	Seizure course during trial	Change in ASM during trial	Interpretation
1	c.625C>T (p.R209C)	25·2 y	Generalized and focal motor onset 7y;	Perampanel 0·16 mg/kg/day	Approximately 1 seizure/year	One seizure during the trial;	None	No clear change from baseline low seizure frequency.
6	c.626G>T (p.R209L)	4·8 y	Generalized motor onset with; tonic 3y;	No ASM	Approximately 1 seizure/year	No seizure during trial	None	No clear change from baseline low seizure frequency
7	c.674G>A (p.C225Y)	4·8 y	Generalized motor onset	OXC 10 mg/kg/d	Seizure free before inclusion	No seizure during trial	None	No change noted
9	c.687C>G (p.S229R)	1·3 y	EIDEE; focal motor onset at 5 days; EIMFS	LEV 66 mg/kg/d STM 11 mg/kg/d CBZ 0·27 mg/kg/d	1-3 seizures/day	Seizures remained frequent during the trial, decreasing to about 1/day or every 2 days	VPA initiated and increased to 48 mg/kg/day; STM increased to 13 mg/kg/day	Reduction in seizure burden occurred in the context of ASM escalation; no zinc-specific effect can be inferred
10	c.607G>A (p.G203R)	0·5 y	EIDEE; focal motor onset in the neonatal period, later generalized motor seizures	LEV 33 mg/kg/d LCM 15 mg/kg/d	Multiple seizures from day 3 of life; frequency reduction for 3 months before inclusion under ASM	No seizure during the trial	LEV reduced to 11 mg/kg/day and LCM to 10 mg/kg/day by end of trial also due to weight gain.	Seizure freedom was maintained during the trial; interpretation remains descriptive
12	c.164T>C (p.I55T)	10·8 y	Generalized motor onset at 3 years	No ASM	Seizure free for >2 years before inclusion	No seizure during the trial	None	No change noted
13	c.625C>T (p.R209C)	10·4 y	Generalized motor onset at 4 years	OXC 41·5 mg/kg/d Brivaracetam 1·38 mg/kg/d Clobazam 0·23 mg/kg/d	Seizure free for >2 years before inclusion	No seizure during the trial	None	No change noted

Summary of epilepsy phenotype, antiseizure medication (ASM) at study inclusion, baseline seizure burden, and seizure course during the 6-month trial period in participants with epilepsy. Where ASM was changed during the trial, seizure evolution is described in that clinical context. Because of the small number of affected participants, heterogeneous epilepsy phenotypes, and concomitant ASM adjustments in individual cases, these data are presented descriptively only.

Abbreviations: ASM = antiseizure medication; CBZ = carbamazepine; EIDEE = Early-infantile developmental and epileptic encephalopathy syndrome; EIMFS = epilepsy of infancy with migrating focal seizures; LCM = lacosamide; LEV = levetiracetam; OXC = oxcarbazepine; STM = sultiame; VPA = valproate/valproic acid.

Table S7. Baseline GNAO1-related disorder severity scores in trial participants

ID	Epilepsy	Movement Disorder	Gross motor function	Language development	Feeding	Total **GNAO1**-RD **severity score**	Severity category
**1**	1	1·5	2	1	0	5·5	Moderate
**2**	0	1·5	2	2	0	5·5	Moderate
**3**	0	3	4	2	1	10	Severe
**4**	0	2	3	2	1	8	Severe
**5**	0	2·75	1	1	0	4·75	Moderate
**6**	1	1·75	3	2	0	7·75	Moderate
**7**	0	0·5	3	2	1	6·5	Moderate
**8**	0	1·25	3	2	0	6·25	Moderate
**9**	3	2·5	4	2	1	12·5	Severe
**10**	2	0·75	3	2	0	7·75	Moderate
**11**	0	2·25	4	2	0	8·25	Severe
**12**	0	1·75	0	0	0	1·75	Mild
**13**	0·5	2·25	1	1	0	4·75	Moderate

Baseline GNAO1-related disorder (GNAO1-RD) severity scores in all participants enrolled in the ZINCGNAO1 trial. Domain scores for epilepsy, movement disorder, gross motor function, language development, and feeding are shown together with the total severity score and corresponding baseline severity category. Severity categories were defined as mild (0–3·9), moderate (4·0–7·9), and severe (≥ 8·0). The GNAO1-RD severity score was included for descriptive baseline characterization only and was not used as a prespecified study outcome.

Table S8. Longitudinal changes in secondary clinical outcome measures grouped by baseline severity score

Baseline GNAO1-RD severity score		Mild (N=1)			Moderate (N=8)			Severe (N=3)		
		Baseline (at day 0)	Visit 2 (at day 90)	Visit 3 (at day 180)	Baseline (at day 0)	Visit 2 (at day 90)	Visit 3 (at day 180)	Baseline (at day 0)	Visit 2 (at day 90)	Visit 3 (at day 180)
**GMFM-66**	N	n=1	n=1	n=1	n=8	n=7	n=7	n=3	n=3	n=2
	Median (IQR)	53·4	49·0	53·4	36·1 (26·7 to 43·7)	40·0 (27·3 to 51·9)	40·7 (31·2 to 51·1)	18·0 (16·0 to 26·0)	18·0 (14·8 to 26·0)	22·0 (18·0 to 26·0)
	Mean (CI)	53·4	49·0	53·4	35·5 (26·7 to 44·2)	39·6 (29·0 to 50·1)	40·0 (30·3 to 49·8)	20·0 (6·9 to 33·1)	19·6 (5·3 to 33·9)	22·0 (-28·8 to 72·8)
**CP-CHILD**	N	n=1	n=1	n=1	n=8	n=7	n=7	n=3	n=3	n=3
	Median (IQR)	59·0	42·0	49·6	59·5 (56·7 to 62·2)	60·2 (47·4 to 68·9)	59·1 (50·7 to 71·0)	51·4 (40·4 to 54·5)	46·5 (28·8 to 62·4)	47·8 (23·0 to 62·4)
	Mean (CI)	59·0	42·0	49·6	61·2 (55·0 to 67·5)	58·9 (46·5 to71·3)	61·1 (50·1 to 72·1)	48·8 (30·4 to 67·2)	45·9 (4·2 to 87·6)	44·4 (-5·1 to 93·9)
**COPM-Performance**	N	n=1	-	n=1	n=8	-	n=7	n=3	-	n=3
	Median (IQR)	2·4	-	3·8	2·5 (2·2 to 2·9)	-	4·0 (2·6 to 4·6)	2·4 (2·2 to 3·2)	-	3·2 (3·0 to 4·0)
	Mean (CI)	2·4	-	3·8	2·7 (1·9 to 3·4)	-	3·9 (2·5 to 5·4)	2·6 (1·3 to 3·9)	-	3·4 (2·1 to 4·7)
**COPM -Satisfaction**	N	n=1	-	n=1	n=8	-	n=7	n=3	-	n=3
	Median (IQR)	2·8	-	3·6	3·0 (2·0 to 3·4)	-	3·6 (2·6 to 6·8)	2·0 (1·4 to 3·6)	-	3·6 (2·8 to 4·4)
	Mean (CI)	2·8	-	3·6	2·8 (2·0 to 3·5)	-	4·3 (2·5 to 6·2)	2·3 (-0·5 to 5·2)	-	3·6 (1·6 to 5·6)
**BFMDRS-Disability**	N	n=1	n=1	n=1	n=8	n=7	n=7	n=3	n=3	n=3
	Median (IQR)	25·0	25·0	22·0	24·5 (22·5 to 27·0)	23·0 (21·0 to 24·0)	23·0 (21·0 to 24·0)	28·0 (27·0 to 28·0)	28·0 (25·0 to 28·0)	28·0 (25·0 to 28·0)
	Mean (CI)	25·0	25·0	22·0	24·8 (22·4 to 27·1)	22·9 (21·0 to 24·7)	22·3 (20·1 to 24·2)	26·7 (26·2 to 29·1)	27·0 (22·7 to 31·3)	27·0 (22·7 to 31·3)
**BFMDRS-Movement**	N	n=1	n=1	n=1	n=8	n=7	n=7	n=3	n=3	n=3
	Median (IQR)	43·5	43·5	43·5	55·5 (50·0 to 78·8)	52·5 (48·0 to 72·5)	53·5 (48·0 to 73·5)	61·0 (19·0 to 66·0)	65·0 (16·0 to 65·0)	61·0 (17·5 to 65·5)
	Mean (CI)	43·5	43·5	43·5	62·9 (46·7 to 79·2)	58·3 (40·9 to 75·6)	58·6 (40·3 to 76·8)	48·7 (-15·5 to 112·8)	48·7 (-21·6 to 118·9)	48·0 (-17·9 to 113·9)
**AIMS**	N	n=1	n=1	n=1	n=8	n=7	n=7	n=3	n=3	n=3
	Median (IQR)	8·0	8·0	8·0	7·5 (4·0 to 10·5)	7·0 (4·0 to 12·0)	8·0 (3·0 to 10·0)	2·0 (0·0 to 15·0)	0·0 (0·0 to 12·0)	0·0 (0·0 to 12·0)
	Mean (CI)	8·0	8·0	8·0	8·5 (2·4 to 14·6)	8·3 (1·9 to 14·7)	7·9 (1·9 to 13·8)	5·7 (-14·6 to 25·9)	4·0 (-13·2 to 21·2)	4·0 (-13·3 to 21·2)

Clinical outcome measures assessed at baseline (day 0), visit 2 (day 90), and visit 3 (day 180) for the patients grouped by baseline severity score (GNAO1-RD). Data are presented for the modified full analysis set (n=12). Continuous variables are shown as number of patients (n), median (IQR) and mean (95%-confidence interval). COPM was assessed at baseline and at month 6 only. Results for COPM-Performance and Satisfaction were given as average values. BFMDRS–Movement ratings were performed by a blinded rater who was not present at study visits and assessed anonymised video recordings at the end of the study.

Abbreviations: GMFM-66 = Gross Motor Function Measure–66; CPCHILD = Caregiver Priorities and Child Health Index of Life with Disabilities; COPM = Canadian Occupational Performance Measure; BFMDRS = Burke–Fahn–Marsden Dystonia Rating Scale; AIMS = Abnormal Involuntary Movement Scale.

4 Figures

Figure S1. Individual patient trajectories of secondary clinical outcome measures

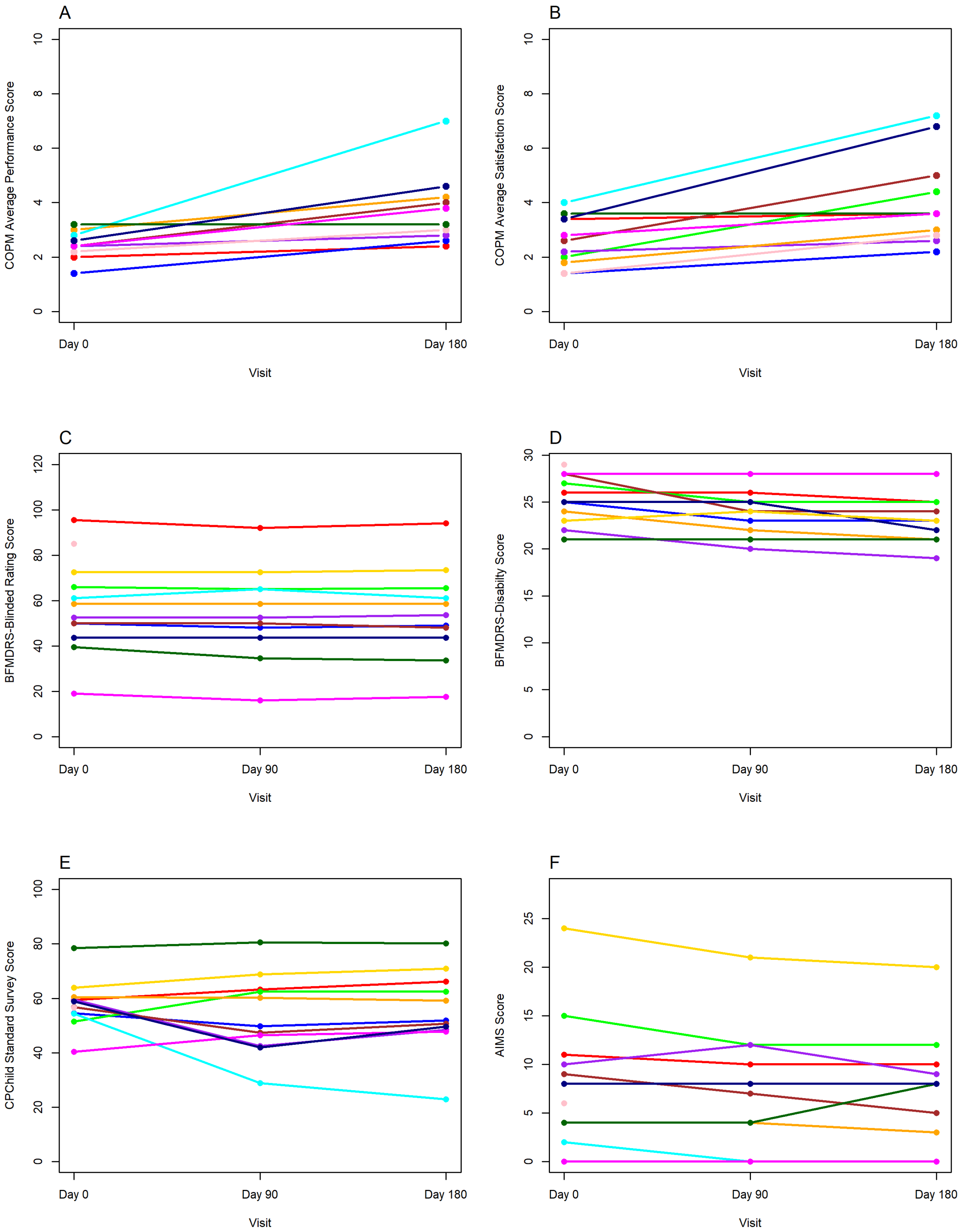

Individual patient trajectories of secondary clinical outcome measures during the 6–month zinc supplementation period. Measures were assessed at baseline (day 0), day 90, and day 180 during zinc supplementation. Data are shown for the modified full analysis set.
(A) Canadian Occupational Performance Measure (COPM) performance score.
(B) Canadian Occupational Performance Measure (COPM) satisfaction score.
(C) Burke–Fahn–Marsden Dystonia Rating Scale (BFMDRS) movement score, assessed by a blinded rater.
(D) Burke–Fahn–Marsden Dystonia Rating Scale (BFMDRS) disability score, assessed by an unblinded rater.
(E) Caregiver Priorities and Child Health Index of Life with Disabilities (CPCHILD).
(F) Abnormal Involuntary Movement Scale (AIMS).

Figure S2. Number of patients shifting between laboratory value categories at each visit.

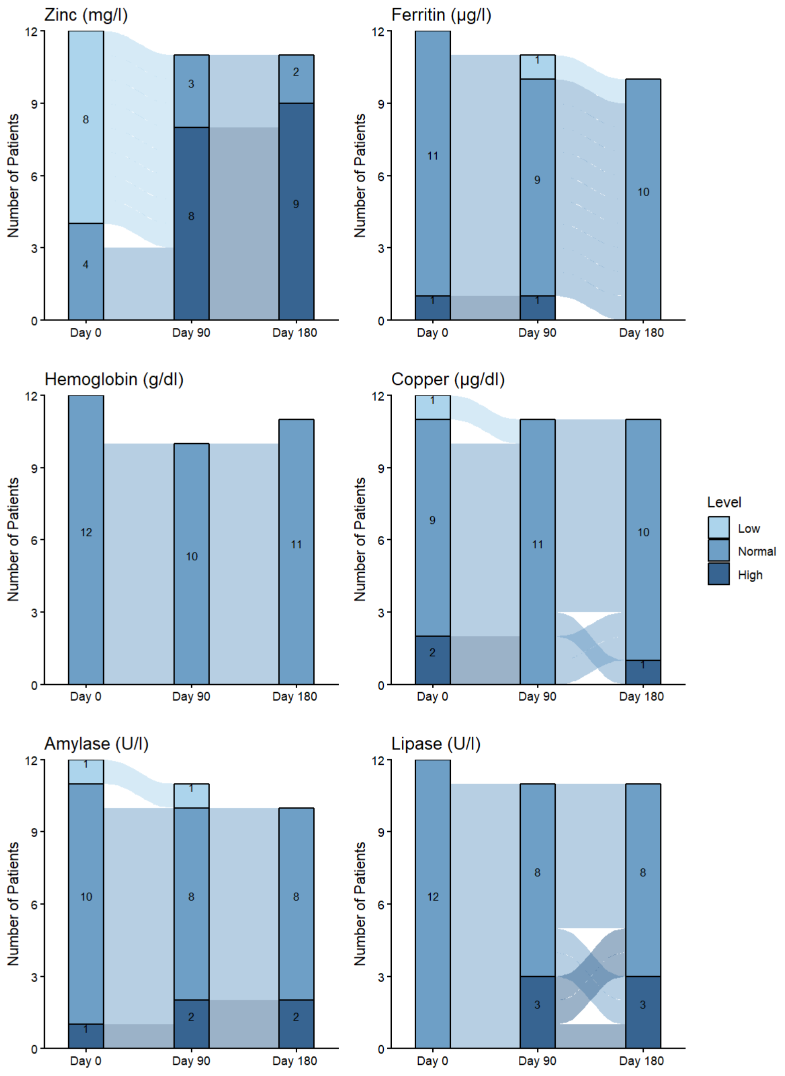

Distribution of patients across laboratory value categories (low, normal, and high) at each study visit. Laboratory results for key safety parameters (zinc, ferritin, haemoglobin, copper, amylase, and lipase) assessed in the safety analysis set are shown. Because reference ranges for these laboratory values vary according to age and sex, direct comparison of absolute values across participants is not appropriate. Therefore, results were categorised according to whether values were lower, normal, or higher than age- and sex-specific reference ranges at each visit.

Figure S3. Effects of zinc on novel pathogenic Gαo variants of participants

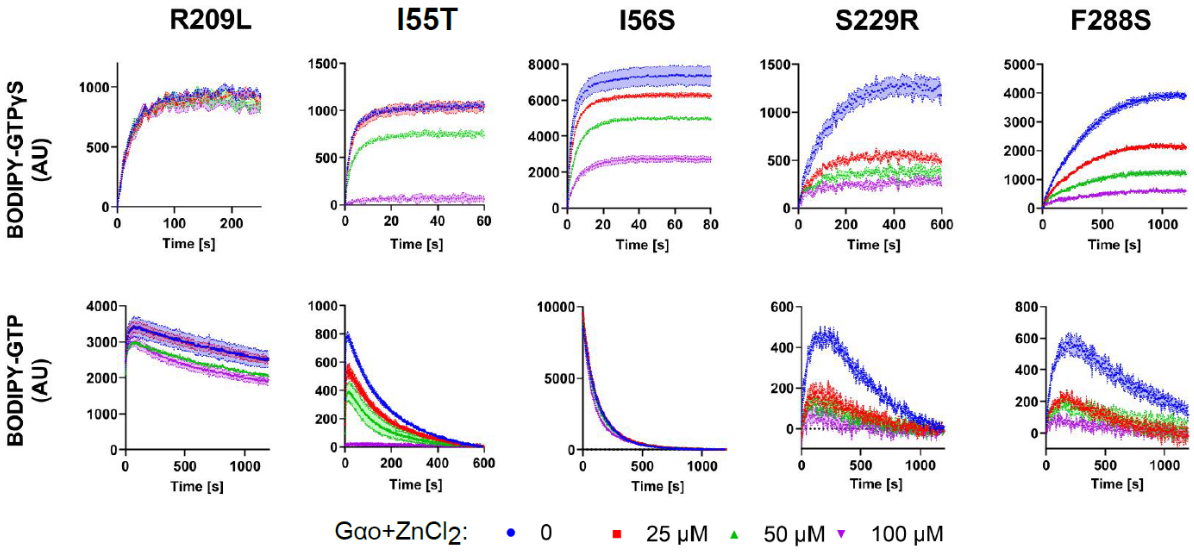

Graphical representation of the effects of zinc on pathogenic Gαo variants tested in this study. Recombinant Gαo (1 μM) was treated with $ZnCl_2$ (25–100 μM) before assessment using BODIPY–GTPγS binding and BODIPY–GTP hydrolysis assays. Based on a previous study, pathogenic variants were categorised into three classes: class I, showing no response to $ZnCl_2$ in either assay; class II, showing dose-dependent effects on BODIPY–GTP hydrolysis but not on BODIPY–GTPγS binding; and class III, showing dose-dependent effects in both assays. All variants tested were responsive to Zn^{2+}. Gαo[R209L] was classified as a class II variant, whereas I55T, I56S, S229R, and F288S were classified as class III variants. Data are presented as mean ± standard error of the mean (SEM; n=3 independent experiments).

5 References

1. Larasati YA, Savitsky M, Koval A, Solis GP, Valnohova J, Katanaev VL. Restoration of the GTPase activity and cellular interactions of Gα(o) mutants by Zn(2+) in GNAO1 encephalopathy models. *Sci Adv* 2022; **8**(40): eabn9350.
2. Larasati YA, Thiel M, Koval A, Silachev DN, Koy A, Katanaev VL. Zinc for GNAO1 encephalopathy: Preclinical profiling and a clinical case. *Med* 2025; **6**(1): 100495.
3. Russell DJ, Avery LM, Rosenbaum PL, Raina PS, Walter SD, Palisano RJ. Improved Scaling of the Gross Motor Function Measure for Children With Cerebral Palsy: Evidence of Reliability and Validity. *Physical Therapy* 2000; **80**(9): 873-85.
4. Pearson TS, Pons R, Ghaoui R, Sue CM. Genetic mimics of cerebral palsy. *Movement Disorders* 2019; **34**(5): 625-36.
5. Duran I, Stark C, Martakis K, Hamacher S, Semler O, Schoenau E. Reference centiles for the gross motor function measure and identification of therapeutic effects in children with cerebral palsy. *Journal of Evaluation in Clinical Practice* 2019; **25**(1).
6. Rosenbaum PR, Rubin DB. The central role of the propensity score in observational studies for causal effects. *Biometrika* 1983; **70**(1): 41-55.
7. Austin PC. An introduction to propensity score methods for reducing the effects of confounding in observational studies. *Multivariate Behavioral Research* 2011; **46**(3).
8. Duran I, Schafmeyer L, Lentzen B, et al. Longitudinal reference centiles for the Gross Motor Function Measure-66 in children and adolescents with cerebral palsy. *Dev Med Child Neurol* 2025.
9. Austin PC. The performance of different propensity-score methods for estimating differences in proportions (risk differences or absolute risk reductions) in observational studies. *Statistics in Medicine* 2010; **29**(20).
10. Rassen JA, Shelat AA, Myers J, Glynn RJ, Rothman KJ, Schneeweiss S. One-to-many propensity score matching in cohort studies. *Pharmacoepidemiology and Drug Safety* 2012; **21**: 69-80.
11. Rubin DB, Rosenbaum PR. Constructing a Control Group Using Multivariate Matched Sampling Methods That Incorporate the Propensity Score. *Taylor & Francis* 2012.
12. Ho D, Imai K, King G, Stuart EA. MatchIt: Nonparametric Preprocessing for Parametric Causal Inference. *Journal of Statistical Software* 2011; **42**: 1-28.
13. Torchiano M. effsize: Efficient Effect Size Computation. 2020.
14. Koval A, Larasati YA, Savitsky M, et al. In-depth molecular profiling of an intronic GNAO1 mutant as the basis for personalized high-throughput drug screening. *Med* 2023; **4**(5): 311-25.e7.
15. Solis GP, Danti FR, Larasati YA, et al. Clinical-molecular profiling of atypical GNAO1 patients: Novel pathogenic variants, unusual manifestations, and severe molecular dysfunction. *Genes Dis* 2025; **12**(5): 101522.
16. Larasati YA, Solis GP, Koval A, et al. Clinical Cases and the Molecular Profiling of a Novel Childhood Encephalopathy-Causing GNAO1 Mutation P170R. *Cells* 2023; **12**(20).

UNIKLINIK KÖLN
Institut für Medizinische Statistik und Bioinformatik

AG Medizinische Statistik
Prof. Dr. Achim Tresch
(Kommissarische Leitung)

Statistical Analysis Plan

Study title:	Prospective pilot trial to address the feasibility and safety of treatment with oral zinc in GNAO1 associated disorders
Study code:	ZincGNAO1
Indication:	GNAO1 associated disorders
Investigational intervention:	An oral therapy with zinc (Zinc acetate dihydrate in age-adapted dosage ranging from 50mg to 150mg Zn^{2+} per day according to the recommended dosage in Wilson‘s disease.)
Comparator:	n.a.
Sponsor (or representative):	University of Cologne<br>Albertus-Magnus-Platz<br>50923 Cologne<br>Germany
Financial support:	Supported by German parent association<br>GNAO1-Gemeinsam nicht allein e.V
Protocol identification:	Uni-Koeln-5275
Development phase (if appropriate):	Phase II
Principal investigator:	Dr. Moritz Thiel,<br>Children’s Hospital<br>University Hospital Cologne<br>Kerpener Str. 62<br>50937 Cologne
Statistics:	Institute for Medical Statistics and Computational Biology (IMSB)<br>Kerpener Str. 62<br>50937 Cologne
SAP author(s):	Dr. Petra Schiller, Jithmi Weliwitage

CONFIDENTIAL

SAP version:1.0, final Date: 2025-07-22

Approved by

Dr. Moritz Thiel,<br>Principal Investigator	Place and date	Signature
Dr. Petra Schiller,<br>Statistician (responsible)	Place and date	Signature
Jithmi Weliwitage,<br>Statistician (representative)	Place and date	Signature

Content

1 Background 4
1.1 Trial objective 4
1.2 Trial design 4
1.3 Timing of Analyses 5
1.4 Sample size 5
2 Analysis populations 5
2.1 Definitions 5
2.2 Major protocol violations / Withdrawals 6
3 Trial centres 6
4 Handling of missing values and outliers 6
4.1 Missing values 6
4.2 Outliers 6
5 Statistical methods and analysis variables 6
5.1 Patient / Subject Disposition 6
5.2 Demography and baseline characteristics 6
5.3 Primary analysis 7
5.3.1 Primary variables 7
5.3.2 Secondary variables 7
5.4 Secondary analysis 10
5.5 Planned subgroup analyses 10
5.6 Planned further analyses 11
6 Data problems 11
7 Software 11
8 References 11

List of Figures

Figure 1 ZincGNAO1 study flow chart 5
Figure 2 Time plan of the study 5

List of abbreviations

ADR	Adverse Drug Reaction
AE	Adverse Event
AIMS	Abnormal Involuntary Movement Scale
BFMDRS	Burke-Fahn-Marsden Dystonia Rating Scale
COPM	Canadian Occupational Performance Measure
FAS	Full analysis set
DBS	Deep Brain Stimulation
GMFM-66	Gross Motor Function Measure
IMP	Investigational Medicinal Product
ITT	Intention-to-treat
PP	Per protocol
SAE	Serious Adverse Event
SAR	Serious Adverse Reaction
SUSAR	Suspected Unexpected Serious Adverse Reaction

1 Background

1.1 Trial objective

To assess the safety and feasibility of the administration of zinc in patients affected by GNAO1 associated disorders and to document potential changes of general Motor Skills, Level of alertness with improved day/night rhythm and reduction of dyskinesia for 6 months of treatment.

Primary objective:

- To assess the feasibility of administration of zinc by the actual days that zinc was taken in the scheduled right dosage at least on 80% of the days.
- To assess the safety by measuring regular evaluation of the AEs defined in section 6.1 in the protocol [1].

Secondary objectives:

- Level of motor-skills measured by change of Gross-motor function measure (GMFM-66)
- Quality of life measured by CP-Child questionnaire for the caregivers and Canadian occupational performance measure (COPM)
- Level of Dystonia measured by change of Burke-Fahn-Marsden Dystonia Rating scale (BFMDRS-Movement Score, blinded rater; BFMDRS-Disabilty Score, unblinded rater)
- Level of dyskinesia measured by Abnormal involuntary movement scale (AIMS) and a Movement log for parents
- Changes in general behaviour including level of alertness, better sleep
- Changes in Seizure logs (times, duration, frequency)
- Changes of Motor Skills and level of dyskinesia in correlation to specific variant in GNAO1
- Serum controls of zinc to measure efficacy of oral zinc administration
- Serum ferritin and copper to detect potential deficiencies, caused by regular zinc administration and therefore reduced uptake of iron and copper
- Analyse of the microbiome in stool

1.2 Trial design

This is a phase II, single arm, open-label pilot trial to assess the safety and feasibility of oral zinc medication in age dependent doses for 6 months in patients with GNAO1 associated disorders.

The trial will be open as monocentre study at the Children's Hospital of Cologne.

The outcome variables will be assessed at:

- Baseline visit (T=0)
- Post start,10 days ± 7 days (T=1)
- Months 3 visit (T=2)
- Months 6 visit (T=3)
- End of trial,30 days ± 10 days after last follow up (T=4)

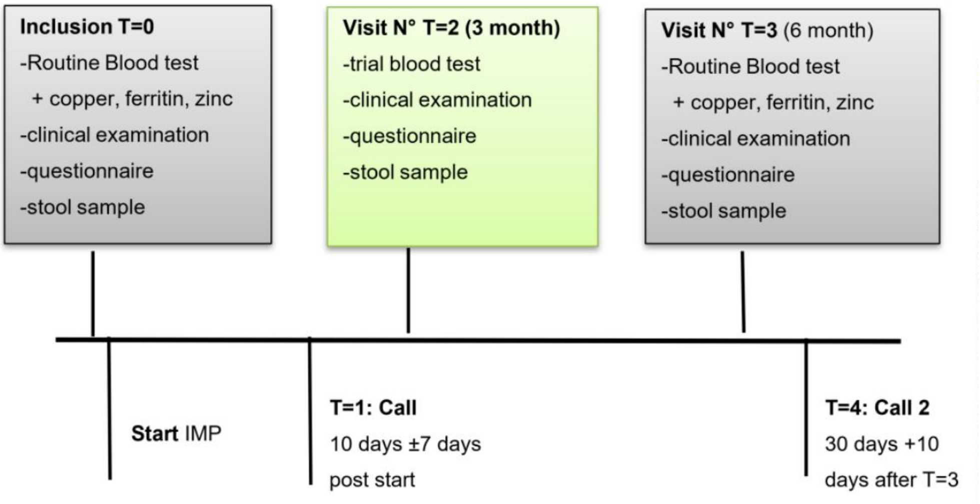

Figure 1: ZincGNAO1 study flow chart

1.3 Timing of Analyses

The study will end 30 ± 10 days after the last visit of the last patient. In total, a duration of 7 months ± 10 days is estimated for an individual patient. Final data entry, monitoring, data checks, queries and data completion with data base closure will then take place, and the final analysis will be performed.

First patient first visit (FPFV):	Q II 2024
Last patient first visit (LPFV):	Q II 2025
Last patient last visit (LPLV):	Q IV 2025
End of trial:	Q IV 2025
Final study report:	Q I 2026

Figure 2: Time plan of the study

1.4 Sample size

For this pilot study, the sample size was set to 12 participants, which seems feasible to be recruited within a limited time frame, but still to allow to answer the question of feasibility of oral zinc treatment and to give first signals on safety.

From a statistical perspective, the sample size n=12 locally minimizes the imprecision of the estimate of probability of response i.e. the half-width of the exact Clopper-Pearson 95% confidence interval assuming a true response probability of 80% (see clinical protocol V02, page 45; calculated with R: A Language and Environment for Statistical Computing, R Foundation for Statistical Computing, Vienna, Austria; binomtest)

2 Analysis populations

All analyses will be conducted on two trial populations (FAS and PP), expect from safety endpoints which will be analysis based on safety population (VAS).

2.1 Definitions

Full analysis set (FAS)

This dataset includes all trial participants who have been enrolled into the trial with the intention to take (not necessarily taken) the trial medication.

Per-protocol set (PP)

The per-protocol population is defined by the trial participants which took the scheduled dosage of the IMP at least on 80% of the scheduled days.

Safety set (VAS)

The safety analysis is based on the safety population (valid for safety, VAS), which includes all trial participants who have been enrolled, have started trial medication and have taken at least one dose.

2.2 Major protocol violations / Withdrawals

Serious protocol violations will be described on a case-by-case basis.

3 Trial centres

Site	Clinic
01 Cologne	Children’s Hospital, University Hospital Cologne

4 Handling of missing values and outliers

4.1 Missing values

No attempt will be made to impute missing data except movement log. Missing values of movement log will be imputed by the “linear interpolation imputation method” or substituted by the mean of the proceeding and the following value.

4.2 Outliers

No outlier analysis will be done.

5 Statistical methods and analysis variables

5.1 Patient / Subject Disposition

All patients enrolled will be accounted for. fulfilment of inclusion/exclusion [1]. Patients discontinuing study medication or not completing the study will be listed along with the reason for their premature discontinuation. Patients excluded from any analysis population will be listed, with frequencies and reasons for exclusion. A CONSORT flow chart will be prepared.

5.2 Demography and baseline characteristics

Demographic and baseline characteristics will be tabulated and summarized, as applicable, by mean, standard deviation, median, interquartile range, minimum and maximum or count and percentage.

Examined demographics include age (age at onset of symptoms, age when first diagnosed) and sex. Baseline characteristics comprise: genetic variants, symptoms, current medication with dosages, current DBS settings.

5.3 Primary analysis

5.3.1 Primary variables

- Feasibility
  Administration of zinc therapy (total dose, dose per day) and number of days that zinc was taken in the right dose
- Safety
  Incidence of adverse events and adverse drug reactions

The measures of **feasibility**, i.e. administration of zinc therapy (total dose, dose per day) will be descriptively summarized using median, maximum/minimum, mean, standard deviation and quartiles.

Number of days for each patient on which zinc was taken in the right dosage out of the total scheduled days will be calculated and summarized using percentage, median, maximum/minimum, mean, standard deviation and quartiles.

The proportion of patients who took at least 80% of zinc medication will be given with 95% confidence interval. Administration is considered feasible, if a response rate of at least 80% is observed.

The **safety** analysis will be assessed by evaluation of AEs. The number of events and percentages of (S)AEs, SARs, SUSARs and ADRs will be reported. Further, AEs and ADRs will be summarized by system organ class, seriousness, causal relationship, severity and outcome and case listings will be given. Moreover, side effects and reasons for not giving the medication in the recommended way will be summarized and case listings will also be given.

5.3.2 Secondary variables

Summary statistics of secondary variables will be tabulated and given in figures as descripted below. No multiplicity adjustment for alpha is planned. P-values are interpreted two-sided. Values below 0.05 are considered statistically significant.

Analysis and presentation of continuous variables

- Level of motor-skills
  - as measured by the Gross-Motor-Function messure-66 (GMFM-66) at visit 0, 2 and 3. The level (numerical value) reached at visit 2 and 3 will be compared to the level at baseline.
- Quality of life
  - as reported by the Caregiver Priorities & Child Health Index of Life with Disabilities (CP-Child) Questionnaire for the caregivers (Visit 0, 2 and 3). The level (numerical value) reached at visit 2 and 3 will be compared to the level at baseline.
  - as reported in the Canadian Occupational Performance Measure (COPM) (Visit 0 and 3). The Performance Score (numeric value) and Satisfaction Score (numeric value) reached at visit 3 will be compared to the Score at baseline.

The occupational performance problems of all participants will be described in groups.

- Serum level of zinc
  - measured at visit 0, 2, 3
    descriptive analyses of the level at baseline, at visit 2 and at visit 3 and of the changes in comparison to baseline
- Serum level of ferritin and copper
  - measured at visit 0, 2, 3
    descriptive analyses of the level at baseline, at visit 2 and at visit 3 and of the changes in comparison to baseline
  - descriptive analyses of the need for ferritin or copper substitution

The above listed continuous secondary variables will be reported for each visit using descriptive statistics (mean, standard deviation, median, interquartile range, minimum and maximum). Change from baseline (visit 0) to follow-up visits will be summarized by mean, standard deviation, median, interquartile range and 95% confidence intervals will be given.

Depending on data distribution (normality) paired statistical tests, t-test or Wilcoxon signed-rank test will be used to evaluate statistical significance of within-subject changes between visits.

Mock table 1: Descriptive analysis

Measure	Visit	Mean (SD)	Median (IQR)	Min-Max	Change from baseline	Mean (SD) or median (IQR)	95% CI	p-value
	Visit 0							
	Visit 2				Visit 2 - visit 0			
	Visit 3				Visit 3 - visit 0			

Mock Figure 1: Time course

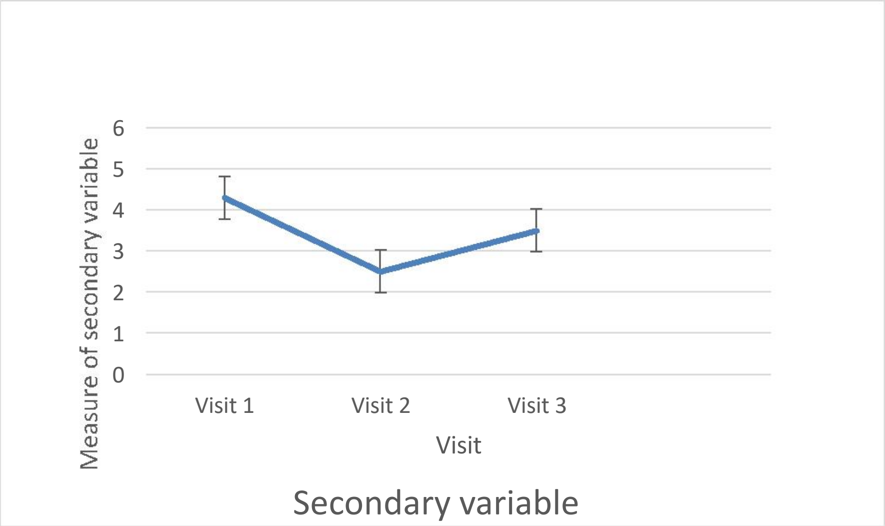

Analysis and presentation of movement disorder

- Level of movement disorder
  - as measured by Change of Burke-Fahn-Marsden Dystonia Rating scale (BFMDRS-Movement Score, blinded rater; BFMDRS-Disabilty Score, unblinded rater). The scale (numerical value) at visit 2 and 3 will be compared to the scale at baseline similar to mock table 1.
  - as measured by the Abnormal involuntary movement scale (AIMS). The scale (numerical value) at visit 2 and 3 will be compared to the scale at baseline similar to mock table 1.
  - as given in the movement log (reported by the parents):
    The AUC of the percentages of involuntary movements, dystonia or choreoathetosis of the awake daytimes during the first two weeks after baseline will be compared to the AUC of the two weeks before visit 2 and to the two weeks before visit 3.
    Missing values will be imputed by the „linear interpolation imputation method" (*56*). A missing value will be substituted by the mean of the proceeding and the following value.

The AUC of the percentages of involuntary movements, dystonia or choreoathetosis of the awake daytimes will be summarized similar to mock table 1 for the corresponding visits (2 weeks after visit 1,2 weeks before visit 2 and 2 weeks before visit 3).

Analysis of change in behaviour, sleep and seizure

- Changes in general behaviour including level of alertness
  - As reported by the care givers in the questionnaire. The parents have to classify (classify as e.g. "much improved"; "improved," "stable", "declined" or "much declined" or via a rating scale). General behaviour including level of alertness during the first two weeks after baseline will be compared to the two weeks before visit 2 and to the two weeks before visit 3.

The average rating score (1-10 Likert scale) of general behaviour will be summarized similar to mock table 1 table at corresponding visits (2 weeks after visit 1,2 weeks before visit 2 and 2 weeks before visit 3).

- Change in sleep
  - As reported by the parents sleep time and sleep interruptions during the first two weeks after baseline will be compared to the two weeks before visit 2 and to the two weeks before visit 3The number of sleeping hours and number of days with interruptions will be summarized similar to Example statistical output 1 table at corresponding visits (2 weeks after visit 1,2 weeks before visit 2 and 2 weeks before visit 3).

- Changes in frequency, time and duration of seizures
  - as reported by the parents
    frequency, time duration and classification according to ILEA 2017 of seizures during the first two weeks after baseline will be compared to the two weeks before visit 2 and to the two weeks before visit 3.

Seizure data will be summarized by in total and by types of Epilepsy using median and interquartile range for the frequency and duration measures.

Mock table 2: Descriptive analysis for seizure data

Visit	Seizure type	N of patients	Median (IQR) frequency	Median (IQR) duration	Change	Frequency		Duration	
						Median (IQR)	p-value	Median (IQR)	p-value
2 weeks after visit 1 (V1)									
2 weeks before visit 2 (V2)					2 weeks before V2 - 2 weeks after V1				
2 weeks before visit 3 (V3)					2 weeks before V3 - 2 weeks after V1				

5.4 Secondary analysis

The described analyses will be repeated with the per-protocol population (sensitivity analysis).

5.5 Planned subgroup analyses

No gender differences are expected, however a subgroup analysis for gender is planned. We expect a male: female ratio of 1:1. All major efficacy and safety variables will be analysed by gender. Furthermore, the variants which occur most often will be analysed separately. The individual results will be put in correlation to the specific variant of the patient to see if the outcome is influenced by the specific variant if feasible. No other subgroup analyses are foreseen but may be explored ad hoc. The results of subgroup analyses will be summarized similar to mock table 3 and mock figure 2.

Mock table 3: Descriptive analysis of sub groups

Measure	Visit	Group	Mean (SD)	Median (IQR)	Min-Max	Change from baseline	Mean (SD) or median (IQR)	95% CI	p-value
	Visit 0	Group 1							
		Group 2							
	Visit 2	Group 1				Visit 2 - visit 0			
		Group 2				Visit 2 - visit 0			
	Visit 3	Group 1				Visit 3 - visit 0			
		Group 2				Visit 3 - visit 0			

Mock Figure 2: Comparison of sub groups

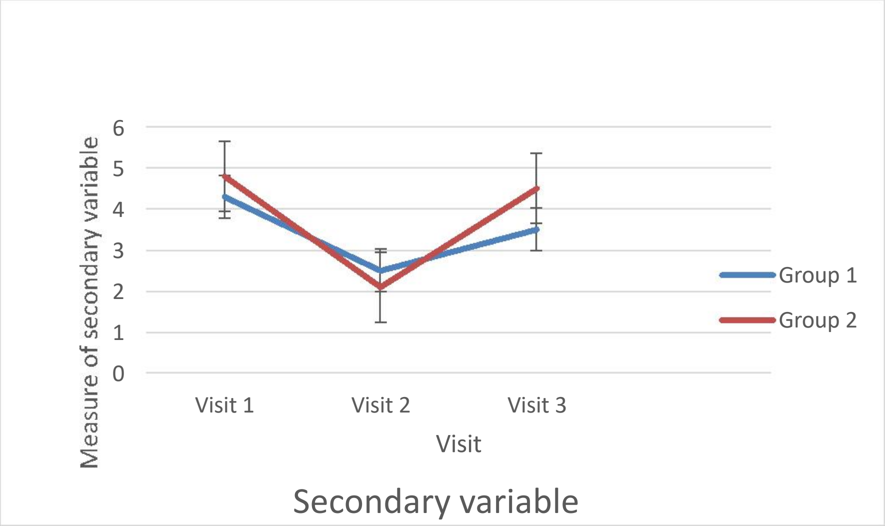

5.6 Planned further analyses

- Microbiome: descriptive analyses as appropriate

Microbiome analyses will be done by M. Thiel and/or co-workers.

6 Data problems

Not expected. If any, circumstances and treatment will be documented in the final analysis report.

7 Software

Data preparation and statistical analyses will be performed using R-version 4.4.0 (R Foundation for Statistical Computing, Vienna, Austria), SPSS Statistics-version 29.0.1.1 (IBM Corp., Armonk, NY, USA) or SAS version 9.4 (SAS Institute Inc., Cary, NC, USA) or higher.

8 References

[1] Clinical Study Protocol Version 1.2 (2025-03-24).
