# Supplementary material for: Safety and feasibility of oral zinc for patients with GNAO1-related disorders (ZINCGNAO1): an open-label, single-arm, single-centre, pilot trial in Germany
Source: eClinicalMedicine. 2026 Jul 23;98:104092. doi: 10.1016/j.eclinm.2026.104092 (PMC13427569; doi:10.1016/j.eclinm.2026.104092)
Supplement: Translated Abstract [file mmc2.docx]

*The following translations in German were submitted by the authors and we reproduce them as supplied. They have not been peer reviewed. Our editorial processes have only been applied to the original abstract in English, which should serve as reference for this manuscript.*

**Safety and feasibility of oral zinc for patients with *GNAO1*-related disorders (ZINCGNAO1): an open-label, single-arm, single-centre, pilot trial in Germany**

Hintergrund: GNAO1-assoziierte Erkrankungen (GNAO1-RD), verursacht durch pathogene Varianten im GNAO1-Gen, umfassen ein breites neurologisches Spektrum mit Bewegungsstörungen und/oder Epilepsie sowie meist globaler Entwicklungsverzögerung und intellektueller Beeinträchtigung. Bisher stehen nur symptomatische Behandlungsoptionen zur Verfügung. Präklinische Daten deuten darauf hin, dass Zink die GTP-Hydrolyse und zelluläre Interaktionen dysfunktionaler Gαo-Proteine teilweise wiederherstellen kann. Ziel der ZINCGNAO1-Studie war es, Sicherheit und Durchführbarkeit einer oralen Zinksupplementierung bei Patientinnen und Patienten mit GNAO1-RD zu untersuchen.

Deutsche Zusammenfassung

Hintergrund: GNAO1-assoziierte Erkrankungen (GNAO1-RD), verursacht durch pathogene Varianten im GNAO1-Gen, das für das Gαo-Protein kodiert, umfassen ein breites phänotypisches Spektrum mit Bewegungsstörungen und/oder Epilepsie und sind typischerweise mit Entwicklungsverzögerung und intellektueller Beeinträchtigung assoziiert. Derzeit stehen nur symptomatische Behandlungen zur Verfügung. Zink stellt einen potenziell krankheitsmodifizierenden Therapieansatz dar, da es die Guanosintriphosphat-Hydrolyse und zelluläre Interaktionen dysfunktionaler Gαo-Proteine wiederherstellen kann. Ziel von ZINCGNAO1 war es, die Sicherheit und Durchführbarkeit einer oralen Zinksupplementierung bei pädiatrischen Patientinnen und Patienten mit GNAO1-RD zu untersuchen.

Methoden: ZINCGNAO1 war eine sechsmonatige, offene, fix dosierte, monozentrische Pilotstudie in Deutschland. Einschlusskriterien waren ein Alter von 6 Monaten bis 30 Jahren, eine genetisch bestätigte pathogene GNAO1-Variante, mindestens ein Hauptmerkmal von GNAO1-RD (Bewegungsstörung, Hypotonie, Epilepsie oder globale Entwicklungsverzögerung) sowie ein Gross Motor Function Measure-66 (GMFM-66)-Wert von höchstens 75. Zinkacetat-Dihydrat wurde altersabhängig oral in hohen Dosen verabreicht. Primäre Endpunkte waren Sicherheit, erfasst durch Monitoring unerwünschter Ereignisse und Laboruntersuchungen, sowie Durchführbarkeit, definiert als Therapieadhärenz an mindestens 80 % der Studientage. Sekundäre Endpunkte umfassten Veränderungen der Schwere der Bewegungsstörung, motorischen Funktion, Lebensqualität, Epilepsie sowie patienten- oder betreuerberichteter Endpunkte; diese Analysen waren explorativ. Die Studie wurde bei ClinicalTrials.gov (NCT06412653) und im EU Clinical Trials Register (2024-512735-72-00) registriert und ist abgeschlossen.

Ergebnisse: Zwischen dem 2. August 2024 und dem 27. Januar 2025 wurden 13 Teilnehmende mit 11 unterschiedlichen genetischen Varianten eingeschlossen; 11 schlossen die Nachbeobachtung der Studie ab. Das mittlere Alter bei Einschluss betrug 8,0 Jahre (Spannweite 0,5 bis 25,1). Die Gesamt-Durchführbarkeit der Behandlung lag bei 84,6 % (11/13). Es traten keine behandlungsbedingten schwerwiegenden unerwünschten Ereignisse auf, und das Labormonitoring ergab während der sechsmonatigen Behandlungsphase keine klinisch relevanten Sicherheitsbedenken. Die Schwere der Bewegungsstörung zeigte über den sechsmonatigen Behandlungszeitraum nur geringe Veränderungen. Explorative Analysen sekundärer Endpunkte zeigten Anstiege der GMFM-66-Werte von Studienbeginn (Mittelwert 33,1 [SD 12,8]) bis 6 Monate (Mittelwert 37,8 [SD 12,8]; nominal p=0,006) sowie der Leistungs- und Zufriedenheitswerte des Canadian Occupational Performance Measure (nominal p=0,003 bzw. nominal p=0,002). Explorative Kontextanalysen der GMFM-66-Daten, einschließlich eines Vergleichs mit publizierten Referenzperzentilen und einer Propensity-Score-gematchten Vergleichsanalyse mit Personen mit Zerebralparese, wurden durchgeführt, um diese Veränderungen weiter einzuordnen.

Interpretation: Unsere vorläufigen Ergebnisse zeigen, dass eine orale Zinksupplementierung in dieser GNAO1-RD-Population sicher und durchführbar war. Explorative sekundäre Endpunkte deuteten auf mögliche Verbesserungen der motorischen Funktion und alltagsrelevanter Aktivitäten hin. Größere multizentrische randomisierte oder Delayed-Start-Studien sind erforderlich, um die Wirksamkeit zu untersuchen.

Finanzierung: GNAO1 – Gemeinsam nicht allein e.V.

Schlüsselwörter: GNAO1-assoziierte Erkrankung; Zinkacetat; Sicherheit und Durchführbarkeit; pädiatrische Bewegungsstörungen; entwicklungsbedingte und epileptische Enzephalopathie; Präzisionsmedizin
