# Supplementary material for: Safety and feasibility of oral zinc for patients with GNAO1-related disorders (ZINCGNAO1): an open-label, single-arm, single-centre, pilot trial in Germany
Source: eClinicalMedicine. 2026 Jul 23;98:104092. doi: 10.1016/j.eclinm.2026.104092 (PMC13427569; doi:10.1016/j.eclinm.2026.104092)
Supplement: Protocol [file mmc3.pdf]

Universität zu Köln

Trial Protocol

Prospective pilot trial to address the feasibility and safety of treatment with oral zinc in GNAO1 associated disorders

(ZINCGNAO1)

Sponsor

University of Cologne
Albertus-Magnus-Platz
50923 Köln
Germany

Principal Investigator:
Dr. Moritz Thiel
Children's Hospital
University Hospital Cologne
Kerpener Strasse 62
50937 Cologne
Germany

Trial protocol code: Uni-Koeln-5275

EU CT Nr. **2024-512735-72-00**

Version 1.2 dated 24.03.2025

The information in this trial protocol is strictly confidential. It is for the use of the sponsor, principal investigator, trial personnel, ethics committee, the authorities, and trial participants only. This trial protocol may not be passed on to third parties without the express agreement of the sponsor or the Principal Investigator (PI)

I. Signatures

Dr. Moritz Thiel (Principal investigator)<br>On behalf of the sponsor<br>University of Cologne	Signature	Date
PD Dr. Kyriakos Martakis<br>(investigator and clinical support)<br>University of Cologne	Signature	Date
Dr. Barbara Hero<br>(clinical project management and administrative support)<br>Klinisches Studienzentrum Pädiatrie<br>University of Cologne	Signature	Date
Dr. Petra Schiller<br>Institute of Medical Statistics and Computational Biology (IMSB)<br>University of Cologne	Signature	Date

Synopsis

Sponsor:	University of Cologne<br>Represented by:<br>Dr. Moritz Thiel<br>Children's Hospital<br>University Hospital Cologne<br>Kerpener Strasse 62<br>50937 Cologne<br>Germany
Principal Investigator	See above
Title of the clinical trial:	Prospective pilot trial to address the feasibility and safety of treatment with oral Zinc in GNAO1 associated disorders
Collaborating Laboratory	Translational Research Center in Oncohaematology, Department of Cell Physiology and Metabolism,<br>University of Geneva,<br>CMU Rue Michel-Servet 1<br>CH-1211 Genève
Indication:	GNAO1 associated disorders
Phase:	Phase II, therapeutic-exploratory
Type of trial, trial design, methodology:	Single-arm, open-label pilot trial
Number of participants:	12
Primary trial objective:	To investigate feasibility and safety of an oral therapy with zinc in patients affected by GNAO1 associated disorders and to document potential changes of general Motor Skills, Level of alertness with improved day/night rhythm and reduction of dyskinesia.

<table>
<tr>
<td>Study end points:</td>
<td>
Primary end point

Feasibility of treatment with oral zinc demonstrated by taking the study medication in 80% of the days
Safety of daily administered zinc in GNAO1 by regular evaluation of the AEs as defined in chapter 6.1.

Secondary end points

Level of motor-skills measured by change of Gross-motor function measure (GMFM-66)
Quality of life measured by CP-Child questionnaire and COPM
Level of Dystonia measured by change of Burke-Fahn-Marsden Dystonia Rating scale (BFMDRS)
Level of dyskinesia measured by Abnormal involuntary movement scale (AIMS) and a Movement log for parents
Changes in general behaviour including level of alertness and sleep
Changes in Seizure logs (duration, frequency)
Changes of Motor Skills and level of dyskinesia in correlation to specific variant in GNAO1
Serum controls of zinc to measure efficacy of oral zinc administration
Serum ferritin and copper to detect potential deficiencies, caused by regular zinc administration and therefore reduced uptake of iron and copper
Analyze of the microbiome in stool

</td>
</tr>
<tr>
<td>Diagnosis and Principal inclusion and exclusion criteria:</td>
<td>
Medical condition or disease to be investigated:

GNAO1-associated disorders

Principal inclusion criteria:

GNAO1 associated neurological disorder, documented by either

proven pathogenic or likely pathogenic mutation in GNAO1 or
a variant of unknown significance in GNAO1 and clinical symptoms likely to be consistent with GNAO1 as determined by the investigators

and at least one of the common symptoms of GNAO1: Movement disorder (Dystonia, Chorea, Ataxia, clonic), central muscular hypotonia, epilepsy, global developmental delay
Age: 6 month – 30 years
GMFM ≤ 75
written informed consent prior to any trial-related procedure (according to age and status of psycho-intellectual development) by parents or legal guardian
stable on following concomitant treatments for at least 3 months prior to trial inclusion: anti-seizure drugs (ASD); baclofen, Deep brain stimulation settings

</td>
</tr>
</table>

	**Principal exclusion criteria:**<br>• Treatment of Zinc in the last 4 months before inclusion<br>• known other genetic variants that are known to cause symptoms like observed in GNAO1-related disorders, additional to the proven GNAO1 mutation<br>• implantation of Deep brain stimulation planned during the duration of the trial, i.e. in the six months after inclusion<br>• start of intrathecal baclofen therapy planned during the duration of the trial, i.e. in the six months after inclusion<br>• Known allergy/hypersensitivity to the scheduled trial drug<br>• Concomitant participation in other clinical drugs with investigational drugs or with competing interventions<br>• sexually active patients who are not willing to use/ not using a highly effective contraception method with a pearl-index < 1. Sexually active patients, unless surgically sterile, must be using a highly effective contraception method (including oral, transdermal, injectable or implanted contraceptives, IUD, using a condom of the sexual partner or sterile sexual partner) and must agree to continue using such precautions during the whole study period.<br>• Pregnant women and nursing mothers
Name of IMP	Wilzin 50mg®: 167.84 mg zinc acetate dihydrate = 50 mg Zn^{2+}<br>Wilzin 25mg®: 83.92 mg zinc acetate dihydrate = 25 mg Zn^{2+}
Investigational medicinal product – dosage and method of administration:	Zinc acetate dihydrate in age-adapted dosage ranging from 50mg to 150mg Zn^{2+} per day according to the recommended dosage in Wilson's disease.
Duration of treatment:	Treatment duration is 6 months.<br>Follow up 30 days after last treatment day.
Time plan (estimated):	First patient first visit (FPFV): Q II 2024<br>Last patient first visit (LPFV): Q II 2025<br>Last patient last visit (LPLV): Q IV 2025<br>End of trial: Q IV 2025<br>Final study report: Q I 2026
Statistician:	Dr. Petra Schiller<br>Institute of Medical Statistics und Computational Biology (IMSB)<br>University of Cologne<br>Kerpener Strasse 62<br>50937 Cologne, Germany
GCP compliance:	The present trial will be conducted in accordance with the valid versions of the trial protocol, the internationally recognised Good Clinical Practice Guidelines (ICH-GCP), the German Drug Law (Arzneimittelgesetz) and of EU Clinical Trials Regulation No. 536/2014 („CTR") including archiving of essential documents.
Financing:	The trial is financed by donations of the German parents asscociation: "GNAO1-Gemeinsam nicht allein e.V."

II. Table of contents

I. Signatures 2
II. Table of contents 6
II.a) List of tables 10
II.b) List of figures 10
III. Abbreviations 11
IV. Definitions 12
1. Introduction 14
2. Objectives of the clinical trial 18
2.1. Rationale for the clinical trial 18
2.2. Primary objective 18
2.3. Secondary and other objectives 18
2.4. Sponsor 19
2.5. Principal Investigator 19
2.6. Statistics 19
2.7. Further committees 20
2.7.1. Steering Committee 20
2.7.2. Data monitoring Committee 20
2.8. Study laboratories and other technical services 20
2.9. Central organisation units 20
2.10. Principal investigators and trial sites 21
2.10.1. Requirements for principal investigators and trial sites 21
2.11. Financing 22
3. Trial conduct 23
3.1. General aspects of trial design 23
3.1.1. Time plan 23
3.2. Discussion of trial design 24

3.3. Risk Benefit Assessment 25
3.3.1. Known Potential Risks 25
3.3.2. Known Potential Benefits 25
3.3.3. Assessment of Potential Risks and Benefits 26
3.4. Selection of trial population 26
3.4.1. Inclusion criteria 26
3.4.2. Exclusion criteria 27
3.5. Withdrawal of trial participants after trial start 27
3.5.1. Withdrawal from treatment 27
3.5.2. Withdrawal from investigations 28
3.5.3. Withdrawal from documentation 28
3.5.4. Procedures for premature treatment termination during the trial 28
3.5.5. Premature termination of trial 28
3.6. Treatment 29
3.6.1. Treatments to be given 29
3.6.2. Description of investigational medicinal product 29
3.6.2.1. Provision, storage and labelling of investigational medicinal product 30
3.6.3. Compliance with treatment / Dispensing and return of investigational medicinal product 30
3.6.4. Selection of dosage of investigational medicinal product 30
3.6.5. Adjustments to dosage of the investigational medicinal product in the individual trial subject 31
3.6.6. Previous and concomitant medication 31
3.6.7. Continuation of treatment after the end of the clinical trial 32
3.7. Efficacy and safety variables 32
3.7.1. Measurement of efficacy and safety variables 32
3.7.1.1. Primary target variable 32
3.7.1.2. Secondary and other target variables 32

3.7.1.3. Description of visits 33
3.7.2. Rationale for assessment procedures 35
3.7.3. Pharmacokinetics/Determination of drug levels 35
3.8. Data quality assurance 35
3.8.1. General aspects of quality assurance 35
3.8.2. Monitoring 35
3.8.3. Serious Breaches 36
3.8.4. Audits / Inspections 36
3.9. Documentation 37
3.9.1. Data management 37
3.9.2. Archiving 37
4. Ethical and regulatory aspects 38
4.1. Approvals by national authorities and ethics committee 38
4.2. Ethical considerations 38
4.3. Legislation and guidelines 38
4.4. Notification of the authorities and registration 38
4.5. Obtaining informed consent from trial participants 39
4.6. Insurance of trial participants 39
4.7. Data protection 39
5. Statistical methods and sample size calculation 41
5.1. Statistical and analytical plan 41
5.1.1. Analysis population 41
5.1.2. Description of trial subject groups 42
5.1.3. Primary target variable 42
5.1.3.1. Feasibility 42
5.1.3.2. Safety 42
5.1.4. Secondary and other target variables 42

5.1.5. Subgroup analyses 44
5.1.6. Interim analysis 44
5.2. Sample size calculation 44
6. Safety 46
6.1. Definitions of adverse events and adverse drug reactions 46
6.1.1. Adverse event 46
6.1.2. Adverse drug reaction 46
6.1.3. Serious adverse events and serious adverse reactions 46
6.1.4. Expected and unexpected adverse drug reaction 46
6.1.5. Suspected unexpected serious adverse reactions 47
6.2. Documentation and follow-up of adverse events 47
6.2.1. Documentation of adverse events and adverse drug reactions 47
6.2.1.1. Exceptions from AE documentation 48
6.2.2. Severity of the adverse event 48
6.2.3. Causal relationship between adverse event and investigational medicinal product 49
6.3. Reporting of serious adverse events, pregnancy and changes in risk-benefit assessment 49
6.3.1. Reports from the investigator to the sponsor 50
6.3.2. Conditions excluded from expedited reporting 50
6.3.3. Pregnancy 50
6.3.4. Obligations and Reporting Requirements of the sponsor 51
6.3.4.1. Assessment of SAEs by the sponsor 51
6.3.4.2. Notification of ethics committee and competent authority 51
6.3.4.3. Review and reporting of changes in the risk-benefit ratio 51
6.3.4.4. Informing the investigators 51
6.4. Annual safety report (DSUR) 52
7. Trial results and publication 53

7.1. Reports 53

7.1.1. Final report 53

7.2. Publication 53

8. Literature 54

9. Appendices 55

9.1. Steering Committee 55

9.2. References 55

9.3. Authors of this Trial Protocol Template: G Grass; University of Cologne (Sponsor); CTC Cologne 58

II.a) List of tables

Table 1: Definitions 12

Table 2: Time plan of the trial 23

Table 3: Scheduled dosage of the investigational medical product 30

Table 4: Visit schedule 34

II.b) List of figures

Figure 1: Trial scheme 24

Figure 2: Sample size calculation chart 45

III. Abbreviations

abbreviation	meaning
AE	Adverse Event
AIMS	Abnormal Involuntary Movement Scale
ALP	Alkaline phosphatase
ASD	Anti-seizure Drug
BfArM	Federal Institute for Drugs and Medical Devices (Bundesinstitut für Arzneimittel und Medizinprodukte)
BFMDRS	Burke-Fahn-Marsden Dystonia Rating Scale
CA	Competent authority (BfArM, PEI)
CRF	Case Report Form
DBS	Deep Brain Stimulation
DMC	Data Monitoring Committee
DSUR	Development Safety Update Report
EC	Ethics Committee
GMFM-66	Gross Motor Function Measure
GOT	Glutamic-oxaloacetic transaminase
GPT	Glutamate pyruvate transaminase
ISF	Investigator Site File
IMP	Investigational Medicinal Product
MD	Movement disorder
PI	Principal Investigator
PEI	Paul-Ehrlich-Institut
SAE	Serious Adverse Event
SUSAR	Suspected Unexpected Serious Adverse Reaction
TMF	Trial Master File

IV. Definitions

Table 1: Definitions

The following definitions apply with respect to the trial population

Term	Definition
completer	Subject who underwent the clinical trial in accordance with the clinical study protocol.
date EoS<br>(End of Study)	**For Completer:** Date of completion of all protocol-required elements in a trial by an enrolled subject.<br>**For Drop-out:** Date of last documented, protocol-required visit/assessment with the subject.
drop-out<br>(early dis-continuation)	Drop-out is the subject who has signed an IC, was eligible, enrolled, with at least one IMP intake or treatment and discontinued the clinical trial for any reason before the end as defined in the trial protocol.<br>NOTE: Reasons for drop-out / discontinuation are defined:<br>a) withdrawal: active discontinuation by a subject;<br>b) investigator initiated discontinuation (e.g., for cause);<br>c) lost to follow-up: cessation of participation without notice or action by the subject;<br>d) sponsor initiated discontinuation.<br>Assignment to analysis population depends on the category and time of the discontinuation.
enrolment	The process of registering or entering a patient into a clinical trial. Once a subject has signed IC, undergone all screening assessments and is eligible according to inclusion/exclusion criteria, he/she can be enrolled.
inclusion/<br>exclusion<br>criteria	The medical or other guidelines that determines whether a person may or may not be allowed to enter a clinical trial. These criteria are based on such factors as age, gender, the type and stage of a disease, previous treatment history, and other medical conditions. Also known as Eligibility or Enrolment Criteria.
lost to follow up	Subcategory of a drop-out:<br>Enrolled subjects with unknown treatment/ observation outcome e.g. due to unreported deaths, unknown transfer of care w/o documentation, disengagement from care
recruitment	Subjects are selected on the basis of the protocol's inclusion and exclusion criteria during the clinical trial recruitment period.<br>The number of subjects that must be recruited for enrolment into a trial and meet the requirements of the protocol.

subject/ participant	An individual who participates in a clinical trial either as a recipient of the investigational product(s) or as a control.<br>The term "subject" is part of the federal regulation and may be used interchangeably with participant.
termination	Discontinuance, by sponsor or by withdrawal of IRB / FDA approval, of a clinical trial before completion. This termination can be at a site or the entire trial.
Withdrawal	Subcategory of a drop-out:<br>The subject-initiated act of discontinuing participation in a clinical trial.<br>NOTE: Withdrawal can range from the subject's complete withdrawal from trial procedures and follow-up activities, to the subject’s withdrawal from trial-related interventions while the subject permits continued access to his / her medical records or identifiable information.<br>NOTE: according to FDA regulations (and AMG - Ger), when a subject withdraws from a trial, the data collected on the subject to the point of withdrawal remain part of the trial database and may not be removed.

1. Introduction

Different variants in GNAO1 have been reported in children with an early onset global developmental disorder over the past ten years.(*1-3*) There is a wide spectrum of different symptoms in GNAO1 related disorders that can occur and vary in severity and type of manifestation. This heterogeneity is based on the different variants within GNAO1. The core clinical features of this disease are a global developmental delay, a complex hyperkinetic movement disorder (MD) alerting with central muscular hypotonia with or without epilepsy. The disease manifest early especially when seizures occur. In the German GNAO1 registry more than the half of the patients manifested in the first four weeks of life with seizures or/and muscular hypotonia and nearly all patients showed symptoms within the first year of life.(*4*) The MD is characterised by dystonia, choreoathetosis, and ballism.(*5*) External triggers like infection, positive or negative emotions and change of positioning may aggravate the clinical features temporally.(*6*) Patients that manifest with seizures often develop early infantile epileptic encephalopathy (EIEE) with pharmaco-refractory seizures of various semiology. In other patients, seizures occur either late or not at all and the MD is the predominant phenotype. Some patients suffer of hyperkinetic crisis (status dystonicus) in the course, which is a life-threatening MD emergency defined as a temporary period with increased and nearly permanent involuntary movements or permanent dystonic posturing, requiring hospital admission, monitoring on an intensive care unit and a massive escalation of pharmacotherapy.(*7*) Lately there are more reports of milder phenotypes in GNAO1(*8*) suggesting that there might be an underreporting of this disease. Nevertheless, most of the patients known to date are severely affected with mental retardation and high mortality in childhood.

Heterotrimeric G proteins are the immediate cytoplasmic signaling transducers of G protein-coupled receptors (GPCRs) – the largest receptor class in animals and the major target of modern drugs (*9*). Composed of the Gα, β, and γ subunits, they interact with receptors when the Gα is loaded with GDP to undergo the activated GPCR-induced GDP-to-GTP exchange and dissociation into the Gα-GTP and Gβγ components, both competent to transmit the signal further downstream (*10*). With time, intrinsic GTPase activity of the Gα-subunits leads to GTP hydrolysis – the activity further sped up by the dedicated RGS (Regulator of G protein Signaling) proteins (*11*). The resultant Gα-GDP can reload with GTP (*12*) or complex back with Gβγ, thus closing the G protein activation-deactivation loop (*10*).

Of the 16 human Gα-subunits, Gαo is the major neuronal representative, transmitting the signals from numerous GPCRs in developing and adult brain. In 2013, the first cases were reported on pediatric encephalopathy patients harboring de novo mutations in GNAO1 – the

gene encoding Gαo (*13*). This discovery was followed by an avalanche of clinical analyses that cumulatively led to the clinical presentation as described above. Although Gαo, in addition to neurons, is also strongly expressed in glial cells (www.proteinatlas.org), disease modeling in mice (*14*) and brain organoids (*15*) demonstrated strong effects of GNAO1 mutations on neuronal rather than glial differentiation, highlighting neurons as the main target of Gαo malfunctioning in the disease. Gαo couples to many neuronal GPCRs, such as D2-dopamine, µ-opioid, M2-muscarinic, α2-adrenergic, and more. As these GPCRs belong to the inhibitory receptors, Gαo misfunctioning induced by mutations may be expected to disbalance the equilibrium formed by the stimulatory and inhibitory GPCR signaling and thus contribute to the disease manifestations (*16*). Another major function is the regulation of cyclic adenosine monophosphate (cAMP) production by the influence on the availability of Gβγ for Adenyl cyclase type 5 (AC5).(*17*) As second messenger, cAMP is significantly involved in cellular signal transduction and the balance of the neuronal system.(*18*) AC5 is an enzyme that catalyzes the formation of cAMP from ATP with elimination of pyrophosphate. A reduced level of cAMP due to ADCY5 mutations has been identified as a cause of MD characterized by involuntary hyperkinesia.(*19, 20*) This link between lower cAMP level and MD, could also explain some of the MD symptoms of children with GNAO1 mutations and therefore reduced cAMP level.

No curative therapy exists for GNAO1 patients until now. Symptomatic treatments for the motor dysfunctions include pharmacotherapy and deep-brain stimulation, while for the epilepsy – different antiepileptics, but at best with partial effects (*21-23*). Until now only animal models for GNAO1-specific therapy exists. The fruit fly Drosophila melanogaster represents an excellent model organism for studies in various fields of biology (*24*), and the recent work of Savitsky et al. highlights the power of Drosophila as the host to model GNAO1 encephalopathy (*25*).

To establish a GNAO1-encephalopathy model in the fruit fly, our cooperation partner in this study Vladimir Katanaev (*26*) and his working group applied the CRISPR/Cas9-mutagenesis together with phiC31-mediated recombinase-mediated cassette exchange (*27*) to introduce the pathogenic G203R mutation into the Drosophila Gαo. They could show that [G203R]/+ flies are viable and fertile yet reveal a number of deficiencies. Specifically, the heterozygous mutant flies manifest a significant motor dysfunction, measured in the negative geotaxis assay as a reduced capacity to climb up the wall, reminiscent of the motor dysfunction in the human patients. Further, the encephalopathy mutant Drosophila display a two-fold reduction in the life span. Regional brain atrophy, sometimes progressive, has been described in GNAO1 patients with the G203R mutation and may be the outcome of epileptic onsets (*28-31*). Analysis of 35 days-old [G203R]/+ flies revealed limited yet significant brain degeneration as well. Given the neonatal lethality of GNAO1[G203R]/+ mice (*14*), they thus established the first viable animal

model of G203R encephalopathy with this Drosophila line, capable of recapitulating some of the clinical manifestations of the disease.

They could show that $ZnCl_2$-containing food significantly improves the motor function of the G203R/+ flies. The effect of $ZnCl_2$ was particularly strong for female Drosophila, bringing the climbing capacity towards the wild-type levels. $ZnCl_2$ food supplementation also rescued the reduced life span of female G203R/+ flies. Overall, these observations reveal strong improvements of the behavioral and life span conditions in the Drosophila model of GNAO1 encephalopathy by dietary zinc supplementation.

To understand this finding they showed that some mutations in GNAO1 accelerate GTP uptake and inactivate GTP hydrolysis through displacement Gln205 critical for GTP hydrolysis resulting in constitutive GTP binding by Gαo. However, the mutants fail to adopt the activated conformation and display aberrant interactions with signaling partners. The structural modeling and molecular dynamics analysis suggests the atomistic mechanism for the action of Zn^{2+} on the restoration of the GTPase activity as the bringing back of the catalytic Gln205 to the γ-phosphate of GTP, otherwise swayed away by the mutations.

Zinc supplementation has been applied to treat various human health conditions, including the neurological ones. In Wilson’s disease, high dosages of zinc are administered over a long, sometimes lifelong period with only rare and minor side effects.(*37*). Additionally zinc has been approved for treatment of various neurological conditions,(34, 38) such as depression (32), epilepsy (33), psychiatric and sleep disorders (35), as well as to support normal neonatal development (36). The daily dose of elemental zinc in Wilson’s disease treatment is 50mg (corresponding to 168mg zinc acetate dihydrate) daily for children under 6 years of age.(39) We suggest that a similar dose should be applied for the treatment of GNAO1 patients older than 6 months. In Wilson’s disease most trials included patients older than one year, since this disease is often diagnosed at an older age. GNAO1 associated disorders manifests early in life and in more than 90% in the first year of life. (40) Most patients show severe symptoms in the first year of life including developmental delay, intellectual disability, movement disorder and epilepsy. If zinc is effective, it should be applied as early as possible to prevent sequalae and to achieve the greatest treatment success. We therefore propose that young children under the age of 1 year should be included into this trial using the same dosage as children above 1 year to prove safety and feasibility in this trial. Prior this trial, we aimed at assessing the safety profile of zinc in animal models of the disease to ensure safety for this trial. In the Drosophila model based on the GNAO1[G203R] allele, we supplied $ZnCl_2$ to the final concentration of 200μM into the food, following the prior study has been previously shown to rescue the survival of Drosophila mutant for dZip1 and dZip2, the gut zinc transporters.(41)

This supplementation resulted in a significant rescue of the motor dysfunction and reduction in the half-life in the Drosophila model of GNAO1 encephalopathy, without any noticeable adverse effects.(42) In this study the zinc containing food was given already to the larvae of the Drosophila. This supports the argumentation that zinc should be applied as early as possible.

In mice, zinc toxicity has not been evaluated in neonates and young pups, whereas zinc salt supplementation has been well-studied in adult animals. For example, maintenance of adult mice for up to 14 months on water supplemented with 0.5g/L elemental zinc in the form of $ZnSO_4$ resulted in no adverse effects in the animals;(43) the maximum tolerated dose (MTD) for adult mice is reported as 75mM (12.11g/L) $ZnSO_4$ in drinking water.(44) We thus first tested the near-MTD doses of $ZnSO_4$ supplied in drinking water to C57BL/6 mice all the way from birth to adulthood: 4-8g/L (ca. 1000-2000mg/kg/day of $ZnSO_4$; the human equivalent dose can be estimated (following fda.gov/media/72309/download) as 81-162mg/kg/day). By the end of the 3-month measurement period, no differences were observed in the appearance of animals treated with the highest dose of $ZnSO_4$. Finally, after 3 months of this treatment, the animals were sacrificed and the weight and appearance of major organs were evaluated at necropsy No difference in organ weight or appearance was observed. Taken together, these results indicate the safety of the near-MTD doses of Zn^{2+} their suitability for long-term treatment.(45)

In the C215Y mouse model of GNAO1 encephalopathy,(14) continuous supplementation of $ZnSO_4$ (2g/L in the drinking water) leads to no behavioral disturbances. Instead, noticeable improvement in the mouse performance in the rotarod test and in the novel object recognition test was observed in the tested mice regardless of their genetic background. From these findings showing the safety of high-dose zinc treatment in mice, (45) we hypothesize that the dosages applied in this trial will be safe also for young patients.

With this background, we applied zinc supplementation therapy to a 3.4 years-old patient with the c607G>A, p.Gly203Arg variant with oral 50mg zinc (in the form of zinc acetate) daily, as applied in Wilson’s disease. During 11 months of treatment, the patient showed a clinical improvement and an excellent safety profile.(45)

2. Objectives of the clinical trial

2.1. Rationale for the clinical trial

Children and young adults affected by GNAO1 associated disorders usually show a very severe phenotype with high level of morbidity and early disease onset. Until now there is no disease specific therapy. In contrast, there is a clear understanding of the underlying mechanism of Gαo in the brain and in the presented animal model with fruit flies in which the application of zinc lead to a significant improvement of symptoms. Zinc is easily oral applicable and is supplemented in various diseases. Thus, there is a broad knowledge of treatment safety and the risk for the participants is therefore minimal.

2.2. Primary objective

- Feasibility of treatment with oral zinc demonstrated by taking the study medication in 80% of the days in the trial
- Safety of daily administered zinc in GNAO1 by regular evaluation of the AEs as defined in chapter 6.1.

2.3. Secondary and other objectives

- Level of Motor-Skills measured by Change of Gross-motor function measure (GMFM-66)
- Quality of life measured by CP-Child Questionnaire for the caregivers and Canadian occupational performance measure (COPM)
- Level of Dystonia measured by Change of Burke-Fahn-Marsden Dystonia Rating scale (BFMDRS),
- Level of dyskinesia measured by Abnormal involuntary movement scale (AIMS) and a Movement log of the parents
- Changes in general behavior including level of alertness, better sleep
- Changes in Seizure logs (times, duration, frequency)
- Changes of Motor Skills and level of dyskinesia in correlation to patient specific variant in GNAO1

- Serum controls of zinc to measure efficacy of oral zinc administration
- Serum ferritin and copper detect potential deficiencies, caused by regular zinc administration and therefore reduced uptake of iron and copper
- Analyze of the microbiome in stool

Organisational and administrative aspects of the trial

2.4. Sponsor

Sponsor:	University of Cologne
Represented by:	Dr. Moritz Thiel<br>Children’s Hospital, University Hospital Cologne<br>Kerpener Strasse 62<br>50937 Cologne<br>Germany

2.5. Principal Investigator

Principal Investigator (PI):	Dr. Moritz Thiel<br>Children’ Hospital, University Hospital Cologne<br>Kerpener Strasse 62<br>50937 Cologne, Germany

2.6. Statistics

Statistician:	Dr. Petra Schiller Institute of Medical Statistics and Computational Biology (IMSB)<br>University of Cologne<br>Kerpener Strasse 62<br>50937 Cologne, Germany

2.7. Further committees

2.7.1. Steering Committee

The steering committee is involved in the planning and set up of this trial. Their tasks consist to critical monitor recruitment and safety and discuss and interpret the results. A list of the members of the Steering Committee is given in Appendix 9.1.

2.7.2. Data monitoring Committee

A data monitoring Committee has been established consisting of two clinicians and one statistician which is going to monitor the trial in regular meetings focussing on the safety aspects.

2.8. Study laboratories and other technical services

The functional analysis of the mutations as well as the analysis of the microbiome in stool will be performed by the Laboratory in Geneve.

- Translational Research Center in Oncohaematology,
  Department of Cell Physiology and Metabolism,
  University of Geneva,
  CMU Rue Michel-Servet 1
  1211 Genève, Switzerland

The blood tests including zinc will be performed by the clinical inhouse laboratory of clinical chemistry.

Institut für Klinische Chemie
Director: Prof. Dr. Thomas Streichert
University Hospital Cologne
Kerpener Strasse 62
50937 Cologne, Germany

2.9. Central organisation units

Principal investigator:

Dr. Moritz Thiel
Children's Hospital, University Hospital Cologne

University of Cologne
Kerpener Strasse 62
50924 Cologne, Germany
Tel. +49 221-478-40652

Clinical project management and administrative support:

Klinisches Studienzentrum Pädiatrie
/ Pediatric Clinical Trials Center
Head: Dr. Barbara Hero
Children’s Hospital, University Hospital Cologne
University of Cologne
Kerpener Strasse 62
50924 Cologne, Germany
Tel. +49 221-478-6850/6853

Investigator and clinical support:

PD Dr. Kyriakos Martakis
Children’s Hospital, University Hospital Cologne
University of Cologne
Kerpener Strasse 62
50924 Cologne, Germany

Monitoring, Data management and Safety management:

Clinical Trials Centre Cologne (CTCC)
Gleueler Strasse 269
50935 Cologne, Germany

2.10. Principal investigators and trial sites

This clinical trial will be open as monocentre study at the Children’s Hospital of Cologne.

2.10.1. Requirements for principal investigators and trial sites

Proof of knowledge of regulatory procedures. Personnel and spatial capacities to carry out the examinations at the specified intervals. Previous survey experience with GMFM-66 and BFMDRS.

2.11. Financing

The clinical trial is financed by the German patient association: “GNAO1-Gemeinsam nicht allein e.V.”.

Additional own resources at the Children’s University Hospital Cologne are used.

3. Trial conduct

3.1. General aspects of trial design

This pilot study is a single-arm, open-label pilot trial for participants with GNAO1 associated disorders. All participants will receive the study medication zinc orally in age dependent doses twice or three times a day for 6 months. (see *Table 3* page 309)

3.1.1. Time plan

Table 2: Time plan of the trial

First patient first visit (FPFV):	Q II 2024
Last patient first visit (LPFV):	Q II2025
Last patient last visit (LPLV):	Q IV 2025
End of trial:	Q IV 2025
Final study report:	Q I 2026

Individual Process for each patient within the trial

After informed consent, the investigations scheduled at baseline for inclusion into the trial take place (see figure below and *Visit schedule Table 4*, Page 34). The first blood sample may be taken as part of a routine blood test (which is scheduled every 6 months in dystonia and epilepsy patients) and the value of zinc, copper and ferritin in serum is additional determined. As this is a single-arm trial, all participants will receive the trial medication. Starting after baseline, Zinc is taken twice or three times a day in an age-dependent dose (see *Table 3* page 29). 10 ±7 days post inclusion a phone call is made to ask for adverse effects. After 3 months (90 days ± 10 days), visit 2 (T2) takes place containing a thorough clinical examination, evaluating the clinical scores and blood samples. After 6 months (180 days ±10 days), visit 3 (End of treatment visit) is scheduled. Again a thorough clinical examination is carried out and the clinical scores are evaluated. The serum levels of zinc copper and ferritin are checked as well as safety lab blood tests. If a clinical benefit of the treatment is observed, the parents may continue to administer the medication (outside the trial). If not they will stop the medication at the End of treatment visit (EOT, T2). 30 +10 days after the last follow up a phone call ensures that no further adverse events occurred in patients that stopped the medication.

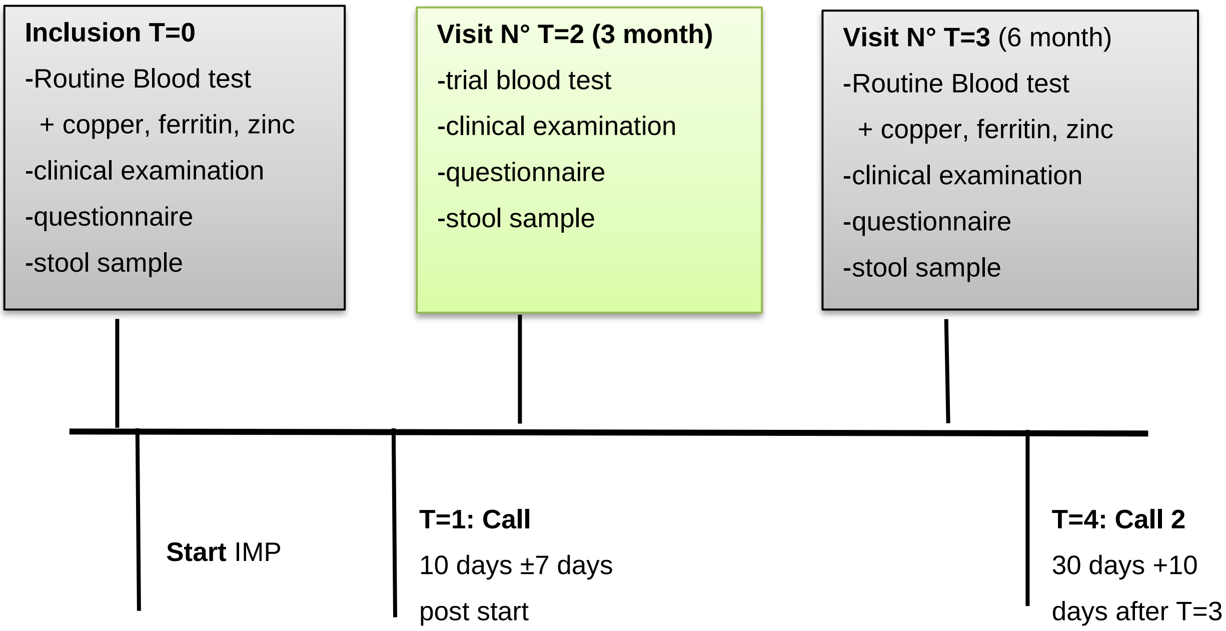

* Trial Blood Test: Complete blood count, liver and kidney values, zinc, copper and ferritin

Figure 1: Trial scheme

End of the clinical trial

The end of trial is defined by 30days + 10day (phone call T4) after the last trial medication of the last patient treated within the trial.

3.2. Discussion of trial design

In this trial oral daily zinc in an age adapted dosage is administered in participants with a GNAO1 associated disorder as described in the introduction. Due to pilot character of this trial in this ultra-rare disorder only a small number of twelve participants will be included. Therefore, we assume that the open label design is the most promising way to a) elaborate if the daily therapy with zinc is feasible and safe, b) if there are any clinical effects from this medication and c) if the chosen clinical tests are valid to measure the clinical effect. The clinical assessments (GMFM-66, BFMDRS; AIMS) used in this trial are regularly used in the clinical routine and are therefore valid. Nevertheless, the questionnaire CP Child, which is completed by caregivers, could be influenced by the placebo effect and the wish of the caregivers to see positive effects of the IMP. The questionnaire should therefore always be interpreted in the context of objective clinical testing and checked for consistency.

3.3. Risk Benefit Assessment

The overall risk of this trial for the participants is low. Zinc supplementation is broadly used and the administered dosage does not exceed the dosages used in Wilson's disease. In Wilson's disease the dosage is approved also in long term intake as proven in many publications. (*46-54*) So far there is no clue that GNAO1 patients experience more or different side effects than the general population. For patients younger than 12 months there are no trials in Wilsons's disease. Therefore, the dosage recommendations in Wilson's disease are above one year. We want to include patients aged 6 months and older in this trial. There are multiple trials in which zinc is applied to neonates and in a Cochrane review for enteral zinc supplementation for prevention of morbidity and mortality in preterm neonates, zinc proved to be safe even in preterm.(*55*) The applied dosages in this trial are far below the toxic limit of zinc and chosen with the aim of the greatest possible safety for patients. Nevertheless, based on the preliminary in animal models and in vitro tests, we assume that we have to dose higher than the substitution dose of zinc.

3.3.1. Known Potential Risks

The well-known but controllable risks of taking zinc are acute side effects such as nausea, abdominal pain and vomiting right after intake. In Wilson's disease around 20% of the patients experience such side effects in the first two weeks of therapy. In most of the patients this side effect disappears after this initial period. In the others this side effect is can be improved by administering the drug to a stomach that is not entirely empty. In the long-term supplementation zinc can lead to reduced iron absorption. This could eventually lead to anaemia. It can also lead to a reduced absorption of copper. It is described that when taking zinc there might be a temporary increase in some liver enzymes (alkaline phosphatase, amylase and lipase), but this does not cause any illness. If zinc is taken for a longer period or is discontinued, these values normalize. Due to the fact that zinc is a licensed product, a summary of product characteristics (SMPC) is available and will be filled in the Trial Master File (TMF)/ Investigator Site File (ISF).

3.3.2. Known Potential Benefits

If the therapy with zinc shows comparable treatment effects as in the animal model, this would be an enormous benefit for the participants. So far, there is no specific therapy for this disease and zinc would be the first therapy with few side effects that could potentially reduce the degree of disability and improve motor skills in GNAO1.

3.3.3. Assessment of Potential Risks and Benefits

Nausea, abdominal pain and vomiting is often a temporary problem. If it is prolonged, it can be improved by administering the drug to a stomach that is not entirely empty or at a different daytime. All participants are called 10±7days after starting the IMP as described above to ask for these side effects and to adjust the medication if necessary. To counteract the risk of lower copper and ferritin absorption, the serum levels of copper and ferritin are determined regularly. If the value falls, either ferritin or copper can be substituted. The possible benefits will be assessed by the trial investigations (e.g. BFMDRS).

3.4. Selection of trial population

Patients with pathogenic GNAO1 variants manifest mostly in the first year of life (*4*) and are often mentally and physically handicapped. Therefore, it is unavoidable to include participants who are not able to give informed consent on their own. Parents or legal guardian must give informed consent in order to ensure the welfare of the patients aged younger than 18 years, but the consent of the patient is needed in addition, as far as the patient is in a state of psycho-intellectual development to understand the information provided. Should participants be 18 years and older and are legally patronized due to their GNAO1-associated disorder, the legal guardian decides on participation in the study.

3.4.1. Inclusion criteria

- GNAO1 associated neurological disorder, documented by either:

  Proven pathogenic or likely pathogenic mutation in GNAO1 or a variant of unknown significance in GNAO1 and clinical symptoms likely to be consistent with GNAO1 as determined by the investigators

  and

  at least one of the common symptoms of GNAO1: Movement disorder (dystonia, chorea, ataxia, stereotypic movements, clonic), central muscular hypotonia, epilepsy, global developmental delay
- Age: 6month-30years
- GMFM ≤ 75
- Written informed consent prior to any trial-related procedure by parents or legal guardian

(due to severe cognitive impairment, informed consent by patients is not feasible even with older age or in adulthood)

- Stable on following concomitant treatments for at least 3 months prior to trial inclusion: anti-seizure medication (ASD); baclofen, Deep brain stimulation settings

3.4.2. Exclusion criteria

- Treatment of Zinc in the last 4 months before inclusion
- Known other genetic variants that are known to cause symptoms like observed in GNAO1-related disorders, additional to the proven GNAO1 mutation
- Implantation of Deep brain stimulation planned during the duration of the trial, i.e. in the six months after inclusion
- Start of intrathecal baclofen therapy planned during the duration of the trial, i.e. in the six months after inclusion
- Known allergy/hypersensitivity to the scheduled trial drug
- Concomitant participation in other clinical drugs with investigational drugs or with competing interventions
- Sexually active participants who are not willing to use/ not using a highly effective contraception method with a pearl-index < 1. Sexually active participants resp. their partner, unless surgically sterile, must be using a highly effective contraception method (including oral, transdermal, injectable or implanted contraceptives, IUD, using a condom of the sexual partner or sterile sexual partner) and must agree to continue using such precautions during the whole study period.
- Pregnant women and nursing mothers

3.5. Withdrawal of trial participants after trial start

3.5.1. Withdrawal from treatment

Withdrawal from treatment is indicated

- at any time, if consent is withdrawn (by patient, parents or legal guardian)

- if a clinical adverse event (AE), laboratory abnormality, illness, or other medical condition or situation occurs such that – according to the judgement of the treating physician – continued participation in the study would not be in the best interest of the subject.

3.5.2. Withdrawal from investigations

The investigational procedures will not be performed

- at any time, if the patient, parents or legal guardian withdraw consent from participating in the study or investigation.
- if, due to medical condition or situation, the procedures would not be in the best interest of the patient according to the judgment of the treating physician

3.5.3. Withdrawal from documentation

At any time, if patient parents or legal guardian withdraw consent from study documentation. Data collected until the withdrawal of consent will be further stored and used according to the trial protocol. If treatment is discontinued or trial investigations are not performed due to medical reasons as defined above, patient’s data will be documented as far as consent to documentation is still given.

3.5.4. Procedures for premature treatment termination during the trial

If treatment is discontinued, a complete final evaluation according to visit 3 EOT (table 3) following the patient's withdrawal should be made. AEs will be followed by a phone call 30 (+ 10) days after premature termination.

3.5.5. Premature termination of trial

The sponsor has the right to terminate the trial prematurely if there are any relevant medical or ethical concerns, or if completing the trial is no longer practicable. If such action is taken, the reasons for terminating the trial must be documented in detail. All trial participants still under treatment at the time of termination shall undergo a final examination which must be documented. The sponsor must be informed without delay if any investigator has ethical concerns about continuation of the trial.

Premature termination of the trial will be considered if:

- The risk-benefit balance for the trial subject changes markedly

- It is no longer ethical to continue treatment with the IMP
- The sponsor considers that the trial must be discontinued for safety reasons
- It is no longer practicable to complete the trial

If premature termination of the trial is considered, the principal investigator decides on whether to discontinue the trial in consultation with the steering committee.

3.6. Treatment

3.6.1. Treatments to be given

In this single-arm trial, all participants will receive the trial drug zinc acetate dihydrate orally. The IMP will be given one hour after meal in a dosage (as listed in Table 3) which is recommended in Wilson's disease and has been given in this condition without observing severe adverse effects. If oral administration is not possible due to the disability level of the patient, the IMP can be mortared and suspended and can then be given as suspension orally or via the PEG. The total treatment duration in each patient is 6 months with stable dosage over the duration of the trial. If the therapy shows effects, the parents and participants may continue medication after the end of the trial. If not, they will stop the medication after the last visit at the trial site (T3). 30 +10 days after the last visit, a phone call ensures that no further adverse events occurred.

3.6.2. Description of investigational medicinal product

There are different formulations of zinc supplements available on the market. In this study, zinc acetate dihydrate (Wilzin 50mg® or Wilzin 25mg®) will be used. The dosage in *Table 3* refers to the zinc ion (Zn2+) concentration.

The IMP has already been approved for use in humans for the following indication: Supplementation of zinc deficiencies, therapy with penicillamine and other chelating agents, therapy of Wilson's disease, acrodermatitis enteropathica. The only contraindication is an hypersensitivity to zinc in the supplemented form. In addition, zinc is freely available in many drugstores and is taken to strengthen the immune system.

3.6.2.1. Provision, storage and labelling of investigational medicinal product

The study drug will be provided by the Sponsor, will be labelled according to CTR Annex 6 and will be handed over to the participants with drug accountability. The trial medication should be stored at room temperature

3.6.3. Compliance with treatment / Dispensing and return of investigational medicinal product

The supply for the first three months of treatment will be given to the trial subject at the first visit (T0). At Visit T2, 3 months later, the supply for the following 3 months will be given to the subject. Unused IMP will be returned to the site at visit T3 and destroyed at site afterwards. To improve treatment adherence and to ask for early adverse effects a phone call is made 10 ±7 days after the start of the therapy. There is a diary that will be handed to the trial subject at T0 in which the daily intake of the IMP has to be noted. At every visit the participants diary will be reviewed to detect possible breaks of intake.

3.6.4. Selection of dosage of investigational medicinal product

The dosage is defined by the age resp. body weight at study entry (T0) as given in Table 3 and will not be changed over the duration of the trial. The total daily dosage will be divided in two or three doses as proposed in Table 3.

Table 3: Scheduled dosage of the investigational medical product

Age or weight	Daily dosage of Zinc ion Zn2+ in mg/day	Number of doses /day	Mg/dose in this trial morning-noon-evening
**6month-<6 years**	50	2	25-0-25
**6-<16 years**	75	3	25-25-25
**≥16 years or >57kg**	150	3	50-50-50

For detailed rationale see Introduction. In this trial the dosages for patients older than 1 year are equal to the dosages administered in Wilson's disease. Since GNAO1 associated disorders manifest in the first year of life we argue that zinc should be administered early in life and therefore already investigated in children older than 6 months to prove safety and feasibility. The proposed dosages of zinc are chosen as high as the dosages used for substitution of zinc, since high levels are needed to pass through the Blood-Brain-Barrier and pre-clinical models give rationale for a supraphysiological substitution.

3.6.5. Adjustments to dosage of the investigational medicinal product in the individual trial subject

The most expected side effects are gastrointestinal problems. They can mostly be treated with changing the intake time after a small meal. It’s known that the gastrointestinal symptoms decrease without any adjustment of dosage over time. The other expected side effect is a reduced iron or copper intake resulting in reduced serum levels of these. In the long term reduced supply of iron could lead to reduced production of haemoglobin resulting in anaemia, reduced levels of copper may lead to paraesthesia and weakness. In most cases this side effect can be treated with a substitution therapy with iron or copper.

If the substitution of zinc is planned beyond the trial and the substitution of iron or copper over 12 weeks in recommended dosage did not treat the low level of copper or ferritin, reduction of zinc should be taken into account by the treating physician. (e.g. halved for another 12 weeks, then further reduction to a minimum of 25mg Zn^{2+}/day)

3.6.6. Previous and concomitant medication

Patients with GNAO1 are often polymedicated with various drugs that treat both movement disorders and epileptic seizures. For this trial, any concomitant medication deemed necessary by the investigator is allowed, however it is required that drugs such as tetrabenazine, trihexyphenidyl and gabapentin, often given for long-term treatment of the movement disorder and in particular of dystonia, have already been used in stable doses for at least 3 months prior to study entry. The same applies to the ASD. Sedatives such as clonazepam or chloral hydrate are often given as needed with varying frequency on a daily or weekly basis, therefore stable doses do not have to be achieved. The following interval has to be considered between inclusion and therapeutic measures. Deep brain stimulation must have been implanted at least 6 months prior to the start of the trial and should have been stable for at least 3 months prior to study entry. A baclofen pump should also have been implanted at least 6 months before the start of the trial and the rate of intrathecal baclofen should have been stable for at least 3 months prior to study entry.

The use and dosage of above mentioned concomitant medication will be documented including for cause medications for disease related symptoms. Relevant previous medication and therapeutic interventions will be documented as well.

3.6.7. Continuation of treatment after the end of the clinical trial

Participants with clinical benefit to the trial medication may continue with Zinc treatment after the end of the scheduled trial treatment at the discretion of the treating physician. If the patient / family wants to stop the medication, the last dose is taken on the day of the T3/ EOT visit after six months. If the medication is further administered, we recommend determination of serum levels of copper and ferritin every 6 month to detect possible deficiencies caused by zinc intake as described in 3.6.5.

3.7. Efficacy and safety variables

3.7.1. Measurement of efficacy and safety variables

3.7.1.1. Primary target variable

The primary objectives of the trial are safety and feasibility of zinc, thus the primary target variables are as follows:

- The feasibility is measured by the actual days that zinc was taken in the right dosage. If the IMP was taken in the scheduled dosage at least on 80% of the days it is assumed to be feasible.
- The safety is measured by regular evaluation of the AEs as defined in chapter 6.1.

3.7.1.2. Secondary and other target variables

- Level of Motor-Skills
    - measured by the Gross-Motor-Function messure-66 (GMFM-66) at visit 0, 2 and 3.

      In this standardized test the subject has to show different tasks to measure the level ranging from 0-100% of gross-motor-function. The GMFM-66 was initially created to evaluate the motor function level in patients with cerebral palsy. Over time, it became apparent that this measure is also well suited for other diseases. It is therefore used today for all diseases that are associated with a measurable delay in motor development.
- Quality of life
    - Measured by the Caregiver Priorities & Child Health Index of Life with Disabilities (CP-Child) Questionnaire in Visit 0,2 and 3

  - Measured by the Canadian Occupational Performance Measure (COPM) in Visit 0 and 3

- The level of dystonia is measured by the Burke-Fahn-Marsden Dystonia Rating scale (BFMDRS) and the level of dyskinesia by the Abnormal involuntary movement scale (AIMS) at visit 0, 2 and 3.
  For the BFMDRS a video has to be recorded and the rating scale is calculated blinded based on the video footage by an experienced paediatric neurologist. In addition, the Movement log for the parents can give an overview in which percentage of the awake daytime involuntary movements, dystonia or choreoathetosis occur.
- General behaviour including level of alertness and sleep is recorded by the parents in a daily logbook that has to been filled out during the trial.
- Frequency, time and duration of seizures is also recorded by the parents in a logbook.
- Serum levels of zinc at visit 0, 2, 3 to measure the efficacy of oral zinc administration.
- Serum level of ferritin and copper at visit 0, 2, 3 to detect potential deficiencies, caused by reduced uptake because of the regular zinc administration.
- Analyzation of the microbiome in the stool by the collaborating laboratory to detect any changes respect to the GNAO1 affected cells of the intestinal.

As far as feasible, depending on patient numbers, the individual results will be put in correlation to the specific variant of the patient to see if the outcome is influenced by the specific variant.

3.7.1.3. Description of visits

Patient visits are carried out at the following timepoints (see Table 4). Visit 1 and 4 are phone calls to the parents and do not require a presence at the site. Visit 0, 2 and 3 require presence in the hospital. The stool samples should be collected at home in the three days before visit 2 and 3 in special sample tubes that are handed out to the patient at visit 0. The first stool samples should be collected at visit 0 or on the first three days thereafter. All stool samples should be stored in the fridge at home until the next visit. The stool samples should be brought to the visit in a cooler with ice or cool packs to prevent thawing. In the clinic the stool samples will be labeled and stored in -80°C freezer and will be sent to the laboratory

in overnight transport on dry ice. At visit 0, 2 and 3 a clinical examination is carried out and clinical scores are evaluated.

Table 4: Visit schedule

**Visit N°**	**0**	**1**	**2**	**3 (EOT)**	**4**
**Trial day**	Day 0	Day 10 (+/-7)	Day 90 (+/- 10)	Day 180 (+/- 10)	Day 210 (+ 10)
Informed consent	x				
Inclusion/exclusion criteria	x				
Medical history, demographic data	x				
Clinical examination[1]	x		x	x	
BFMDRS	x		x	x	
GMFM-66	x		x	x	
AIMS	x		x	x	
COPM	x			x	
CP-Child	x		x	x	
Stool samples	x		x	x	
Serum level of zinc, copper, ferritin	x*		x	x*	
Pregnancy test in sexually active participants	x		x	x	
Safety assessment: Incl. Blood count; Na^{+}; Ca^{2+}; Mg^{2+}; K^{+}; GOT; GPT; ALP; Lipase; Amylase Creatinine, CRP	x		x	x	
Phone call		x			x
Information from diary		x	x	x	

* possible in addition to routine blood sample

1. the clinical examination includes a physical examination of all organ systems, paediatric neurology status assessment and the recording of vital signs,

Duration of the clinical trial in the individual subject

The duration of the clinical trial for the individual patient is 6 months plus the follow up of 30+10 days. In total a duration of 7 month +10 days is estimated.

3.7.2. Rationale for assessment procedures

Some assessment procedures such as blood tests every 6 months, physical examination, physiotherapeutic evaluation, videography of movement disorder, evaluation of BFMDRS and GMFM-66 and seizure and movement logs are considered clinical routine in the care of patients with GNAO1.

The additional blood sample at visit 2, the CP Child questionnaire, stool samples, visits every 3 months and the scheduled phone calls are not clinical routine but with very limited stress for the patient and the family.

3.7.3. Pharmacokinetics/Determination of drug levels

Serum controls of zinc at visit 0, 2, 3 to measure efficacy of oral zinc administration

3.8. Data quality assurance

3.8.1. General aspects of quality assurance

All investigators involved in this trial are well experienced in the care of patients with GNAO1 related disorders. The assessment tools planned in the trial are regularly used at the site involved, thus a high quality of trial investigations is guaranteed.

The video footage recorded for the BFMDRS will be rated assessor-blinded by an experienced pediatric neurologist otherwise not involved in the care of the trial participants.

With respect to administrative and regulatory issues, the principal investigator will be supported by experienced personnel of the Pediatric Clinical Trials Center Cologne.

3.8.2. Monitoring

On site monitoring will be performed by the Clinical Trials Center Cologne (CTCC) to ensure a high standard of data quality. The objectives of the monitoring procedures are to ensure that the trial subject’s safety and rights as a study participant are respected, that accurate, valid and complete data are collected, and that the trial is conducted in accordance with the trial protocol, the principles of GCP and national and European legislation. Monitoring will be planned according to the ADAMON criteria (TMF project “GCP conform monitoring in Investigator Initiated Trials”; BMBF grant No. 01EZ0876). Source data verification will focus on verification of informed consent, inclusion and exclusion criteria and on the outcome measures. The exact extent of the monitoring procedures is described in a separate monitoring manual.

A monitoring visit report is prepared for each visit describing the progress of the clinical trial and any problems. All tasks of the CTCC will be performed according to SOPs of the CTCC. The principal investigator will reasonable consider the corrective and preventive measures suggested by the monitor.

The investigators agree that the monitor regularly visits the trial site and assure that the monitor will receive appropriate support in their activities at the trial site including access to all necessary documentation. The informed consent (see section 4.5) includes a statement to the effect that the monitor has the right – while observing the provisions of data protection legislation – to compare the case report forms (eCRF) with the trial subject's medical records.

3.8.3. Serious Breaches

Suspected serious breaches may be reported to the sponsor by any investigator, the trial facilities and members of CTCC involved in the trial. The sponsor will assess the suspected serious breach with support by the Pediatric Clinical Trials Center Cologne and will, if the criteria for a serious breach are given, report it into the Clinical Trial Information System (CTIS) respecting the regulatory timelines. Details for processing of serious breaches will be given in a Serious Breaches Manual.

3.8.4. Audits / Inspections

As part of quality assurance, the sponsor has the right to audit the trial site and any other institution involved in the trial. The aim of an audit is to verify the validity, accuracy and completeness of data, to establish the credibility of the clinical trial, and to check whether the trial subject's rights and trial subject safety are being maintained. The sponsor may assign these activities to persons otherwise not involved in the trial (auditors). These persons are allowed access to all trial documentation (especially the trial protocol, case report forms, trial participants' medical records, drug accountability documentation, and trial-related correspondence).

The sponsor and the trial site involved undertake every effort to support auditors and inspections by the competent authorities at all times and to allow the persons charged with these duties access to the necessary original documentation. These persons conducting audits will keep all trial subject data and other trial data confidential.

3.9. Documentation

All data relevant to the trial are documented and signed timely after assessment into the electronic case report form (eCRF). Entering data may be delegated to qualified members of the trial team. The PI is responsible for assuring the data entered into the eCRF is complete, accurate and updates are performed in a timely manner.

The final signature in the eCRF has to be done by the principal investigator.

3.9.1. Data management

Data management activities will be conducted by CTCC.

The IT infrastructure and data management staff will be supplied by the CTCC. The trial database will be developed and validated before data entry based on standard operating procedures at the CTCC. The data management system is based on commercial trial software and stores the data in a database. All changes made to the data are documented in an audit trail. The trial software has a user and role concept that can be adjusted on a trial-specific basis. The database is integrated into a general IT infrastructure and safety concept with a firewall and backup system. The data are backed up daily. After completion and cleaning of data, the database is locked and the data exported for statistical analysis.

The data will be entered remote at the trial site via the internet. Automated plausibility checks are run during data entry, thereby detecting many discrepancies immediately. The data management at CTCC will conduct further checks for completeness and plausibility and will clarify any questions with the trial sites electronically via the trial software. These electronic queries have to be answered by the trial site without unreasonable delay. Further details will be specified in the data management plan. A guidance document for data entry in the eCRF will be provided.

3.9.2. Archiving

All essential trial documents according to ICH-GCP chapter 8 will be archived for at least 25 years after the end of the trial unless longer times are foreseen by local regulatory requirements.

4. Ethical and regulatory aspects

4.1. Approvals by national authorities and ethics committee

Before the start of the clinical trial, all necessary documentation will be submitted to the competent authority (Federal Institute for Drugs and Medical Products, Bundesinstitut für Arzneimittel und Medizinprodukte [BfArM]) and the competent Ethics committee for approval via the Clinical Trials Information System (CTIS).

4.2. Ethical considerations

The present trial protocol and any amendments were and will be prepared in accordance with the principles of the Declaration of Helsinki.

4.3. Legislation and guidelines

The present clinical trial will be conducted in accordance with the principles of the guidelines for Good Clinical Practice (ICH-GCP) and applicable legislation (e.g. German drug law; CTR and GDPR).

These principles cover, amongst other aspects, ethics committee procedures, the obtaining of informed consent from trial participants, adherence to the trial protocol, administrative documentation, documentation regarding the IMP, data collection, trial participants' medical records (source documents), documentation and reporting of adverse events (AEs), preparation for inspections and audits, and the archiving of trial documentation.

All investigators and other staff involved in the trial will be informed that local and national competent authorities as well as foreign supervisory bodies and authorised representatives of the sponsor have the right to review trial documentation and the trial participants' medical records at any time.

4.4. Notification of the authorities and registration

National authorities, ethics committee and local authorities will be notified via CTIS according to applicable regulations.

Before the trial is started, it will be registered under a register approved by the World Health Organisation (WHO) (http://www.who.int/ictrp/en/).

4.5. Obtaining informed consent from trial participants

A patient may not be enrolled into the present trial unless the parents or legal guardian have provided written consent to take part in the trial after being informed verbally and written on the scope and possible medical consequences by a trial investigator. This includes consent to data access by representatives of the sponsor (e.g. monitors or auditors) and the competent authorities. The parents or legal guardian will be informed on potential benefits and possible risks including side effects of the IMP. It must be clear to the parents or legal guardian that withdrawal of consent is possible at any time without giving reasons and without jeopardizing the further medical care of the patient.

Patients with GNAO1 associated disorders are not able to fully understand an informed consent form of this extend and are usually under the supervision of a legal guardian or parents in medical and legal matters, even in adulthood. Thus, informed consent by themselves is not feasible. However, patients will, as far as appropriate for their cognitive development, be informed on the trial, on potential benefits and possible risks and the trial procedures. In the informed consent form, the legal guardians or parents must confirm that they take the child's/participants wishes into account.

The originally signed consent form is archived in the investigator site file at the trial site. Trial participants receive copies of the written information sheet and the signed informed consent form.

The informed consent form and all other documents handed out to the trial subjects and families and any recruitment advertisements will be submitted to the ethics committee for approval before use. Monitors will check that the most recent informed consent form was used before the trial subject’s inclusion and that it was dated and signed by parents/legal guardian.

4.6. Insurance of trial participants

All trial participants enrolled will be insured in accordance with regulatory requirements. In addition, travel accident insurance is provided for patient and both parents/legal guardians.

4.7. Data protection

The provisions of data protection legislation will be observed. It is assured by the sponsor that all investigational materials and data will be pseudonymised in accordance with data protection legislation.

Parents / legal guardians will be informed that the pseudonymised data of the patients will be handled in accordance with applicable law. Participants will not be enrolled into the trial without parents / legal guardians agreeing to data handling as described in the informed consent form.

5. Statistical methods and sample size calculation

5.1. Statistical and analytical plan

All statistical analyses are considered descriptive and, thus, no adjustment will be made for multiple testing. Quantitative variables are summarized by mean, standard deviation and percentiles (0, 25, 50, 75, 100), qualitative variables by count (percentage). P-values are interpreted two-sided. Values below 0.05 are considered statistically significant. Details of the statistical analysis will be fixed in the Statistical Analysis Plan, a separate document to be finalized before database lock.

To allow an analysis of at least 12 evaluable participants, drop-outs will be substituted, however only, if not caused by ability to take the drug or by safety issues.

All enrolled participants will be included in the analyses as far as data are available, but no attempt will be made to impute missing data, if not otherwise specified (see below).

5.1.1. Analysis populations

The planned analyses will be conducted on the following trial populations:

The primary dataset for analysis is the Full Analysis Set (FAS). This dataset includes all trial participants who have been enrolled into the trial with the intention to take (not necessarily taken) the trial medication.

Moreover, analyses are repeated based on the per-protocol population, which is defined by the trial participants which took the scheduled dosage of the IMP at least on 80% of the scheduled days.

The safety population (valid for safety, VAS) includes all trial participants who have been enrolled and have started trial medication and have taken at least one dose.

Data on demographic and baseline characteristics will be evaluated for all analysis populations. The primary analysis of efficacy (first primary endpoint and secondary endpoints) will be performed with the FAS; a secondary analysis of these endpoints will be performed in the PP population. The safety population is the basis for all safety evaluations including adverse events (second primary endpoint) and compliance.

5.1.2. Description of trial subject groups

Demographic and baseline characteristics (e.g. genetic variants, symptoms, age at onset of symptoms, age when first diagnosed, current medication with dosages, current DBS settings) will be tabulated using frequencies, means and quantiles as appropriate.

5.1.3. Primary target variable

The primary objectives of the trial are safety and feasibility of the administration of zinc, thus the primary target variables are defined and analysed as follows:

5.1.3.1. Feasibility

Administration of zinc therapy (total dose, dose per day) will be descriptively summarised. The administration is considered feasible, if zinc was taken in the scheduled dosage at least on 80% of the scheduled treatment days.

5.1.3.2. Safety

Safety is measured by regular evaluation of the AEs as defined in chapter 6.1.

AEs as defined in 6.2.1 are listed and summarized by system organ class, preferred term, intensity, and relatedness.

5.1.4. Secondary and other target variables

- Level of motor-skills
  - as measured by the Gross-Motor-Function messure-66 (GMFM-66) at visit 0, 2 and 3. The level (numerical value) reached at visit 2 and 3 will be compared to the level at baseline.
- Quality of life
  - as reported by the Caregiver Priorities & Child Health Index of Life with Disabilities (CP-Child) Questionnaire for the caregivers (Visit 0, 2 and 3). The level (numerical value) reached at visit 2 and 3 will be compared to the level at baseline.
  - as reported in the Canadian Occupational Performance Measure (COPM) (Visit 0 and 3). The Performance Score (numeric value) and Satisfaction Score (numeric value) reached at visit 3 will be compared to the Score at baseline. The occupational performance problems of all participants will be described in groups.

- Level of movement disorder
    - as measured by Change of Burke-Fahn-Marsden Dystonia Rating scale (BFMDRS). The scale (numerical value) at visit 2 and 3 will be compared to the scale at baseline.
    - as measured by the Abnormal involuntary movement scale (AIMS) . The scale (numerical value) at visit 2 and 3 will be compared to the scale at baseline.
    - as given in the movement log (reported by the parents):
      The AUC of the percentages of involuntary movements, dystonia or choreoathetosis of the awake daytimes during the first two weeks after baseline will be compared to the AUC of the two weeks before visit 2 and to the two weeks before visit 3.
      Missing values will be imputed by the „linear interpolation imputation method“ (*56*). A missing value will be substituted by the mean of the proceeding and the following value.
- Changes in general behaviour including level of alertness
    - As reported by the care givers in the questionnaire. The parents have to classify (classify as e.g. “much improved”; “improved,” “stable”, “declined” or “much declined” or via a rating scale). General behaviour including level of alertness during the first two weeks after baseline will be compared to the two weeks before visit 2 and to the two weeks before visit 3.
- Change in sleep
    - as reported by the parents
      sleep time and sleep interruptions during the first two weeks after baseline will be compared to the two weeks before visit 2 and to the two weeks before visit 3.
- Changes in frequency, time and duration of seizures
    - as reported by the parents
      frequency, time, duration and classification according to ILEA 2017 of seizures during the first two weeks after baseline will be compared to the two weeks before visit 2 and to the two weeks before visit 3.
- Serum level of zinc

- measured at visit 0, 2, 3
  descriptive analyses of the level at baseline, at visit 2 and at visit 3 and of the changes in comparison to baseline

- Serum level of ferritin and copper
  - measured at visit 0, 2, 3
    descriptive analyses of the level at baseline, at visit 2 and at visit 3 and of the changes in comparison to baseline
  - descriptive analyses of the need for ferritin or copper substitution
- Microbiome analysis: descriptive analyses as appropriate

5.1.5. Subgroup analyses

No gender differences are expected, however a subgroup analysis for gender is planned. We expect a male:female ratio of 1:1.

The individual results will be put in correlation to the specific variant of the patient to see if the outcome is influenced by the specific variant if feasible.

No other subgroup analyses are foreseen but may be explored ad hoc.

5.1.6. Interim analysis

No interim analyses are foreseen.

5.2. Sample size calculation

Because there are no data available on the efficacy of treatment with zinc in patients with GNAO1-related disorders, the sample size for this pilot trial was set to 12 participants, a sample size, which seems feasible to be recruited within a limited time frame, but still to allow to answer the question of feasibility of oral zinc treatment and to give first signals on safety.

From a statistical perspective, the sample size n=12 locally minimizes the imprecision of the estimate of probability of response (i.e. the half-width of the exact Clopper-Pearson 95% confidence interval (*57*) assuming a true response probability of 80%, as visible in the chart given below (intersection of yellow curve and broken black line) (calculated with R: A Language and Environment for Statistical Computing, R Foundation for Statistical Computing, Vienna, Austria; binom.test).

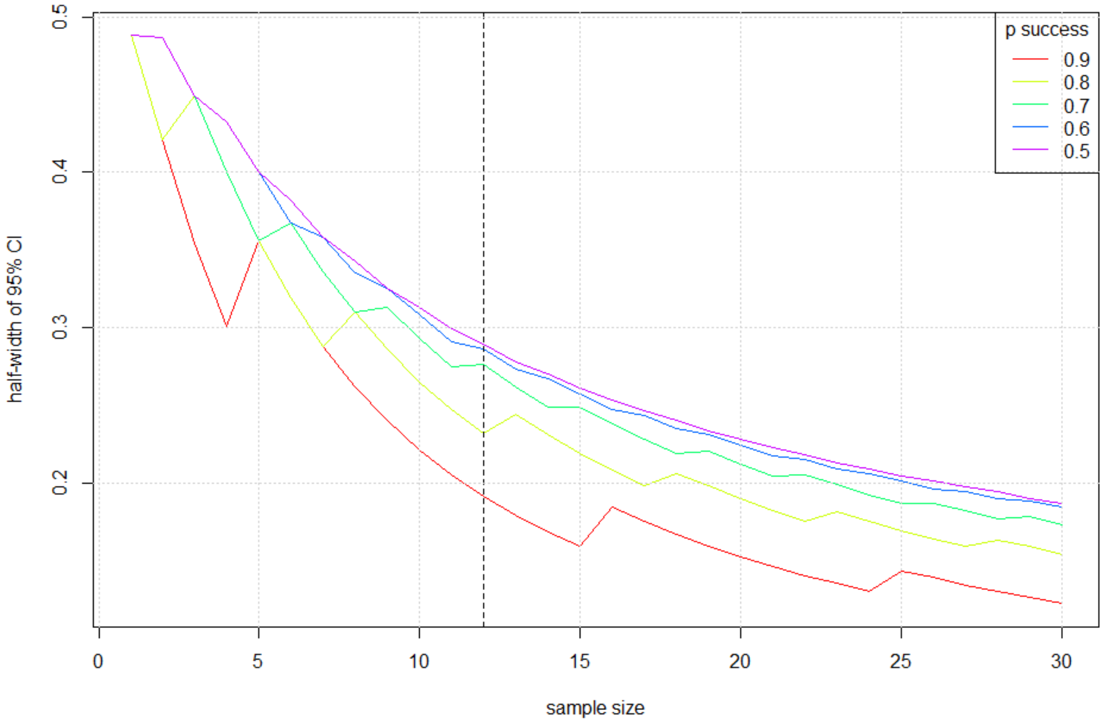

Figure 2: Sample size calculation chart

6. Safety

6.1. Definitions of adverse events and adverse drug reactions

6.1.1. Adverse event

An adverse event (AE) is any untoward medical occurrence in a trial subject administered an IMP. There does not necessarily have to be a causal relationship with this treatment.

6.1.2. Adverse drug reaction

All adverse events judged by either the investigator or the sponsor as having a reasonable suspected causal relationship to an investigational medicinal product qualify as adverse drug reactions (ADR).

6.1.3. Serious adverse events and serious adverse reactions

A serious AE (SAE) or serious ADR (SAR) is any untoward medical occurrence that at any dose

1. Results in death,
2. Is life-threatening at the time of the event,
3. Requires inpatient hospitalisation or prolongation of existing hospitalisation,
4. Results in persistent or significant disability / incapacity,
5. Is a congenital anomaly or birth defect,
6. Is any other medical important event in the opinion of the investigator.

Inpatient hospitalisation is defined as any stay in hospital that includes at least one night (midnight to 06:00). Admission to hospital as an inpatient planned before the first administration of the IMP is not considered as SAE, but must be documented in a proper manner in the trial subject’s medical records.

6.1.4. Expected and unexpected adverse drug reaction

Within the context of this trial, adverse drug reactions listed in the most recent product information (Fachinformation) of the IMP are considered expected. Thus, an unexpected ADR

is an ADR not listed in the product information (Fachinformation) or of which the severity is not consistent with the product information.

Routine health assessment requiring admission for baseline/trending of health status is not considered SAE.

6.1.5. Suspected unexpected serious adverse reactions

A suspected unexpected serious adverse reaction (SUSAR) is an adverse event, the nature or severity of which is not consistent with the product information available for the IMP, is regarded as serious, and has at least a possible causal relationship with the IMP.

6.2. Documentation and follow-up of adverse events

The sponsor ensures that all persons involved in the treatment of trial participants are adequately informed of the responsibilities and actions required when AEs occur. Trial participants or their legal guardians/parents will be asked at each visit at the trial site and during the scheduled phone calls whether they have experienced AEs or SAEs. AEs will be documented in the trial subject’s medical records and in the eCRF. Regardless of whether a causal relationship between the AE and the IMP is suspected, trial participants who develop adverse events must be monitored until all symptoms have been subsided, pathological laboratory values have returned to pre-event levels, a plausible explanation is found for the AE, the trial subject has died, or the study has been terminated for the trial subject concerned.

For the procedure of SAE-reporting see section 6.2.2.

6.2.1. Documentation of adverse events and adverse drug reactions

AEs will be documented if occurring between first intake of IMP and 30 days after last dose of the individual patient resp. until the end of the trial for those participants continuing treatment with Zink after the scheduled end of trial treatment.

AEs are documented in the CRF including the following information:

- AE verbatim
- Date of onset and resolution
- Severity (CTCAE-toxicity grade, see 6.2.2)
- Causal relationship with IMP

- Seriousness
- Action taken
- Outcome

A persistent adverse event is one that extends continuously, without resolution, between subject evaluation time points. Such events should only be recorded once on the Adverse Event eCRF. The initial severity (intensity or grade) of the event will be recorded at the time the event is first reported. If a persistent adverse event becomes more severe, the most extreme severity should be recorded on the Adverse Event eCRF. If an AE becomes serious within the reporting period, it should be reported to the sponsor as described in section 6.3.1.. The SAE information should be amended on the AE eCRF page by providing the date that the event became serious as onset date of the SAE and completing all data fields related to serious adverse event.

6.2.1.1. Exceptions from AE documentation

Temporary worsening of the movement disorder due to simple viral or bacterial infections occurs in many patients with GNAO1 associated disease and is therefore not defined as adverse event within the scope of this trial and will not be documented. However, if the worsening of the movement disorder lasts longer than one month or if no clear infection as trigger can be estimated or if worsening leads to hospitalization (see section 6.3.2), it is defined as adverse event and will be documented as such. Similarly, simple viral or bacterial infections often observed in childhood are not considered adverse events in the context of this trial.

The preexisting symptoms in GNAO1 related disease such as seizures, dystonia and choreoathetosis are subject to a natural variation in daily expression. The preexisting symptoms are not documented as AE.

Preexisting diseases, which are evident before the first administration of the treatment, are not documented as adverse events, however preexisting diseases that worsen during the defined reporting period and any new diseases are documented as AEs.

6.2.2. Severity of the adverse event

The grading of AEs in this trial will be carried out on the basis of the 5-grade scale defined in the Common Terminology Criteria for Adverse Events (CTCAE) version 5 developed by the National Cancer Institute:

Grade 1: Mild; asymptomatic or mild symptoms; clinical or diagnostic observations only; intervention not indicated.

Grade 2: Moderate; minimal, local or noninvasive intervention indicated; limiting age- appropriate instrumental for activities of daily living (ADL).

Grade 3: Severe or medically significant but not immediately life-threatening; hospitalization or prolongation of hospitalization indicated; disabling; limiting self-care ADL.

Grade 4: Life-threatening consequences; urgent intervention indicated.

Grade 5: Death related to AE.

6.2.3. Causal relationship between adverse event and investigational medicinal product

The investigator will assess for every AE whether a causal relationship with the IMP and/or study procedure can be assumed or not. The assessment includes consideration of the nature and type of reaction, the temporal relationship with the IMP, the clinical status of the trial subject, concomitant medication and other relevant clinical factors. If the event is considered due to lack of efficacy or as a symptom or sign of the underlying disorder, no causal relationship will be assumed.

Every AE will be assessed according to the causality determinations of CIOMS VI-Group (Council for International Organizations of Medical Sciences) as follows:

- Related: There is a reasonable possibility that the AE may be related to the IMP
- Not related: There is not a reasonable possibility that the AE may be related to the IMP.

A report on an event which cannot be judged because information is insufficient or contradictory or has not been judged will be regarded as related.

6.3. Reporting of serious adverse events, pregnancy and changes in risk-benefit assessment

Regardless of the assumed causal relationship, every SAE that occurs from the time of IMP administration until 30 days after last dose of the individual patient (resp. until the end of the trial (=Visit 3) for those participants continuing treatment with Zink after the scheduled end of the trial treatment) must be documented in the appropriate part of the eCRF and reported on an SAE form to the sponsor (for exemptions see 6.3.2). Pregnancies must be documented on separate pregnancy forms and reported to the sponsor within the defined periods (see Section 6.3.3.).

6.3.1. Reports from the investigator to the sponsor

The investigator will inform the sponsor of the occurrence or receipt of knowledge of the occurrence of an SAE without delay, at the latest within 24 hours of being made aware by sending the SAE report form, via Fax or Email to Clinical Trials Center Cologne (CTCC, Gleueler Strasse 269, 50935 Cologne) to which the sponsor has delegated the SAE management procedures:

Reporting Dedicated Facsimile: +49 221 478 7984

Reporting E-Mail: zks-sae-vigilanz@uk-koeln.de

6.3.2. Conditions excluded from expedited reporting

In patients with GNAO1 associated disorders, hospitalizations may occur that are clearly related to the natural course of the disease, i.d. deterioration of the neurological condition with uncomplicated infections.

Therefore, in the context of this trial, the following conditions will not be subject to the expedited reporting within 24 h, but will be reported in the eCRF (including days of hospitalisation):

- hospitalization due to neurological deterioration with uncomplicated infections
- seizures in known participants with epilepsy and which proceed in the same way as previously known seizures
- brief, self-limiting worsening of the movement disorder lasting less than 3 hours, or responding to prescribed on-demand medication

However, the following conditions **need** expedited reporting within 24 h, even if considered to be caused by the underlying disease:

- any condition resulting in death or being life-threatening at the time of the event
- hospitalization leading to intensive care treatment
- conditions resulting in persistent or significant disability/incapacity,
- any condition, although per se expected, which in the opinion of the investigator, is unexpectedly severe

6.3.3. Pregnancy

Due to the high grade of disability in GNAO1 and the young age of most of the patients with known GNAO1 variants a pregnancy is extremely unlikely. If pregnancy occurs the investigator will also inform the sponsor without delay within 24 hours of being made aware of any pregnancy of a trial subject that occurs during the trial and its outcome. This will be documented in the pregnancy form part I and II. The pregnant trial subject resp. her legal

guardian will be asked to give separate informed consent for pregnancy follow up. The parents resp. legal guardians will be asked to give informed consent to follow-up of the child until 4 month after life birth.

6.3.4. Obligations and Reporting Requirements of the sponsor

6.3.4.1. Assessment of SAEs by the sponsor

All cases of suspected SAEs are assessed by the sponsor with regard to seriousness (see Section 6.2.2), causal relationship (see Section 6.2.3) and expectedness (see Section 6.1.4).

6.3.4.2. Notification of ethics committee and competent authority

Every SUSAR that becomes known in a clinical trial will be reported by the sponsor to the competent authority and the ethics committee by electronic submission via the Eudravigilance database respecting the regulatory timelines.

6.3.4.3. Review and reporting of changes in the risk-benefit ratio

The sponsor will continuously evaluate any events and review whether these may affect the benefit-risk balance of the clinical trial.

The sponsor will notify the Member States concerned through CTIS of all unexpected events which affect the benefit-risk balance of the clinical trial, but are not suspected unexpected serious adverse reactions. That notification shall be made without undue delay but no later than 15 days from the date the sponsor became aware of this event. This includes, but is not restricted to:

- Individual reports of expected SARs with an unexpected outcome
- A clinically relevant increase in the rate of occurrence of expected SARs
- SUSARs in trial subjects who have already completed the follow-up period of the clinical trial (”end-of-trial visit”)
- Events in connection with the conduct of the study or the development of the investigational medicinal product which may affect the safety of the trial subjects.

6.3.4.4. Informing the investigators

The sponsor or principal investigator will inform investigators of all SUSARs including all relevant further information at the minimum within the periods set by the competent authority. In addition, the sponsor or principal investigator will inform all investigators on changes of the product information (Fachinformation) and on any change of the risk-benefit-ratio.

6.4. Annual safety report (DSUR)

The sponsor will provide annually a report on the safety of trial subjects with all available relevant information concerning patient safety during the reference period to the Member States concerned via CTIS.

The annual safety report will be compiled according to the corresponding ICH guideline E2F „Development Safety Update Report – DSUR“.

7. Trial results and publication

7.1. Reports

7.1.1. Final report

The competent authority and ethics committee will be informed within 90 days after the end of the trial as defined in 3.1.1.

The end of the clinical trial (as defined in 3.1.1) will be communicated within 15 days after the end of the trial through the EU portal.

Within one year from the end of the trial, a summary of the results of the trial will be submitted to the EU database.

7.2. Publication

The trial will be registered in a public register in accordance with the recommendations of the ICMJE (see also Section 4.4).

Trial results will be published in a scientific journal and presented at national or international congresses. Publication of the results of the trial as a whole is intended. Any publication will follow the 'Uniform requirements for manuscripts submitted to biomedical journals (International Committee of Medical Journal Editors' (ICMJE) [JAMA 1997;277(11):927-34]).

Any publication will observe privacy concerning trial participants and investigators.

The following persons and bodies will receive data of this trial.

The data collected in this trial will be shared with the collaborating laboratory in Switzerland in order to interpret the results in the context of the participants.

The data collected will be presented anonymized to caregivers of affected patients in order to improve the knowledge and treatment option.

Changes to this trial protocol may only be implemented if agreed by the sponsor, sponsor's representative, the principal investigator and statistician. Any changes to the trial procedures must be made in writing and must be documented with reasons and signed by the sponsor's representative, the principal investigator and the statistician. Significant changes will be implemented after approval by the competent authority and favorable opinion of the ethics committee, only. Exceptions to this are amendments made to avoid immediate dangers.

8. Literature

a. The European Medicines Agency. Guideline for Good Clinical Practice; (ICH Topic E6(R2)) (EMA/CHMP/ICH/135/1995).

b. The European Medicines Agency. Note for Guidance on Structure and Content of Clinical Study Reports; (ICH Topic E3) (CPMP/ICH/137/95).

c. National Cancer Institute. Protocol Templates, Applications and Guidelines http://ctep.cancer.gov/guidelines/templates.html

d. EMEA-Guideline On Data Monitoring Committees: EMEA/CHMP/EWP/5872/03 Corr

e. The DAMOCLES Study Group. A proposed charter for clinical trial 2005 data monitoring committees: helping them do their job well. Lancet 2005; 365: 711-22

f. Clinical trial registration: a statement from the International Committee of Medical Journal Editors. Accessed on 22 May 2007 at http://www.icmje.org/clin_trial.pdf.

g. WHO - Standardised case causality assessment. http://www.who.int/medicines/areas/quality_safety/safety_efficacy/WHOcausality_assessment.pdf

h. Chan AW, Tetzlaff JM, Gotzsche PC, Altman DG, Mann H, Berlin JA, et al. SPIRIT 2013 explanation and elaboration: guidance for protocols of clinical trials. Bmj. 2013;346:e7586.

i. The European Medicines Agency. Note for Guidance on Statistical Principles for Clinical Trials; (ICH Topic E9) (CPMP/ICH/363/96) http://www.ich.org/products/guidelines/efficacy/efficacy-single/article/statistical-principles-for-clinical-trials.html.

j. Uniform requirements for manuscripts submitted to biomedical journals (International Committee of Medical Journal Editors' (ICMJE) [JAMA 1997;277:927-34].

9. Appendices

9.1. Steering Committee

- PD Dr. Anne Koy, Cologne
- Dr. Moritz Thiel, Cologne
- PD Dr. Kyriakos Martakis, Cologne
- Dr. Vladimir Katanaev, Geneve
- Dr. Barbara Hero, Cologne

9.2. References

1. N. E. I. L. Kulkarni, S. H. A. Tang, R. A. T. A. N. Bhardwaj, S. Bernes, T. A. Grebe, Progressive Movement Disorder in Brothers Carrying a GNAO1 Mutation Responsive to Deep Brain Stimulation. *Journal of child neurology* **31**, 211-214 (2016).
2. K. Nakamura *et al.*, De Novo mutations in GNAO1, encoding a Gαo subunit of heterotrimeric G proteins, cause epileptic encephalopathy. *American journal of human genetics* **93**, 496-505 (2013).
3. T. Schirinzi *et al.*, Phenomenology and clinical course of movement disorder in GNAO1 variants: Results from an analytical review. *Parkinsonism & Related Disorders* **61**, 19-25 (2019).
4. E. Axeen *et al.*, Results of the First GNAO1-Related Neurodevelopmental Disorders Caregiver Survey. *Pediatric Neurology* **121**, 28-32 (2021).
5. H. Feng *et al.*, Mouse models of GNAO1-associated movement disorder: Allele- and sex-specific differences in phenotypes. *PloS one* **14**, (2019).
6. H. S. Singer, J. W. Mink, D. L. Gilbert, J. Jankovic, *Dystonia. In: Movement disorders in childhood*. (Academic Press, London, ed. 2nd, 2015), pp. 177-205.
7. N. M. Allen, J. P. Lin, T. Lynch, M. D. King, Status dystonicus: a practice guide. *Developmental medicine and child neurology* **56**, 105-112 (2014).
8. T. Wirth *et al.*, Highlighting the Dystonic Phenotype Related to GNAO1. *Movement disorders : official journal of the Movement Disorder Society* **37**, 1547-1554 (2022).
9. A. S. Hauser, M. M. Attwood, M. Rask-Andersen, H. B. Schiöth, D. E. Gloriam, Trends in GPCR drug discovery: new agents, targets and indications. *Nature Reviews Drug Discovery* **16**, 829-842 (2017).
10. W. M. Oldham, H. E. Hamm, Heterotrimeric G protein activation by G-protein-coupled receptors. *Nat Rev Mol Cell Biol* **9**, 60-71 (2008).
11. E. M. Ross, T. M. Wilkie, GTPase-activating proteins for heterotrimeric G proteins: regulators of G protein signaling (RGS) and RGS-like proteins. *Annu Rev Biochem* **69**, 795-827 (2000).
12. C. Lin *et al.*, Double suppression of the Galpha protein activity by RGS proteins. *Mol Cell* **53**, 663-671 (2014).
13. K. Nakamura *et al.*, De Novo mutations in GNAO1, encoding a Galphao subunit of heterotrimeric G proteins, cause epileptic encephalopathy. *Am J Hum Genet* **93**, 496-505 (2013).

14. D. Silachev *et al.*, Mouse models characterize GNAO1 encephalopathy as a neurodevelopmental disorder leading to motor anomalies: from a severe G203R to a milder C215Y mutation. *Acta Neuropathol Commun* **10**, 9 (2022).
15. S. Akamine *et al.*, GNAO1 organizes the cytoskeletal remodeling and firing of developing neurons. *The FASEB Journal* **34**, 16601-16621 (2020).
16. H. Feng *et al.*, Movement disorder in GNAO1 encephalopathy associated with gain-of-function mutations. *Neurology* **89**, 762-770 (2017).
17. B. S. Muntean *et al.*, Gαo is a major determinant of cAMP signaling in the pathophysiology of movement disorders. *Cell reports* **34**, 108718-108718 (2021).
18. H. Feng, S. Khalil, R. R. Neubig, C. Sidiropoulos, A mechanistic review on GNAO1-associated movement disorder. *Neurobiology of disease* **116**, 131-141 (2018).
19. R. Carapito *et al.*, A de novo ADCY5 mutation causes early-onset autosomal dominant chorea and dystonia. *Movement disorders : official journal of the Movement Disorder Society* **30**, 423-427 (2015).
20. F. C. F. Chang *et al.*, Phenotypic insights into ADCY5-associated disease. *Movement Disorders* **31**, 1033-1040 (2016).
21. E. Axeen *et al.*, Results of the First GNAO1-Related Neurodevelopmental Disorders Caregiver Survey. *Pediatr Neurol* **121**, 28-32 (2021).
22. A. Benato *et al.*, Long-term effect of subthalamic and pallidal deep brain stimulation for status dystonicus in children with methylmalonic acidemia and GNAO1 mutation. *J Neural Transm (Vienna)* **126**, 739-757 (2019).
23. G. P. Solis *et al.*, Pediatric Encephalopathy: Clinical, Biochemical and Cellular Insights into the Role of Gln52 of GNAO1 and GNAI1 for the Dominant Disease. *Cells* **10**, 2749 (2021).
24. S. E. Mohr, *First in fly : Drosophila research and biological discovery*. (Harvard University Press, Cambridge, Massachusetts, 2018), pp. xii, 257 pages.
25. M. Savitsky, G. P. Solis, M. Kryuchkov, V. L. Katanaev, Humanization of Drosophila Gαo to Model GNAO1 Paediatric Encephalopathies. *Biomedicines* **8**, 395 (2020).
26. Y. Larasati, M. Savitsky, A. Koval, G. Solis, V. Katanaev, Restoration of the GTPase activity of Gαo mutants by Zn2+ in GNAO1 encephalopathy models. (2021).
27. J. R. Bateman, A. M. Lee, C.-t. Wu, Site-Specific Transformation of Drosophila via ϕC31 Integrase-Mediated Cassette Exchange. *Genetics* **173**, 769-777 (2006).
28. T. Schirinzi *et al.*, Phenomenology and clinical course of movement disorder in GNAO1 variants: Results from an analytical review. *Parkinsonism Relat Disord* **61**, 19-25 (2019).
29. H. Saitsu *et al.*, Phenotypic spectrum of GNAO1 variants: epileptic encephalopathy to involuntary movements with severe developmental delay. *Eur J Hum Genet* **24**, 129-134 (2016).
30. R. Arya, C. Spaeth, D. L. Gilbert, J. L. Leach, K. D. Holland, GNAO1-associated epileptic encephalopathy and movement disorders: c.607G>A variant represents a probable mutation hotspot with a distinct phenotype. *Epileptic Disord* **19**, 67-75 (2017).
31. D. C. Schorling *et al.*, Expanding Phenotype of De Novo Mutations in GNAO1: Four New Cases and Review of Literature. *Neuropediatrics* **48**, 371-377 (2017).
32. L. E. M. da Silva *et al.*, Zinc supplementation combined with antidepressant drugs for treatment of patients with depression: a systematic review and meta-analysis. *Nutr Rev* **79**, 1-12 (2021).
33. U. Doboszewska *et al.*, Zinc signaling and epilepsy. *Pharmacology & therapeutics* **193**, 156-177 (2019).
34. A. M. Grabrucker, M. Rowan, C. C. Garner, Brain-Delivery of Zinc-Ions as Potential Treatment for Neurological Diseases: Mini Review. *Drug Deliv Lett* **1**, 13-23 (2011).
35. Y. Cherasse, Y. Urade, Dietary Zinc Acts as a Sleep Modulator. *International Journal of Molecular Sciences* **18**, 2334 (2017).

36. L. P. Brion, R. Heyne, C. S. Lair, Role of zinc in neonatal growth and brain growth: review and scoping review. *Pediatric research* **89**, 1627-1640 (2021).
37. C. Appenzeller-Herzog *et al.*, Comparative effectiveness of common therapies for Wilson disease: A systematic review and meta-analysis of controlled studies. *Liver Int* **39**, 2136-2152 (2019).
38. A. Członkowska *et al.*, Wilson disease. *Nat Rev Dis Primers* **4**, 21 (2018).
39. G. Ranucci, F. Di Dato, M. I. Spagnuolo, P. Vajro, R. Iorio, Zinc monotherapy is effective in Wilson's disease patients with mild liver disease diagnosed in childhood: a retrospective study. *Orphanet J Rare Dis* **9**, 41 (2014).
40. M. Thiel *et al.*, Genotype-phenotype correlation and treatment effects in young patients with GNAO1-associated disorders. *J Neurol Neurosurg Psychiatry*, (2023).
41. Q. Qin, X. Wang, B. Zhou, Functional studies of Drosophilazinc transporters reveal the mechanism for dietary zinc absorption and regulation. *BMC Biology* **11**, 101 (2013).
42. Y. A. Larasati *et al.*, Restoration of the GTPase activity and cellular interactions of Gα(o) mutants by Zn(2+) in GNAO1 encephalopathy models. *Sci Adv* **8**, eabn9350 (2022).
43. E. Aughey, L. Grant, B. L. Furman, W. F. Dryden, The effects of oral zinc supplementation in the mouse. *Journal of Comparative Pathology* **87**, 1-14 (1977).
44. J. Souffriau *et al.*, Zinc inhibits lethal inflammatory shock by preventing microbe-induced interferon signature in intestinal epithelium. *EMBO Mol Med* **12**, e11917 (2020).
45. Y. A. Larasati *et al.*, Zinc for GNAO1 encephalopathy: preclinical profiling and a clinical case. *Research Square*, preprint, DOI 10.21203/rs.21203.rs-3771723/v3771721 (2024).
46. L. A. Anderson, S. L. Hakojarvi, S. K. Boudreaux, Zinc acetate treatment in Wilson's disease. *Ann Pharmacother* **32**, 78-87 (1998).
47. G. J. Brewer *et al.*, Treatment of Wilson's disease with zinc: XV long-term follow-up studies. *J Lab Clin Med* **132**, 264-278 (1998).
48. A. Czlonkowska, J. Gajda, M. Rodo, Effects of long-term treatment in Wilson's disease with D-penicillamine and zinc sulphate. *J Neurol* **243**, 269-273 (1996).
49. C. C. Huang, N. S. Chu, Wilson's disease: resolution of MRI lesions following long-term oral zinc therapy. *Acta Neurol Scand* **93**, 215-218 (1996).
50. G. J. Brewer, R. D. Dick, V. Yuzbasiyan-Gurkan, V. Johnson, Y. Wang, Treatment of Wilson's disease with zinc. XIII: Therapy with zinc in presymptomatic patients from the time of diagnosis. *J Lab Clin Med* **123**, 849-858 (1994).
51. R. Milanino *et al.*, Oral zinc as initial therapy in Wilson's disease: two years of continuous treatment in a 10-year-old child. *Acta Paediatr* **81**, 163-166 (1992).
52. C. Veen, C. J. van den Hamer, P. W. de Leeuw, Zinc sulphate therapy for Wilson's disease after acute deterioration during treatment with low-dose D-penicillamine. *J Intern Med* **229**, 549-552 (1991).
53. T. U. Hoogenraad, J. Van Hattum, C. J. Van den Hamer, Management of Wilson's disease with zinc sulphate. Experience in a series of 27 patients. *J Neurol Sci* **77**, 137-146 (1987).
54. G. J. Brewer, G. M. Hill, A. S. Prasad, Z. T. Cossack, P. Rabbani, Oral zinc therapy for Wilson's disease. *Ann Intern Med* **99**, 314-319 (1983).
55. E. Staub, K. Evers, L. M. Askie, Enteral zinc supplementation for prevention of morbidity and mortality in preterm neonates. *Cochrane Database of Systematic Reviews*, (2021).
56. J. Twisk, W. de Vente, Attrition in longitudinal studies. How to deal with missing data. *J Clin Epidemiol* **55**, 329-337 (2002).
57. C. J. CLOPPER, E. S. PEARSON, THE USE OF CONFIDENCE OR FIDUCIAL LIMITS ILLUSTRATED IN THE CASE OF THE BINOMIAL. *Biometrika* **26**, 404-413 (1934).

9.3. Authors of this Trial Protocol Template:

G Grass; University of Cologne (Sponsor); CTC Cologne

This document is subject to the UVM licence for unprotected content (http://www.ifross.de/Lizenzen/LizenzFuerFreieInhalte.html) and may only be used if the license is adhered to. This document must always be passed on with a copy of the licence. If you use this document as the basis for a trial protocol or when planning a clinical trial, please mention this in the trial protocol as appropriate, or include it in the reference list.
